# Dual Inhibitors of SARS-CoV-2 3CL Protease and Human Cathepsin L Containing Glutamine Isosteres Are Anti-CoV-2 Agents

**DOI:** 10.1021/jacs.4c11620

**Published:** 2025-01-02

**Authors:** Vivek Kumar, Jiyun Zhu, Bala C. Chenna, Zoe A. Hoffpauir, Andrew Rademacher, Ashley M. Rogers, Chien-Te Tseng, Aleksandra Drelich, Sharfa Farzandh, Audrey L. Lamb, Thomas D. Meek

**Affiliations:** † Department of Biochemistry and Biophysics, Texas A&M University, 301 Old Main Drive, College Station, Texas 77845, United States; ‡ Department of Chemistry, University of Texas at San Antonio, 1 UTSA Circle, San Antonio, Texas 78249, United States; § Department of Microbiology & Immunology Centers for Biodefense and Emerging Diseases, The University of Texas Medical Branch at Galveston, 301 University Boulevard, Galveston, Texas 77555, United States

## Abstract

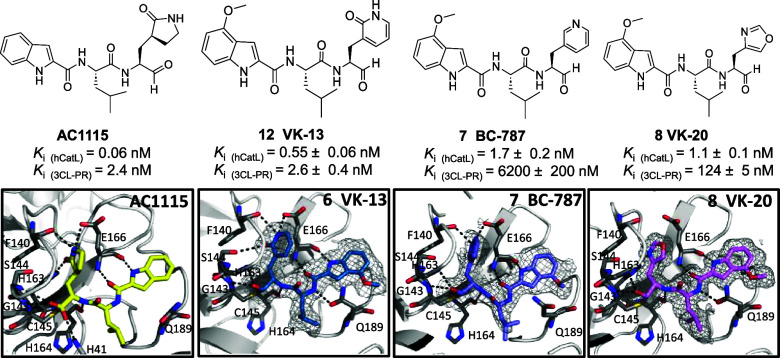

SARS-CoV-2 3CL protease (Main protease) and human cathepsin L are proteases that play unique roles in the infection of human cells by SARS-CoV-2, the causative agent of COVID-19. Both proteases recognize leucine and other hydrophobic amino acids at the P_2_ position of a peptidomimetic inhibitor. At the P_1_ position, cathepsin L accepts many amino acid side chains, with a partial preference for phenylalanine, while 3CL-PR protease has a stringent specificity for glutamine or glutamine analogues. We have designed, synthesized, and evaluated peptidomimetic aldehyde dual-target (dual-acting) inhibitors using two peptide scaffolds based on those of two Pfizer 3CL-PR inhibitors, **Nirmatrelvir**, and **PF-835321**. Our inhibitors contain glutamine isosteres at the P_1_ position, including 2-pyridon-3-yl-alanine, 3-pyridinyl-alanine, and 1,3-oxazo-4-yl-alanine groups. Inhibition constants for these new inhibitors ranged from *K*_i_ = 0.6–18 nM (cathepsin L) and *K*_i_ = 2.6–124 nM (3CL-PR), for which inhibitors with the 2-pyridon-3-yl-alanal substituent were the most potent for 3CL-PR. The anti-CoV-2 activity of these inhibitors ranged from EC_50_ = 0.47–15 μM. X-ray structures of the peptidomimetic aldehyde inhibitors of 3CL-PR with similar scaffolds all demonstrated the formation of thiohemiacetals with Cys_145_, and hydrogen-bonding interactions with the heteroatoms of the pyridon-3-yl-alanyl group, as well as the nitrogen of the N-terminal indole and its appended carbonyl group at the P_3_ position. The absence of these hydrogen bonds for the inhibitors containing the 3-pyridinyl-alanyl and 1,3-oxazo-4-yl-alanyl groups was reflected in the less potent inhibition of the inhibitors with 3CL-PR. In summary, our studies demonstrate the value of a second generation of cysteine protease inhibitors that comprise a single agent that acts on both human cathepsin L and SARS-CoV-2 3CL protease. Such dual-target inhibitors will provide anti-COVID-19 drugs that remain active despite the development of resistance due to mutation of the viral protease. Such dual-target inhibitors are more likely to remain useful therapeutics despite the emergence of inactivating mutations in the viral protease because the human cathepsin L will not develop resistance. This particular dual-target approach is innovative since one of the targets is viral (3CL-PR) required for viral protein maturation and the other is human (hCatL) which enables viral infection.

## Introduction

**Figure 1:**
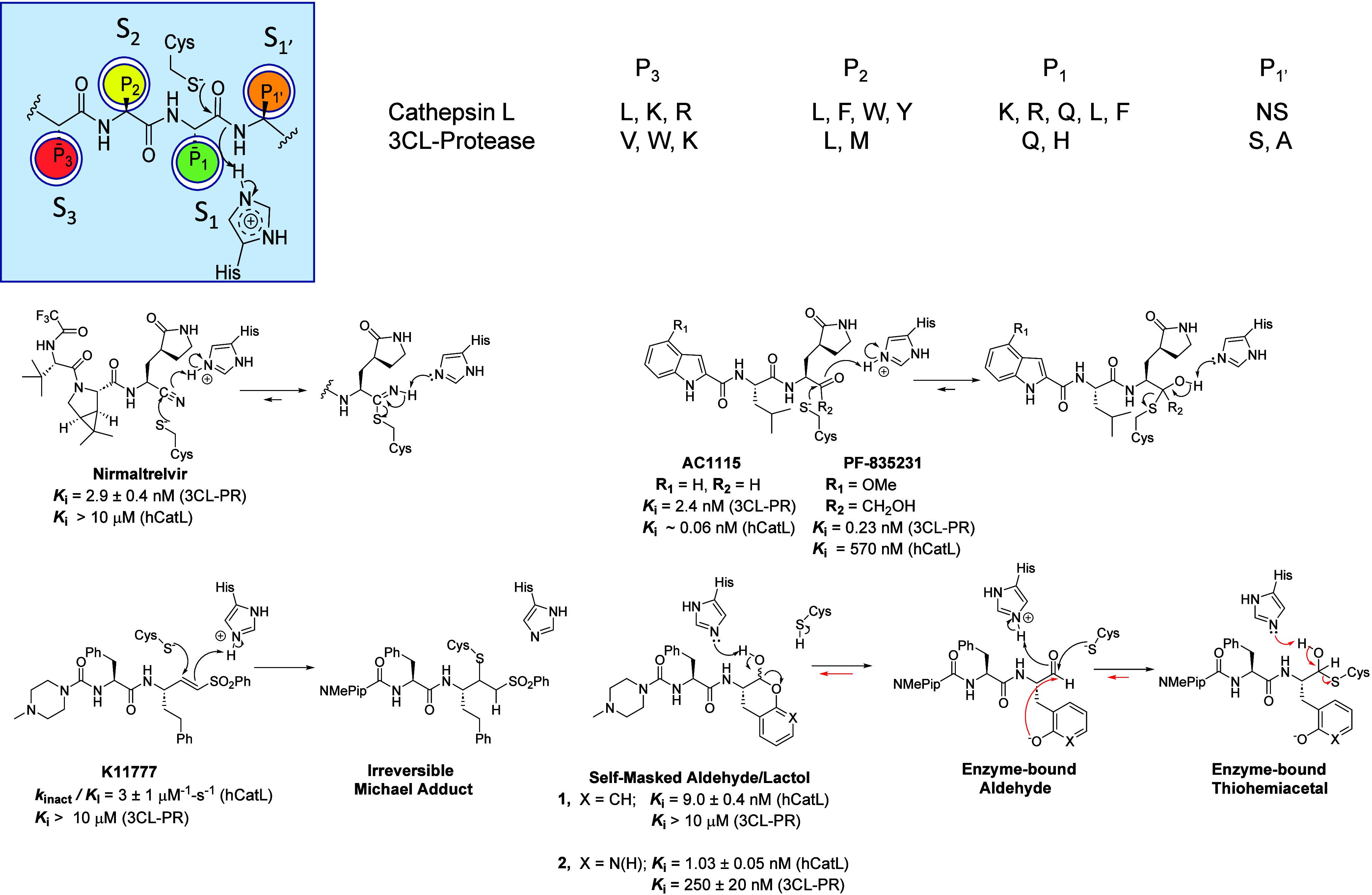
(top) Substrate specificities of cathepsin L and 3CL protease for the P_3_–P_1__′_^23^ side chains with amino acids named in single-letter code. NS; nonspecific. The most commonly found amino acids in cleavage sites of the two proteases are listed left to right in terms of frequency.^24,25^ (middle) Structure of **Nirmatrelvir**, inhibition constants vs 3CL-PR and hCatL, and putative mechanism of reversible covalent inhibition of 3CL-PR.^8^ Structures of **AC1115**(26) and **PF-835231**,^27^ inhibition constants vs 3CL-PR and hCatL, and putative mechanisms of reversible covalent inhibition of the proteases. (bottom) Structure of **K11777**, second-order rate constant of inactivation of hCatL,^19^ and proposed mechanism of irreversible Michael addition to target cysteine proteases.^28^ Self-masked aldehyde **1**, inhibition constants for hCatL and 3CL-PR, and the demonstrated mechanism of reversible covalent inhibition of cruzain.^29^ Compound **2**, the 2-pyridon-3-yl-Ala analogue of **1** shown with inhibition constants for hCatL^30^ and 3CL-PR.^29^

The global health crisis caused by the COVID-19 outbreak of 2019^1^ is the fifth most deadly in human history.^2^ According to the World Health Organization, as of January 2024, there have been 774 million cases and 6.9 million deaths of COVID-19-related deaths worldwide.^3^ Since 2020, the approval and availability of vaccines resulted in the downgrading of COVID-19 by the WHO from pandemic to endemic status; however, new strains of SARS-CoV-2 continue to emerge,^4^ requiring continuous genetic modifications in existing vaccines to retain efficacy. COVID-19 cases spiked upward in late 2023, resulting in an 8% increase in COVID-19-related hospitalizations and deaths in the United States.^5^ Paxlovid, the single approved therapy for COVID-19, remains the standard of care today.^6,7^ Paxlovid contains two drugs: nirmatrelvir,^8,9^ a reversible covalent inhibitor of the cysteine protease SARS-CoV-2 3-chymotrypsin-like (3CL-PR, or Main) protease^10,11^ (Figure 1) and ritonavir, an inhibitor of HIV protease which is an additive to prevent oxidation of nirmatrelvir by human cytochrome 3A4 (CYP3A4).^12^ Despite the FDA approval of Paxlovid, the discovery of new drugs to treat COVID-19 still remains essential for this disease. Paxlovid is contra-indicated in patients who take medications that rely on CYP3A4 for drug metabolism and where induction of CYP3A4 will result in inactivation of other drugs.^13^ Recently, mutations within the substrate-binding site of 3CL-PR in emergent strains of SARS-CoV-2 result in poorer binding of nirmatrelvir, such that Paxlovid may ultimately become ineffective.^14,15^ Accordingly, the discovery of new inhibitors of SARS-CoV-2 3CL-PR remains a necessity.

Beta-coronaviruses encode two major open-reading frames that are translated into two polyproteins, pp1a and pp1ab, with the second polyprotein arising from a frameshift that allows translation of a second internal open-reading frame.^16,17^ Two nascent cysteine proteases (CPs) are expressed within both polyproteins: 3CL-PR (also called Main protease) and the papain-like (PL-PR)^18^ protease. These coronaviral CPs are essential for viral infection. 3CL-PR and papain-like protease catalyze cleavages in the polyproteins at 11 and 4 peptide sequences, respectively. The cleavage of the polyproteins affords maturation of the nonstructural proteins, including the essential enzymes for infectious viruses. Two 3CL-PR domains within pp1a(b) form a homodimer, leading to autocatalysis to release active 3CL-PR from the polyproteins, and subsequent catalysis of other peptide cleavages resulting in the formation of structural and nonstructural proteins of the coronavirus. The mature 3CL-PR (EC 3.4.22.69; Uniprot number P0CTC1) homodimer is composed of 34 kDa subunits. Each monomer harbors an active site with a Cys_145_-His_41_ catalytic dyad, which is a hallmark of cysteine hydrolases.

We recently showed that **K11777** is an irreversible covalent inactivator of cysteine proteases including, human cathepsin L (hCatL), and blocked SARS-CoV-2 infection of permissive mammalian cells (cells that can enable a productive infection of SARS-CoV-2 that yield progeny virus) at nanomolar concentrations, but was completely inactive as an inhibitor of CoV-2 3CL-PR (Figure 1).^19^ The study showed that (a) hCatL was the cellular target of **K11777**, and (b) anticoronaviral action of **K11777** results from inhibiting cleavage of the Spike protein of the coronavirus by hCatL, a crucial step in the disassembly of the virus inside of host cells.^19^ hCatL is an important host cell factor for infection by SARS-CoV-2, in some but not all SARS-CoV-2 permissive cells.^20,21^ In addition, expression of hCatL has been shown to be upregulated in lung tissue collected from autopsy samples of COVID-19 patients,^22^ and the level of upregulation correlates with the extent of lung damage. Accordingly, hCatL has roles in both the initiation of SARS-CoV-2 infection and in pulmonary pathology.

As with all cysteine proteases, the active sites of 3CL-PR or hCatL are composed of substrate subsites (S_3_, S_2_, S_1_, S_1′_, etc.) which accommodate the corresponding side chains of peptide substrates and peptidomimetic inhibitors (P_3_, P_2_, P_1_, P_1′_, etc.), for which the peptide cleavage site occurs between the P_1_–P_1__′_ residues of substrates.^23^ The P_2_, P_1_, and P_1′_ peptide sequences recognized by 3CL-PR, (**L**/V/F/M)-(**Q**/H)*-(**G**/A/S/N), have cleavage sites (*) following a Gln at the P_1_ residue, which, with the exception of His, is otherwise invariant (Figure 1).^24^ While the substrate specificities of 3CL-PR and cathepsin L^25^ differ in their preference for the P_1_ side chain, wherein the former protease has high specificity for Gln while the latter enzyme is nonspecific, both proteases have high specificity for Leu at the P_2_ position (Figure 1).

These cumulative findings suggest that a single compound that is a dual-target inhibitor of both SARS-CoV-2 3CL-PR and hCatL would provide a powerful treatment alternative to the single-target agent nirmatrelvir. **PF-835231**(27) and **AC1115**(26) (Figure 1) contain an activated ketone or an aldehyde warhead, respectively, within nearly identical peptidomimetic scaffolds. Both inhibitors are administered as prodrugs and are clinically successful anti-COVID-19 agents. **AC1115** has previously been shown to be a dual target inhibitor of 3CL-PR and hCatL,^26^ and we find here that **PF-835231**(27) is as well, albeit, with poor inhibition of hCatL.

Of current fashion in medicinal chemistry is the development of covalent inhibitors that act reversibly against their enzyme targets to foster sufficient selectivity without the potential toxicity of irreversible covalent inactivation.^31,32^ While peptide aldehyde inhibitors comprise a long-standing class of reversible covalent inhibitors of cysteine protease,^33−35^ they are highly reactive electrophiles with significant metabolic liabilities. We have recently reported a new class of cysteine protease inhibitors, the self-masked aldehyde inhibitors (SMAIs), in which a 2-hydroxyphenylalanyl group in the P_1_ position of the inhibitors forms a δ-lactol with the C-terminal aldehyde (Compound **1**, Figure 1).^29,30^ Compound **1** has a similar peptide scaffold as **K11777**. We demonstrated that **1** remains in its δ-lactol form in an aqueous solution but upon binding to the CP cruzain (and presumably, hCatL) the δ-lactol is opened,^29^ and the resulting aldehyde forms a reversible thiohemiacetal with the protease, effecting low nanomolar inhibition of cruzain and hCatL overall.^29,30^ While the *o*-hydroxy-Phe group at P_1_ is well accommodated by cruzain and hCatL, it does not sufficiently resemble a glutamine residue to effect inhibition of 3CL-PR. However, the single atomic change of carbon to a nitrogen atom to provide the P_1_ 2-pyridon-3-alanyl group in **2** resulted in a dual-target, reversible inhibitor of both proteases, albeit with an opportunity for improvement for 3CL-PR by modification of the peptide scaffold. These findings demonstrate that the 2-pyridon-3-alanyl group is a “chimera” of a Phe or Gln side chain at the P_1_ position and suggest that derivatization of this 2-pyridon-3-alanal amino acid analogue and optimization of its peptidomimetic scaffold will afford potent, reversible covalent inhibitors of both 3CL-PR and hCatL with more durable anti-SARS-CoV-2 activity. Here, we further explore this side chain and other isosteres of glutamine in a series of peptide aldehyde inhibitors, characterize their modes of inhibition and activities in SARS-CoV-2 infected cells, and elucidation their binding interactions with 3CL-PR by X-ray crystallography.

## Experimental Section

### Chemicals

Sodium acetate, disodium–EDTA, Tris–HCl, and NaCl were obtained from Millipore Sigma. CHAPS and dithiothreitol were obtained from BioGold. Cbz-Phe-Arg-7-amino-4-methylcoumarin (Z-FR-AMC) was purchased from EMD Millipore or GenScript. The methods for the synthesis and characterization of the FRET-based substrate for 3CL-PR, (Abz)HN-Ser-Ala-Val-Leu-Gln*Ser-Gly-Phe-Arg-Lys(e-Dnp)-CONH_2_ are described in Mellott et al.^19^

### Chemical Synthesis

#### General Considerations of Synthesis

Chemicals and reagents were purchased from commercial sources and used as received without further purification. Reactions were carried out under an inert atmosphere of nitrogen unless otherwise specified. The progress of the reactions was monitored using thin layer chromatography (TLC) and LC-MS (liquid chromatography-mass spectrometry) analysis, by employing an HPLC-MS (UltiMate 3000 equipped with a diode array spectrophotometer coupled to an MSQ Plus Single Quadrupole Mass Spectrometer, ThermoFisher Scientific), using electrospray positive and negative ionization detection. Conditions used for HPLC were: Column: Phenomenex Luna 5 μm C18(2) 100 Å, 4.6 mm × 50 mm, Mobile phase A: water with 0.1% formic acid (v/v). Mobile phase B was acetonitrile with 0.1% formic acid (v/v). Temperature: 25 °C. Gradient: 0–100% B over 6 min, then a 2 min hold at 100% B. Flow rate: 1 mL/min. Spectrophotometric detection: 254 nm.

Compounds were purified by flash column chromatography (FCC) on silica gel (200–300 mesh) with different solvent systems. Most of the final compounds were purified by semipreparative HPLC (Prep-HPLC) on the same UltiMate 3000 HPLC system, which was connected to a fraction collector. The typical settings for preparative HPLC were as follows: Column used: Phenomenex Luna 5 μm C18(2) 100 Å, 21.2 mm × 250 mm; mobile phases A and B were the same as those for analytical HPLC; method: gradient elution at 10–100% B over 25 min, then isocratic elution at 100% B for 5 min; flow rate: 21.2 mL/min. Proton NMR spectra were obtained in DMSO-*d*_*6*_ or CDCl_3_ at 400 MHz at 298 K on a Bruker Avance III NanoBay console with an Ascend magnet. The following abbreviations were utilized to describe peak patterns when appropriate: br = broad, s = singlet, d = doublet, q = quartet, t = triplet, and m = multiplet. The compound used for testing in assays and biological studies had purities that were determined to be >95% as evaluated by their proton NMR spectra and their HPLC/MS based on ultraviolet detection at 254 nm.

The syntheses of compounds **1**(29) and **2**(36) are described elsewhere, as well as their inhibition data. The synthesis, kinetics, and activity in infected cells of **K11777**(19) and compound **3**(29) are reported elsewhere.

**Scheme 1:**
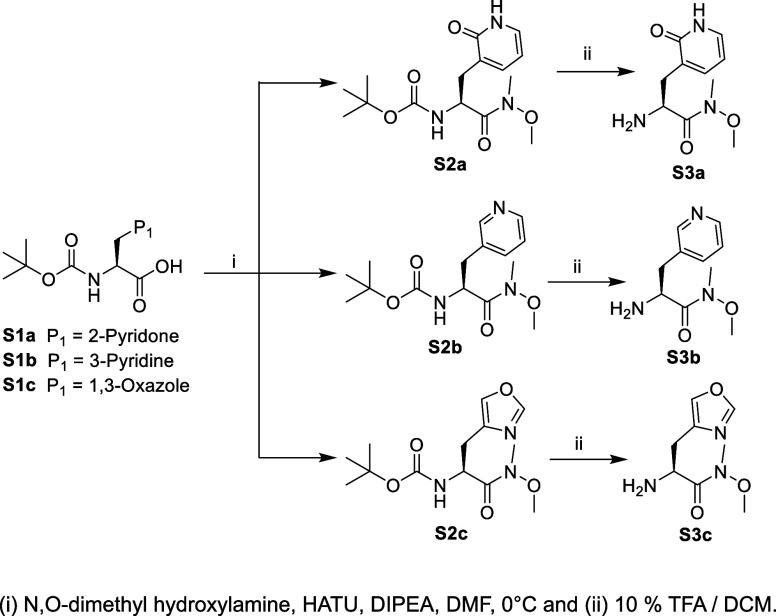
Synthesis of Compounds **S3a**, **S3b**, and **S3c**

**Scheme 2:**
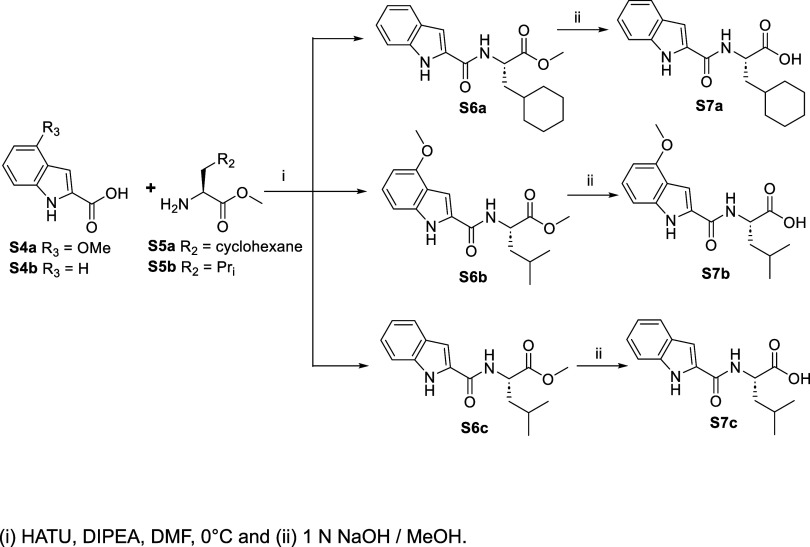
Synthesis of **S7a**, **S7b**, and **S7c**

**Scheme 3:**
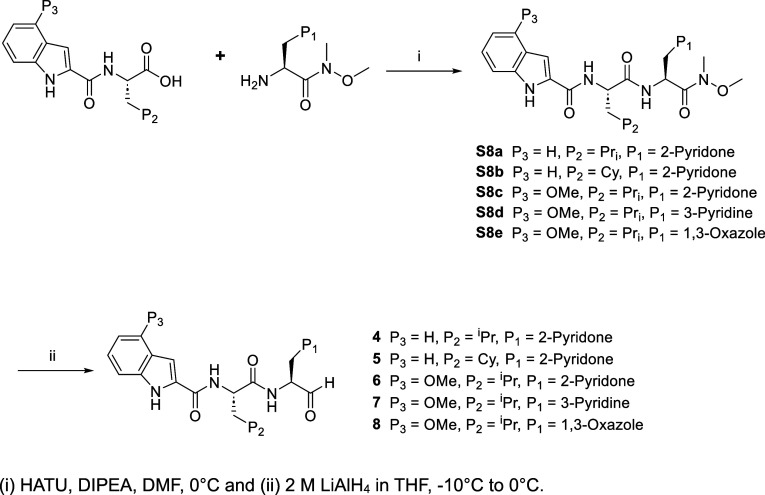
Synthesis of Compounds **4–8**

**Scheme 4:**
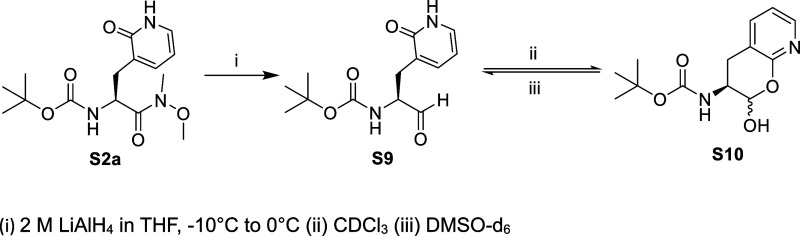
Synthesis of Compound **S9**

Synthesis of compounds **4–8**. Characterization and detailed procedures for all reactions in Schemes 1–4 are described below. Compounds **S1b** and **S1c** were purchased commercially from Ambeed. Compound **S1a** was synthesized using a published protocol.^29^

##### *tert*-Butyl (*S*)-(1-(Methoxy(methyl)amino)-1-oxo-3-(2-oxo-1,2-dihydropyridin-3-yl)propan-2-yl)carbamate (S2a, P_1_ = 2-Pyridone)

In a 25 mL round-bottom flask, **S1a** (3 mmol, 1 equiv, 847 mg) was dissolved in 5 mL of DMF and cooled to 0 °C. To this solution, HATU (3.6 mmol, 1.2 equiv, 1369 mg) was added. DIPEA (6 mmol, 6 equiv, 1 mL) was added, and the reaction was stirred for 2 min at 0 °C. N,O-Dimethyl hydroxylamine hydrochloride (4.5 mmol, 1.5 equiv, 441 mg) was neutralized with DIPEA, dissolved in 1 mL of DMF, and added slowly to the reaction mixture, which was stirred for an additional 24 h. After reaction completion, the mixture was added to 100 mL of water, followed by 100 mL EtOAc. The product was extracted three times with EtOAc (3 × 100 mL). The organic layer was washed three times with a 0.1 N HCl solution (3 × 50 mL), three times with a 10% Na_2_CO_3_ solution (3 × 50 mL), and finally washed with brine (3 × 50 mL). The organic layer was dried over Na_2_SO_4_. The product compound was purified by silica gel column chromatography using an EtOAc/hexane solvent system (30–50% EtOAc in hexane, v/v) to give **S2a** (yield 660 mg, 70%). ^1^H NMR (400 MHz, DMSO-D_6_) δ 1.30 (s, 9H, Boc), 2.56 (dd, *J* = 4, 12 Hz, 1H, 2-Pyrd-CH_2_), 2.73 (d, *J* = 8 Hz, 1H, 2-Pyrd-CH_2_), 3.13 (s, 3H, amide N–CH_3_), 3.70 (s, 3H, N–O–CH_3_), 4.68 (s broad, 1H, CH_α_), 6.11 (t, *J* = 4 Hz, 1H, 2-Pyrd C–H), 7.04 (d, *J* = 8 Hz, 1H, Boc-N–H), 7.25 (d, *J* = 4 Hz, 2H, 2-Pyrd C–H), 11.55 (s, 1H, 2-Pyrd N–H). ^13^C NMR (100 MHz, DMSO-D_6_) δ 1.38 (28.64, 32.71, 50.42, 53.94, 78.40, 105.30, 128.60, 134.01, 139.81, 155.63, 163.32). ^1^H NMR (400 MHz, CDCl_3_) δ 1.38 (s, 9H, Boc), 2.90 (d, *J* = 4 Hz, 2H, 2-hydroxy-pyridyl CH_2_), 3.19 (s, 3H, amide N–CH_3_), 3.81 (s, 3H, N–O–CH_3_), 4.92 (q, *J* = 4,8 Hz, 1H, CH_α_), 6.03 (s, 1H, Boc-N–H), 6.21 (t, *J* = 8 Hz, 1H, 2-hydroxy-pyridine CH), 7.31 (d, *J* = 8 Hz, 2H, 2-hydroxy-pyridine CH), 12.92 (s, broad, 1H, hydroxy-pyridine O–H). ^13^C NMR (100 MHz, CDCl_3_) δ (28.34, 32.95, 51.08, 53.71, 61.67, 79.27, 106.63, 128.43, 133.28, 140.41, 155.52, 165.27, 172.51). (LC-MS: *t*_R_ = 3.26 min; C_15_H_23_N_3_O_5_ (*m*/*z* calcd 325.16, found 326.2 (M + H), 226.2 (M-Boc)). Similarly, **S2b** and **S2c** were prepared. For **S2b:** (LC-MS: *t*_R_ = 2.66 min; C_15_H_23_N_3_O_4_ (*m*/*z* calcd 309.17, found 310.1 (M + H), 209.2 (M-Boc). **S2c** (LC-MS: *t*_R_ = 3.01 min; C_13_H_21_N_3_O_5_ (*m*/*z* calcd 299.2, found 300.2 (M + H), 199.1 (M-Boc).

For Boc deprotection, **S2a** (2 mmol, 2 equiv, 650 mg) was dissolved in 10% (v/v) TFA/DCM (1 mL of TFA in 9 mL of DCM) in a 250 mL round-bottom flask and stirred for 2 h. After completion of the reaction, excess DCM was added to the mixture, followed by evaporation under vacuum to give **S3a**. TFA was removed by evaporation to provide the gummy product, which was dissolved in 5 mL of water and lyophilized to afford the TFA salt of **S3a**, which was used for further steps without purification. Compounds **S3b** and **S3c** were prepared in a similar manner.

##### (4-Methoxy-1*H*-indole-2-carbonyl)-l-leucine (**S7b**, P_2_ = Pr_i_, P_3_ = OMe)

In a 25 mL round-bottom flask, **S4a** (5 mmol, 5 equiv, 955 mg) and HATU (6 mmol, 1.2 equiv, 2280 mg) were dissolved in 5 mL DMF and cooled to 0 °C. To this solution, DIPEA (10 mmol, 2 equiv, 1.6 mL) was added slowly with stirring for 2 min to form the activated HOAt ester. l-Leu methyl ester (HCl salt) **S5b** (6 mmol, 1.2 equiv, 786 mg) was neutralized with DIPEA, and the mixture was dissolved in 1 mL of DMF. This solution was added slowly to the reaction mixture followed by stirring overnight. After completion of the reaction, 100 mL of water was added to the reaction mixture, followed by the addition of 100 mL EtOAc. The product compound was extracted with EtOAc (3 × 100 mL). The organic layer was washed three times with a 0.1 N HCl solution (3 × 50 mL), three times with a 10% (w/v) Na_2_CO_3_ solution (3 × 50 mL), and finally washed with brine (3 × 50 mL). The organic layer was dried over Na_2_SO_4_. The product was purified by column chromatography (silica gel) using a EtOAc/hexane solvent system (20–30% EtOAc in hexane, (v/v)). The solvent was evaporated to give a white powder (**S6b**); yield 1.05 g, 70%). In a 250 mL round-bottom flask, the whole compound **S6b** (1.05 g, 3.3 mmol) was dissolved in 25 mL of MeOH. Twenty-five mL of 1 N NaOH in water was added slowly to the reaction mixture, followed by stirring for 2 to 3 h. After complete hydrolysis as confirmed by TLC and LC-MS, MeOH was evaporated, and the reaction mixture was acidified with 1 N HCl solution. The compound was extracted three times with EtOAc (3 × 100 mL) and washed three times with brine (3 × 50 mL). EtOAc was evaporated to give a pure white powder (**S7b**; 915 mg, 91%). (LC-MS: *t*_R_ = 4.52 min; C_16_H_20_N_2_O_4_ (*m*/*z*: calcd 304.1; found 305.1 (M + H)). Similarly, intermediates **S7a** and **S7c** were prepared. **S7a**, LC-MS: *t*_R_ = 4.52 min; C_18_H_22_N_2_O_3_ (*m*/*z*: calcd 314.2; found 315.2 (M + H)). **S7c**, LC-MS: *t*_R_ = 4.73 min; C_15_H_18_N_2_O_3_ (*m*/*z*: calcd 274.1; found 275.1 (M + H), 297.1 (M + Na)).

#### Synthesis of Compounds **4–8**

In a 25 mL round-bottom flask, **S7b** (2 mmol, 1 equiv, 610 mg) and HATU (2.4 mmol, 1.2 equiv, 912 mg) were dissolved in 5 mL of DMF and cooled to 0 °C. The acid was converted to its HOAt ester by adding DIPEA (4 mmol, 2 equiv, 0.7 mL) into the reaction mixture, followed by stirring for 2–3 min. The TFA salt of **S3a** (2.4 mmol, 1.2 equiv, 813 mg) was neutralized with a few drops of DIPEA and then dissolved into 1 mL DMF. The **S3a** solution was added dropwise into a reaction mixture with the activated ester at 0 °C and stirred for 24 h. After completion of the reaction, the mixture was added to 100 mL of water, followed by the addition of 100 mL of EtOAc. The product was extracted with EtOAc (3 × 100 mL). The organic layer was washed with 0.1 N HCl solution (3 × 50 mL), three times with 10% Na_2_CO_3_ solution (50 mL s), and finally washed with brine (3 × 50 mL s). The organic layer was dried over Na_2_SO_4_. The compound was purified by silica gel column chromatography using a methanol/DCM solvent system (4–6% methanol in DCM, v/v). The solvent was evaporated to give a yellowish gummy compound **S8c** (511 mg, 50%) (LC-MS: *t*_R_ = 4.05 min; C_26_H_33_N_5_O_6_, *m*/*z*: calcd 511.2; found 512.2 (M + H)). Similarly, **S8a**, **S8b**, **S8d**, and **S8e** were prepared. **S8a**, LC-MS: *t*_R_ = 4.27 min; C_25_H_31_N_5_O_5_, *m*/*z*: calcd 481.2; found 482.3 (M + H). **S8b**, LC-MS: *t*_R_ = 4.81 min; C_28_H_35_N_5_O_5_, *m*/*z*: calcd 521.2; found 522.2 (M + H). **S8d**, LC-MS: *t*_R_ = 3.63 min; C_26_H_33_N_5_O_5_, *m*/*z*: calcd 495.2; found 496.2 (M + H). **S8e**, LC-MS: *t*_R_ = 4.42 min; C_24_H_31_N_5_O_6_, *m*/*z*: calcd 485.2; found 486.2 (M + H).

In a 100 mL round-bottom flask, **S8c** (1 mmol, 1 equiv, 511 mg) was dissolved in 10 mL of dry THF and cooled to −10 °C. Under nitrogen, 2 M LiAlH_4_ in THF (1.2 mmol, 1.2 equiv, 550 μL) was added dropwise into the reaction mixture. The reaction was stirred for 30–40 min. After completion of the reaction, it was slowly quenched with a few drops of 0.1 N HCl solution at 0 °C. THF was evaporated, and excess water was added to the mixture. The aldehyde was extracted with EtOAc (3 × 50 mL). The organic layer was washed with brine solution (3 × 50 mL). The organic layer was concentrated under reduced pressure and dissolved in 5 mL of DMF. Compound **6** was purified by preparative HPLC using a linear gradient of (40–60% acetonitrile) in 0.1% formic acid (v/v) (yield: 60 mg, 13%). Due to severe peak tailing of aldehydes observed upon purification with preparative HPLC, only the peaks corresponding to purity >95% were collected, which were then lyophilized. Compound **6**; LC-MS: *t*_R_= 3.7 min: C_24_H_28_N_4_O_4_, *m*/*z*: calcd, 452.21; found, 453.24 (M + H). ^1^H NMR (400 MHz, DMSO-D_6_) δ 0.86 (m, 6H), 1.50 (tq, 1H), 1.63 (m, 2H), 2.58 (ddd, 1H), 3.00 (dd, 1H), 3.89 (s, 3H), 4.36 (m, 1H), 4.50 (ddt, 1H), 6.02 (dt, 1H), 6.51 (d, 1H), 7.02 (d, 1H), 7.10 (t, 1H), 7.19 (ddd, 1H), 7.24 (dd, 1H), 7.32 (m, 1H), 8.36 (dd, 1H), 8.43 (dd, 1H), 9.46 (d, 1H), 11.52 (s, 1H), 11.55 (s, 1H).

Compound **4** (yield: 40 mg, 10%), LC-MS: *t*_R_= 4.11 min, C_23_H_26_N_4_O_4_; *m*/*z*: calcd, 422.2; found, 423.2 (M + H). ^1^H NMR (400 MHz, DMSO-D_6_) δ: 0.87 (m, 6H), 1.49 (m, 3H), 3.17 (s, 2H), 3.25 (m, 2H), 3.39 (d, 1H), 4.04 (d, 2H), 4.34 (s, 1H), 4.47 (tdd, 1H), 4.53 (s, 1H), 5.96 (m, 1H), 7.03 (tt, 1H), 7.20 (m, 3H), 7.43 (dt, 1H), 7.60 (m, 1H), 8.30 (q, 1H), 9.47 (d, 1H), 11.49 (s, 1H), 11.54 (s, 1H).

Compound **5** (yield: 70 mg, 15%), LC-MS: *t*_R_= 4.63 min, C_26_H_30_N_4_O_4_; *m*/*z*: calcd, 462.2; found, 463.3 (M + H). ^1^H NMR (400 MHz, DMSO-D_6_) δ 0.83 (dtd, 2H), 1.06 (p, 3H), 1.35 (m, 1H), 1.55 (m, 6H), 1.66 (d, 1H), 2.52 (ddd, 1H), 2.92 (dd, 1H), 4.28 (dddd, 1H), 4.50 (ddd, 1H), 5.91 (dt, 1H), 6.97 (t, 1H), 7.12 (m, 5H), 7.37 (d, 1H), 7.55 (d, 1H), 8.39 (m, 1H), 9.39 (d, 1H), 11.49 (s, 1H), 11.51 (s, 1H).

Compound **7** (yield: 100 mg, 24%), LC-MS: *t*_R_ = 3.52 min, C_24_H_28_N_4_O_4_; *m*/*z*: calcd, 436.2; found, 437.3 (M + H). ^1^H NMR (400 MHz, DMSO-D_6_) δ: 0.85 (ddq, 6H), 1.37 (h, 1H), 1.54 (dd, 2H), 2.66 (q, 1H), 2.82 (ddd, 1H), 3.22 (m, 1H) 3.88 (s, 3H), 4.37 (td, 1H), 4.48 (s, 1H), 6.51 (dt, 1H), 7.02 (dd, 1H), 7.12 (m, 2H), 7.34 (d, 1H), 7.56 (m, 1H), 7.63 (d, 1H), 8.26 (m, 1H), 8.42 (m, 1H), 9.49 (d, 1H), 11.54 (s, 1H).

Compound **8** (yield: 20 mg, 5%), LC-MS: *t*_R_ = 4.22 min, C_22_H_26_N_4_O_5_; *m*/*z*: calcd, 426.2; found, 427.2 (M + H). ^1^H NMR (400 MHz, DMSO-D_6_) δ 0.80 (m, 6H), 1.42 (qq, 1H), 1.58 (dd, 2H), 2.74 (m, 1H), 2.95 (ddd, 1H), 3.82 (s, 3H), 4.27 (ddd, 1H), 4.44 (m, 1H), 6.44 (d, 1H), 6.94 (d, 1H), 7.03 (dd, 1H), 7.26 (dd, 1H), 7.76 (d, 1H), 8.22 (m, 1H), 8.33 (d, 1H), 8.40 (dd, 1H), 9.41 (d, 1H), 11.47 (t, 1H).

##### *tert*-Butyl (*S*)-(1-Oxo-3-(2-oxo-1,2-dihydropyridin-3-yl)propan-2-yl)carbamate (S9)

In a 100 mL round-bottom flask, **S2a** (2 mmol, 1 equiv, 650 mg) was dissolved in 15 mL of anhydrous THF and cooled to −10 °C. Under nitrogen, 2 M LiAlH_4_ in THF (2.4 mmol, 1.2 equiv, 1.1 mL) was added dropwise into the reaction mixture, followed by stirring for 30–40 min. After completion, the reaction was slowly quenched with a few drops of a 0.1 N HCl solution at 0 °C. THF was evaporated, and excess water was added to the mixture. The reaction mixture was extracted with EtOAc (3 × 50 mL). The organic layer was washed with brine solution (3 × 50 mL). The compound was purified using silica gel column chromatography (2–3% MeOH in DCM, (v/v)) to give **S9** (yield: 260 mg, 50%). ^1^H NMR (400 MHz, DMSO-D_6_) δ 1.35 (s, 9H, Boc), 2.46 (d, *J* = 12 Hz, 1H, 2-Pyrd-CH_2_), 2.93 (dd, *J* = 4, 8 Hz, 1H, 2-Pyrd-CH_2_), 4.13 (dq, *J* = 4, 8 Hz, 1H, CH_α_), 6.13 (t, *J* = 4 Hz, 1H, 2-Pyrd-C–H), 7.25 (s, 1H, Boc-N–H), 7.27 (d, *J* = 8 Hz, 2H, 2-Pyrd-CH_2_), 9.48 (s, 1H, CHO), 11.57 (s, 1H, 2-Pyrd N–H). ^13^C NMR (100 MHz, DMSO-D_6_) δ (28.56, 30.08, 58.74, 78.85, 105.36, 128.30, 133.99, 140.03, 156.01, 163.02, 201.81). 15 mg powder of **S9**, when dissolved in CDCl_3_ tautomerized into **S10**. ^1^H NMR (400 MHz, CDCl_3_) δ 1.42 (s, 9H, Boc), 3.05 (d, *J* = 4, 8 Hz, 2H, pyridyl-CH_2_), 4.33 (q, *J* = 4, 8 Hz, 1H, C–H_α_), 6.16 (s, 1H, Boc-N–H), 6.29 (t, *J* = 4 Hz, 1H, pyridine C–H), 7.39 (td, *J* = 4,8 Hz, 2H, Pyridine C–H), 9.62 (s, 1H, anomeric C–H), 12.91 (s, broad, lactol O–H). ^13^C (28.32, 30.57, 60.00, 79.90, 107.45, 127.38, 133.72, 141.94, 155.78, 165.32). **S9** (LC-MS: *t*_R_ = 2.11 min, C_13_H_18_N_2_O_4_; *m*/*z* calcd 266.1, found 167.2 (M-Boc).

#### Cathepsins

Recombinant cathepsins were obtained from the following vendors: recombinant human cathepsin L (Millipore Sigma or R&D Systems Inc.), and recombinant cathepsin B (Millipore Sigma or R&D Systems), and were used without further purification. Recombinant human cathepsin L was also prepared in-house as described below. Proteins were dissolved or aliquoted into solutions of 50 mM sodium acetate (pH 5.5), 1 mM Na_2_EDTA, 1 mM CHAPS, 10% (v/v) DMSO, and 5 mM DTT to final protein concentrations of 1–10 mM aliquots and stored at −80 °C until needed. These protein samples were then diluted into the same buffer to concentrations of ∼100 nM, and these dilutions were stored at 4 °C and used daily until depletion.

### Expression and Purification of SARS-CoV-2 3CL protease (3CL-PR)

The expression and purification of the 3CL-PR was as previously described.^19,36^ Briefly, an expression construct of SARS-CoV-2 3CL-PR encoding a reading frame which will express 3CL-PR in its nascent form as N-term-GST-tag/SAVLQ*SGF-3CL-PR-SAVLQ*SGF/SGVTFQ*GP/His_6_-tag-C-term. The N-terminal region contains a GST-tag followed by the native 3CL-PR cleavage sequence (SAVLQ*SGF), while the C-terminal region contains a modified HRV-3C protease cleavage site sequence (SGVTFQ*GP), followed by a His_6_-tag. During expression, autoproteolysis by 3CL-PR removed the GST sequence, yielding the native N-terminal sequence Ser-Gly-Phe. This material then was bound to a nickel-NTA column, and the protease was eluted with buffer containing 500 mM imidazole, and eluted fractions were pooled and dialyzed. The C-terminal His_6_ tag was proteolyzed by incubation with 3.5 units of HRV 3C Protease (Thermo Fisher Scientific) per milligram of 3CL-PR at 4 °C overnight. The protein mixture was then chromatographed on a 5 mL GSTrap HP column, and then a 5 mL HisTrap HP column (GE Healthcare), to remove, respectively, the GST-fused HRV 3C protease and undigested His_6_-tagged protein. After chromatography on an anion exchange column and a gel-filtration column, the tag-free 3CL-PR was pooled and concentrated (10 kDa molecular weight cutoff filter, GE Healthcare). The protein was deemed to be ≥95% pure by SDS-PAGE, and was stored at −80 °C in 12 mM Tris–HCl, 120 mM NaCl, 0.1 mM EDTA, 2 mM DTT, (pH 7.5) with 50% glycerol (v/v). Analytical gel filtration indicated that native 3CL-PR was the expected homodimer (68 kDa).

Human cathepsin L (Uniprot P07711) was expressed in *E. coli* and purified by inclusion body refolding and size exclusion chromatography. A plasmid containing a construct that encompasses the canonical 333-amino acid sequence of procathepsin L was cloned into the pET-28a(+) vector (Genscript) and transformed into the C43(DE3) *E. coli* strain. Isolated colonies were grown overnight at 37 °C with shaking at 180 rpm in Difco Terrific Broth containing 50 μg/mL kanamycin. These cultures were then scaled to 500 mL under the same conditions and grown to an OD = 0.4–0.6, followed by induction with IPTG to a final concentration of 1 mM. Induced cultures were grown for 24 h at 37 °C, 180 rpm. Cells were pelleted and stored at −20 °C until purification. Pellets were suspended in lysis buffer (50 mM Tris HCl (pH 8.0), 2 mM EDTA, 5% (w/v) saccharose, 30 mg/L DNase 1, 30 mg/L RNase 1) and were sonicated at 61% amplitude, 15 × 10 s. The resulting lysate (6 mL buffer/1 g pellet) was centrifuged at 16,000*g* for 30 min. The pellet containing inclusion bodies was resuspended in buffer (50 mM Tris HCl (pH 8), 2 mM EDTA, 0.1% TritonX-100) and stirred for 30 min before centrifugation. Resuspension and washing of the inclusion bodies were repeated twice with 50 mM Tris HCL (pH 8) and 1 M urea before solubilization of inclusion bodies in 50 mM Tris HCL (pH 8), 5 mM EDTA, 6 M guanidinium HCl, 150 mM NaCl, and 10 mM DTT. Inclusion bodies that were solubilized were then diluted 1:50 at a rate of 0.5 mL/min into 50 mM Tris HCl (pH 8.5), 0.5 M l-arginine, 0.01% Brij-35, 10 mM NaCl, 1 mg/mL catalase (Thermo Scientific, ≥3000 units/mg protein), and 10 mM each of reduced and oxidized glutathione, followed by stirring for 48 h at 4 °C. The sample was then dialyzed against 25 mM sodium phosphate (pH 7.0) and 0.5 mM NaCl, followed by concentration to 1 mg/mL protein by ultrafiltration using an Amicon Stirred Cell with a 10 kDa cutoff filter. The filtrate was buffer-exchanged against Activation Buffer (20 mM sodium acetate (pH 4.5), 300 mM NaCl, 5 mM DTT, 2.5 mM EDTA, 0.01% NaN_3_, and 0.01% Brij-35) via size exclusion chromatography (HiLoad 26/600 Superdex 200 pg). Fractions containing hCatL were combined and protein was captured, isolated, and collected from Ni-NTA affinity chromatography (Ni-NTA agarose (Qiagen)). The resulting protein was concentrated to 5 mg/mL before activation by autoproteolysis at 37 °C for 30 min. Activated protein was aliquoted with added 40% (v/v) glycerol and frozen at −80 °C. Protein purity and identity were confirmed by SDS-PAGE (Figure S13) and the molecular weight of the autoprocessed enzyme was determined to be 24.2 kDa (Figure S14).

### Kinetic Assays and Characterization of Inhibition for Human Cathepsin B and L

For both cathepsins, reaction mixtures were prepared identically. All stocks of inhibitors and fluorescent substrates, Cbz-Phe-Arg-7-amino-4-methyl-coumarin (Z-FR-AMC) or Cbz-Phe-Leu-7-amino-4-methyl-coumarin (Z-LR-AMC), were dissolved and diluted in 100% DMSO and added to reaction mixtures to the desired final concentrations. The reaction buffer consisted of sodium acetate (pH 5.5), 1 mM CHAPS, 1 mM Na_2_EDTA, and 5 mM DTT at 25 °C. For each run, prediluted aliquots of the inhibitor and fluorescent substrate were added to the 0.25 mL volume reaction buffer such that a final concentration of 10% (v/v) DMSO was maintained. Reactions were initiated by the addition of the proteases to final concentrations of 1–10 nM of either cathepsin L or cathepsin B. Michaelis constants for Cbz-Leu-Arg-AMC (Z-LR-AMC) were determined for cathepsin L (*K*_m_ = 4.4 μM) and cathepsin B (*K*_m_ = 25 μM), and fixed concentrations of Z-LR-AMC of 1× or 2 × *K*_m_ were used to evaluate inhibitors. The formation of the fluorescent product AMC was monitored over 30–60 min time courses for reaction mixtures in 96-well clear-bottomed, black microplates (Greiner). Rates of peptidolysis of the dipeptide-AMC substrate(s) were measured on either a SpectraMax M2 (Molecular Devices) or a Synergy Mx (Biotek, Winooski, VT) microplate reader which measured the formation of fluorescence using an excitation wavelength of λ_ex_ = 360 nm, with detection of emission at λ_em_ = 460 nm at 6 s intervals. Control samples excluded substrate. The measured relative fluorescence units (RFUs) of generated AMC were converted to reaction rates of μM/s by use of a standard curve of known AMC concentrations obtained for both plate readers.

### Kinetic Analysis of SARS-CoV-2 3CL-PR and Characterization of Its Inhibitors

In reaction mixtures containing 20 mM Tris–HCl (pH 7.5), 150 mM NaCl, 0.1 mM EDTA, 2 mM DTT, 10% (v/v) DMSO (a final concentration of 10% (v/v) DMSO in each sample arising from the addition of substrates and inhibitors added from 100% (v/v) DMSO solutions), and variable concentrations of the FRET-based substrate Abz-SAVLQ*SGFRK(DNP)-NH_2_, the reaction was initiated by the addition of 3CL-PR to final concentrations of 20–50 nM in 96-well plates (Greiner, flat-bottom half volume, clear-bottom black plates). Rates of peptidolysis of the Abz-SAVLQ*SGFRK(DNP)-NH_2_ substrate were measured on either a SpectraMax M5 (Molecular Devices) or a Synergy HTX (Biotek, Winooski, VT) microplate reader with λ_ex_ = 320, λ_em_ = 420 nm in 8 s intervals, and time courses of inhibition were obtained for either 30 or 60 min intervals. Control samples excluded substrate. The measured relative fluorescence units (RFUs) of generated Abz-SAVLQ-COOH were converted to reaction rates of μM/s by use of a standard curve of known concentrations of fully hydrolyzed substrates obtained for both plate readers.

### Cell Cultures and Evaluation of Efficacy in SARS-CoV-2 Infection

Vero E6 cells [CRL:1586, ATCC], derived from African green monkey cells, were grown in Eagle’s minimal essential medium (EMEM) supplemented with standard doses of penicillin and streptomycin, and 10% fetal bovine serum (FBS), which we designate as M-10 medium. SARS-CoV-2 (USA_WA1/2020 isolate), the third passage in Vero E6 cells from the original CDC (Atlanta) material with a confirmed sequence, was used throughout the study. A modified Vero E6-based standard microneutralization assay was used to rapidly evaluate the drug efficacy against SARS-CoV-2 infection. Briefly, confluent Vero E6 cells grown in 96-well microtiter plates were pretreated with 78 nM to 20 μM of the protease inhibitors (2-fold serially diluted) for 2 h, before infection with ∼100 or ∼500 infectious SARS-CoV-2 particles, respectively, in 100 μL EMEM supplemented with 2% FBS (2-MEM). Samples contained 2 μM of the MDR inhibitor CP-100356.^8^ Cells pretreated with 2-fold serially diluted DMSO with or without virus were included as positive and negative controls, respectively. After cultivation at 37 °C for 3 days, individual wells were observed by microscopy for the status of virus-induced formation of the cytopathic effect (CPE). The efficacy of individual drugs was calculated and expressed as the lowest concentration capable of completely preventing virus-induced CPE in 100% of the wells. The values of EC_50_ (the concentration of inhibitor that results in 50% growth of virus) were determined in two ways based on the presentation of the two replicates. If duplicate samples of a single compound dilution both displayed 100% CPE for both replicates and in which no CPE was observed at the next highest concentration of the inhibitor in duplicates, the assigned EC_50_ was the average of these two concentrations. In the event that duplicate concentrations result in one sample displaying CPE while the other does not, the value of EC_50_ was assigned to this concentration. All experiments using infectious viruses were conducted at the University of Texas Medical Branch under BSL-3 conditions.

### Fitting of Kinetic Data

Time-course data for compounds that exhibited time-dependent inhibition were fitted to eq 1, for which *P* is the concentration of the fluorescent product, *v*_i_ and *v*_s_ are respectively the initial (*t* < 300 s) and steady-state (*t* > 1600 s) velocities, *k*_obs_ is the rate constant of conversion of *v*_i_ to *v*_s_, *t* is time in seconds, and *C* is a background constant.
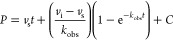
1

In general, resulting values of *k*_obs_ vs [*I*] were replotted and fitted to eq 2, in which *k*_3_ and *k*_4_ represent the respective rates of formation and reversion of the tight-binding EI* complex to the initial **EI** complex, in which *K*_i_ = *k*_2_/*k*_1_ and *K*_i_* = (*k*_2_/*k*_1_)(*k*_4_/(*k*_3_ + *k*_4_)), and for which *A* is the fixed substrate concentration and *K*_a_ is the Michaelis constant.
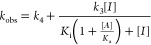
2

Linearity of *k*_obs_ vs [*I*] of inhibitors compounds will be observed when *K*_i_ ≫ *K*_i_*. Under these conditions, the concentration of inhibitor required to observe time-dependent inhibition would be much lower than the value of *K*_i_ for the **EI** complex. In this case, eq 2 reduces to eq 3, which is a linear function with a slope = *k*_3_/*K*_i_ and a *y*-intercept = *k*_4._
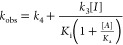
3

Inhibition constants (*K*_i_ and *K*_i_* values) were also obtained by fitting plots of *v*_i_/*v*_0_ and *v*_s_/*v*_0_ vs [inhibitor] to eqs 4 and 5, wherein *v*_i_ and *v*_s_ are velocities at, respectively, early and late stages of the time courses of inhibition, *v*_0_ is *v*_i_ and *v*_s_ when no inhibitor is present, [*I*] is variable concentrations of a competitive inhibitor, and *K*_a_ is the Michaelis constant of the the substrate, *A*.
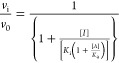
4
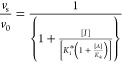
5
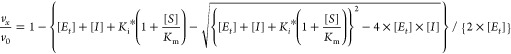
6

Inhibitors for which *K*_i_* and/or *K*_i_ ∼ [*E_t_*] and [*I*] were fit to eq 6, in which *v*_*x*_ is the velocity of the enzymatic reaction at different inhibitor concentrations, *v*_0_ is the velocity of enzymatic reaction in the absence of inhibitors. *E_t_* is the concentration of enzyme in the assay, [*I*] is the inhibitor concentration, *K*_i_ is the inhibition constant when *v*_*x*_ is *v*_0_, *K*_i_* is the inhibition constant when *v*_*x*_ is *v*_s_, [S] is the substrate concentration used in the assay (30 μM final), and *K*_m_ is the Michaelis–Menten constant of Abz-Dnp substrate (36.4 μM). Eq 6 reduces to eqs 4 and 5 when [*E_t_*] ≪ [*I*].

Kinetic plots of inhibitors vs substrates were fitted to both eqs 7 and 8, which conform respectively to competitive and noncompetitive (mixed) inhibition for which *v* is the initial velocity, *V* is the maximum velocity, *A* is the concentration of the variable substrate, *K*_a_ is the Michaelis–Menten constant, and *K*_is_ and *K*_ii_ are the slope and intercept inhibition constants, respectively.
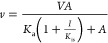
7
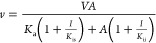
8

### Crystallization of 3CL-PR with BC671

Solutions of 354 μM 3CL-PR and 10 mM BC671 were diluted in 16 mM Tris pH 7.5, 120 mM NaCl, 80 μM EDTA, and 1.7 mM DTT, generating 250 μL of 59 μM protein with 0.2 mM inhibitor. The mixture was incubated on ice for 2 h and concentrated to 236–414 μM, using a 10K cutoff Amicon Ultra-0.5 centrifugal filter. Seed crystals of 3CL-PR incubated with **BC671** (compound **4**) were grown using sitting drops at room temperature with a well solution containing 0.2 M ammonium phosphate, pH 8, 21% (w/v) polyethylene glycol (PEG) 3350. The drops contained a 1:2 ratio of protein to reservoir solution. Thin plates of multilayered crystals formed within 24 h. These crystals were aspirated and crushed using a Seed Bead (Hampton Research) and vortexed with 20 μL of the reservoir solution for 20 s. The crushed seed stock was diluted 100-fold. At room temperature, sitting drops were set up with a reservoir solution containing 0.2 M ammonium phosphate (pH 8), 20% (w/v) PEG 3350, and 0.1 M MES, pH 5.5 with a 1:2 ratio of protein incubated with **BC671** to reservoir solution and immediately seeded with 0.2 μL of the diluted seed stock. The majority of the crystals formed were multilayered plates; however, the crystal used for structure determination was a single isolated thin plate. 3CL-PR crystals grown with the inhibitor **BC671** were harvested and transferred to a cryoprotectant composed of 0.2 M ammonium phosphate pH 8, 20% (w/v) PEG 3350, 20% (v/v) ethylene glycol and flash cooled using liquid nitrogen.

### Crystallization of 3CL-PR with, BC674, VK13, BC787, and VK20 (Compounds 5–8)

Frozen 50 μL aliquots of protein in 20 mM Tris, 100 mM NaCl, and 2 mM DTT at pH 7.5 were thawed and diluted to a final concentration of 177 μM protein by the addition of 20 mM Tris, pH 7.5. Seed crystals were grown via the hanging drop method, at room temperature with a 1 mL reservoir solution containing 0.1 M potassium sodium tartrate and 10% PEG 3350. The drops contained a 1:1 ratio of protein (177 μM) to the reservoir solution. Small needle clusters formed after 3 days. 3–4 of these crystals were aspirated and crushed using a Seed Bead and vortexed with 40 μL of the reservoir solution for 3 min. The crushed seed stock was diluted 20-fold using a reservoir solution and used immediately to microseed room temperature 2.4 μL hanging drops made using a 1:1 ratio of protein solution (177 μM 3CL-PR prepared as stated above) and reservoir solution (100 mM potassium sodium tartrate and 10% PEG 3350). For **VK13**, granules of the compound were added to the drop at the time of microseeding. Granules of **VK20** were added to drops 24 h after seeding. For **BC674** and **BC787**, 27.6 μL of enzyme solution was incubated with residual compound in a 1.5 mL Eppendorf tube (<1 mg of compound total) for 30 min at room temperature to dissolve the compound; 2.4 μL hanging drops were made using a 1:1 ratio of protein solution and immediately microseeded. Long, spindle-like crystals formed after a few days, but the needles continued to grow perpendicular to the long axis for one month before they were harvested. Crystals of sufficient size were immersed in a cryoprotectant solution prior to flash cooling. Crystals were washed in reservoir solution and then cryo-protected by washing in reservoir solution augmented with increasing concentrations of glycerol (10% and 20%). Crystals were immediately flash-cooled by submerging in liquid nitrogen.

### Data Collection, Data Processing, Phasing, and Refinement

X-ray diffraction data were collected remotely at the Stanford Synchrotron Radiation Lightsource beamline 12-2 using the software package Blu-Ice^37,38^ with a wavelength of 0.979 Å at 100 K and a Dectris Pilatus 6 M pixel detector. The oscillation wedge (total ° of data collected) and oscillation angle (° per image), exposure time per image, attenuation, and crystal-to-detector distance are reported in Table 1. The data were indexed and scaled with XDS. The space group, unit cell dimensions, resolution, and other important data collection parameters are reported in Table 1.

**Table 1:** Data Collection and Refinement for 3CLpro Structure with Inhibitors

**Data Collection**					
PDB code	9CEC	9CEK	9CED	9CFB	9CF9
inhibitor	**BC671**	**VK20**	**VK13**	**BC674**	**BC787**
wedge (deg), oscillation angle (deg)	180, 0.15	360, 0.15	360, 0.15	136, 0.1	340, 0.1
exposure (s), attenuation (%)	0.2, 50	0.2, 79	0.2, 16	0.1, 96	0.1, 94
crystal-to-detector distance (mm)	450	175.5	256.8	167.6	267.3
wavelength (Å)	0.979	0.979	0.979	0.979	0.979
space group	*C*2	*P*21	*P*21212	*P*21	*P*21
unit cell dimensions	*a* = 113.74, *b* = 53.52, *c* = 45.94, β = 102.22	*a* = 46.92, *b* = 64.06, *c* = 103.12, β = 90.85	*a* = 45.84, *b* = 64.24, *c* = 105.85	*a* = 46.81, *b* = 63.82, *c* = 102.20, β = 90.97	*a* = 44.79, *b* = 54.05, *c* = 114.87, β = 100.49
resolution range (Å)	34.48–2.36 (2.45–2.36)	37.85–1.38 (1.40–1.38)	37.31–1.82 (1.86–1.82)	37.74–1.45 (1.47–1.45)	39.05–2.00 (2.05–2.00)
*R*_sym_	0.074 (0.705)	0.058 (0.875)	0.053 (0.926)	0.048 (0.326)	0.045 (0.550)
*R*_pim_	0.049 (0.450)	0.035 (0.534)	0.032 (0.564)	0.037 (0.266)	0.032 (0.400)
CC1/2	0.941 (0.692)	0.999 (0.717)	0.999 (0.668)	0.997 (0.809)	0.998 (0.725)
total reflections	37,232 (3424)	872,066 (5762)	194,489 (11,302)	278,639 (12,873)	120,458 (8693)
unique reflections	11,119 (1055)	120,401 (5762)	28,780 (1664)	104,319 (5031)	36,272 (2637)
redundancy	3.3 (3.2)	7.2 (7.1)	6.8 (6.8)	2.7 (2.6)	3.3 (3.3)
completeness (%)	98.8 (91.0)	96.1 (93.9)	99.8 (99.0)	97.8 (95.9)	98.5 (96.0)
mean I/sigma(I)	8.5 (2.1)	14.9 (2.0)	15.4 (2.0)	10.2 (2.5)	10.9 (2.0)
**Phasing**					
LLG	1904	8263	360	7500	8540
TFZ	23.7	84.9	6.4	77.7	12
asymmetric unit	1 monomer	2 monomers	1 monomer	2 monomers	2 monomers
**Refinement**					
resolution range (Å)	34.48–2.36 (2.47–2.36)	37.85–1.38 (1.41–1.38)	37.31–1.82 (1.86–1.82)	37.74–1.45 (1.47–1.45)	39.05–2.00 (2.05–2.00)
*R*_cryst_	0.1941 (0.2798)	0.1343 (0.2205)	0.2234 (0.3521)	0.1725(0.2453)	0.2122(0.3120)
*R*_free_	0.2581 (0.3765)	0.1778 (0.3065)	0.2767 (0.3990)	0.2031(0.2753)	0.2661(0.3644)
reflections used in refinement	11097 (1153)	120368 (8215)	28745 (1867)	104282(7167)	36218(2376)
reflections used for *R*-free	1111 (128)	2000 (139)	2000 (139)	1996(143)	1995(133)
number of non-hydrogen atoms	2368	5456	2465	5236	4765
macromolecules	2323	4871	2344	4702	4658
ligands	35	73	33	69	64
solvent	10	512	88	465	43
protein residues	300	610	299	610	605
RMS (bonds)	0.013	0.008	0.013	0.009	0.014
RMS (angles)	1.272	1.057	1.292	1.094	1.385
average *B*-factor	51.44	15.46	39.97	13.76	36.94
**Ramachandran Plot Analysis**					
Ramachandran favored (%)	96.31	98.84	97.32	99.33	96.81
Ramachandran allowed (%)	3.69	0.99	2.34	0.67	2.85
Ramachandran outliers (%)	0.0	0.17	0.33	0	0.34

The structures were solved by molecular replacement using the Phaser-MR package in the Phenix software suite.^39^ An existing apo structure of a 3CL-PR monomer (PDB: 6M2Q) was used as the search model in Phaser for the structure containing **BC671**, after the removal of water molecules and all other buffer components. All subsequent structures used the **BC671** structure (without water, buffer, or inhibitors) as the molecular replacement model. In each case, Phaser yielded clear solutions: the log-likelihood gain and translation function *z*-score and number of monomers in the asymmetric unit are reported in Table 1 for each structure. In each case, the map generated indicated the density of the ligand in the active site. Model building and refinement were performed with the software Coot and Phenix.Refine.^40−43^ After the amino acid backbone was well refined, the Phenix package eLBOW^44^ was used to generate .pdb and .cif files for each inhibitor, which were placed in the density and refined using LigandFit.^45,46^ Water molecules were added and manually verified in subsequent refinements. Anisotropic B-factors were only used for the high-resolution structure with the **VK20** inhibitor. The Ramachandran statistics calculated by MolProbity^47^ and other refinement statistics are reported in Table 1, including the Protein Data Bank accession codes for the deposition of atomic coordinates and structure factors. The components of the final models (residues, inhibitors, waters, and buffer components) are summarized in Table 2. Structural figures were prepared using Pymol (Schrodinger). In each structure, the electron density map between the inhibitor and Cys_145_ supported the hypothesis that the molecule was covalently bound to the active site.

**Table 2:** Composition of Final Models

3CL-PR-BC671	
protein	residues 1–300
inhibitor	1, covalently attached to Cys_145_
water	10
buffer	1 DMSO on a special position
3CL-PR-VK20	
protein	chain A: residues 1–305, chain B: residues 1–305
inhibitor	3; 1 covalently bound to each Cys_145_, and a third bound at a crystal packing interface (no density for oxazole)
water	513
buffer	1 sodium atom, 1 tartrate
3CL-PR-VK13	
protein	Residues 1–45, 49–306
inhibitor	1 covalently bound to the Cys_145_
water	88
buffer	none
3CL-PR-BC674	
protein	chain A: 1–192, 196–305, chain B: 1–153, 156–305
inhibitor	2; 1 covalently bound to each Cys_145_
water	466
buffer	1 sodium atom
3CL-PR-BC787	
protein	chain A: 1–305, chain B: 1–45, 49–300
inhibitor	2; 1 covalently bound to each Cys_145_, but in different binding poses
water	43
buffer	none

## Results and Discussion

### Chemistry

#### Synthesis of Peptidomimetic Aldehydes (4–8)

The N-*t*-Boc-2-pyridon-3-yl alanine (**S1a**) was synthesized as described,^29^ while N-*t*-Boc-pyridin-3-yl alanine (**S1b**) and N-*t*-Boc-1,3-oxazo-4-yl alanine (**S1c**) were acquired commercially. Each of these N-*t*-Boc amino acids was converted to their Weinreb amides by HATU-catalyzed coupling to N,O-dimethyl hydroxylamine with DIPEA in DMF at 0 °C, to afford **S2a**, **S2b**, and **S2c**, respectively, followed by purification by silica gel chromatography with elution by 40–60% (v/v) EtOAc/hexane, resulted in 70–80% yields (Scheme 1). Trifluoroacetic acid was used to remove the *t*-Boc groups quantitatively to provide **S3a**, **S3b**, and **S3c**, respectively.

Dipeptides **S6a**, **S6b**, and **S6c**, which comprise the P_3_–P_2_ amino acids of the target tripeptide aldehydes, were synthesized by HATU-catalyzed coupling (with DIPEA in DMF at 0 °C) of indole-2-carboxylic acid (Ind, **S4b**) or 4-methoxy-indole-2-carboxylic acid (4-OMe-Ind, **S4a**) with leucine methyl ester (Leu-OMe, **S5a**) or cyclohexyl alanine methyl ester (CHA-OMe, **S5b**), to provide the methyl esters of Ind-CHA (**S6a**), 4-OMe-Ind-Leu (**S6b**), and 4-Ind-Leu (**S6c**), which were then purified by Silica Gel column chromatography with 1:1 (v/v) EtOAc/hexane as the eluant, resulting in 60–80% overall yields. The C-terminal methyl esters were hydrolyzed in a reaction mixture of NaOH in methanol, and the resulting dipeptide acids **S7a**, **S7b**, and **S7c**, respectively, were obtained in >95% yield. Using HATU-catalyzed coupling of the Weinreb amides (**S3a–c**) as described above, the dipeptide acids **S7a**, **S7b**, and **S7c** resulted in the tripeptide amides **S8a**, **S8b**, **S8c**, **S8d**, and **S8e**. Following purification by Silica Gel column chromatography with 2–4% MeOH (v/v) in DCM as the eluant, products **S8a–e** were obtained in 40–60% yield. Finally, using LiAlH_4_-mediated reduction of the Weinreb amide groups of **S8a–e** in THF at −10 °C provided tripeptide aldehydes (**4–8**). Due to severe peak tailing of aldehydes observed during purification by preparative-HPLC, those peaks that corresponded to samples of >95% purity were collected and lyophilized, resulting in <25% yields.

**Figure 2:**
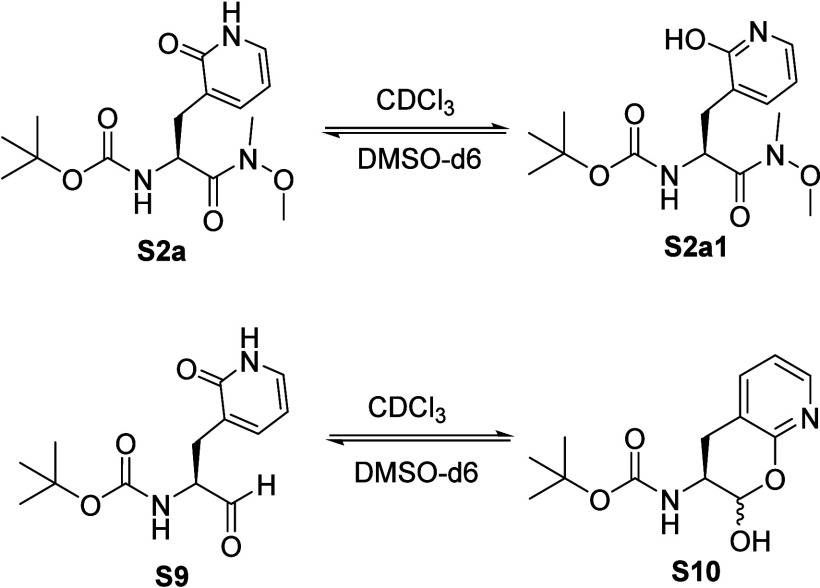
Structures of the lactam (**S2a** and **S9**) and lactim (**S2a1** and **S10**) tautomers of 2-pyridone-containing amino acid analogues.

**Figure 3:**
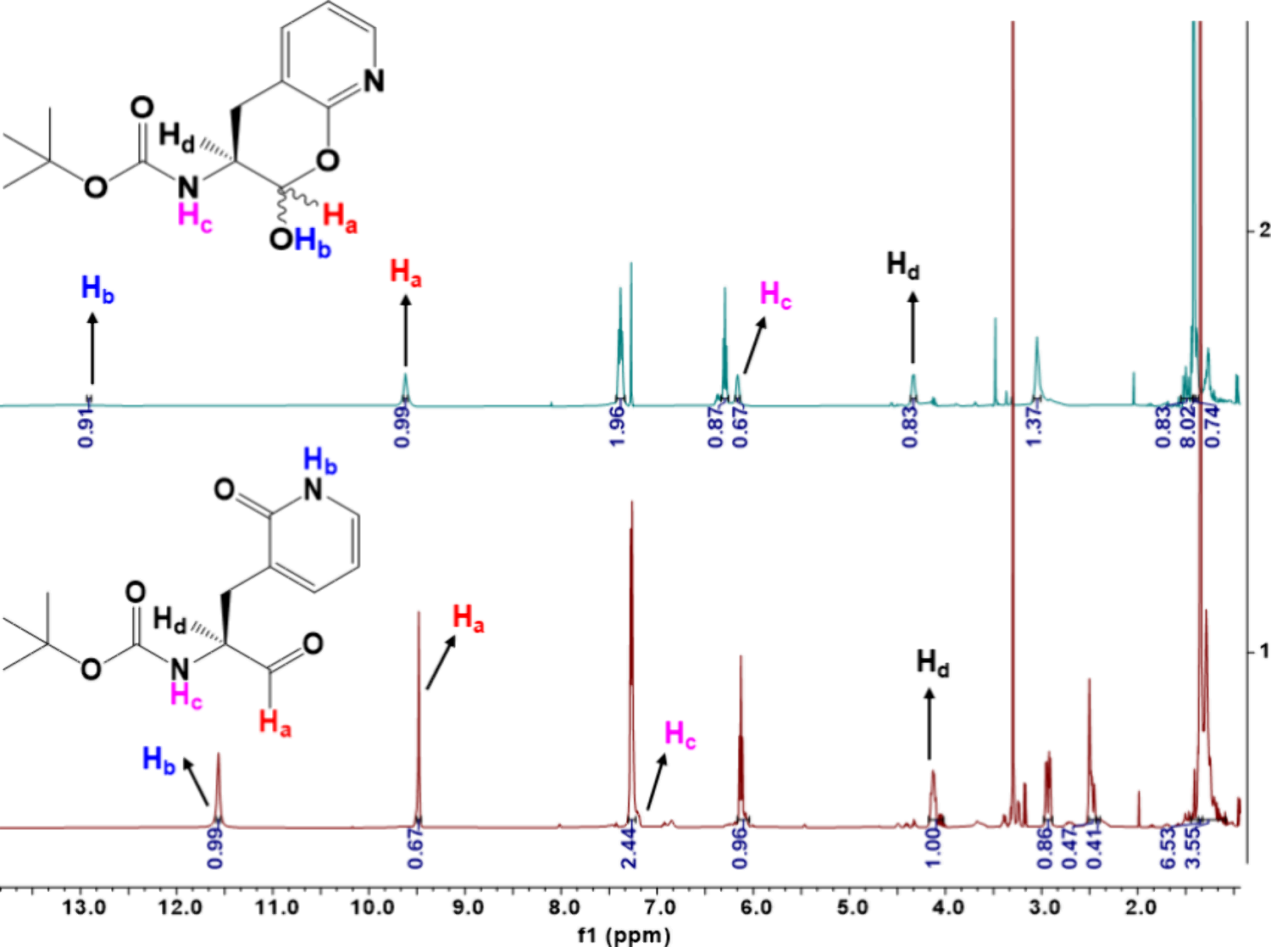
^1^H NMR spectra of compound **S9/S10** obtained in CDCl_3_ (Top) and DMSO-*d*_6_ (bottom). The different chemical shifts of the proton attached to either the δ-lactol anomeric oxygen (H_a_) or the nitrogen (H_b_) of the heterocyclic group demonstrated that the lactim (**S10**) and lactam (**S9**) tautomers are the respective predominate species in the nonpolar and polar solvent. H_c_ and H_d_ in spectra are Boc-NH and alpha protons, respectively. After cyclization, aldehydic proton H_a_ is converted to the lactol anomeric proton in CDCl_3_.

**Figure 4:**
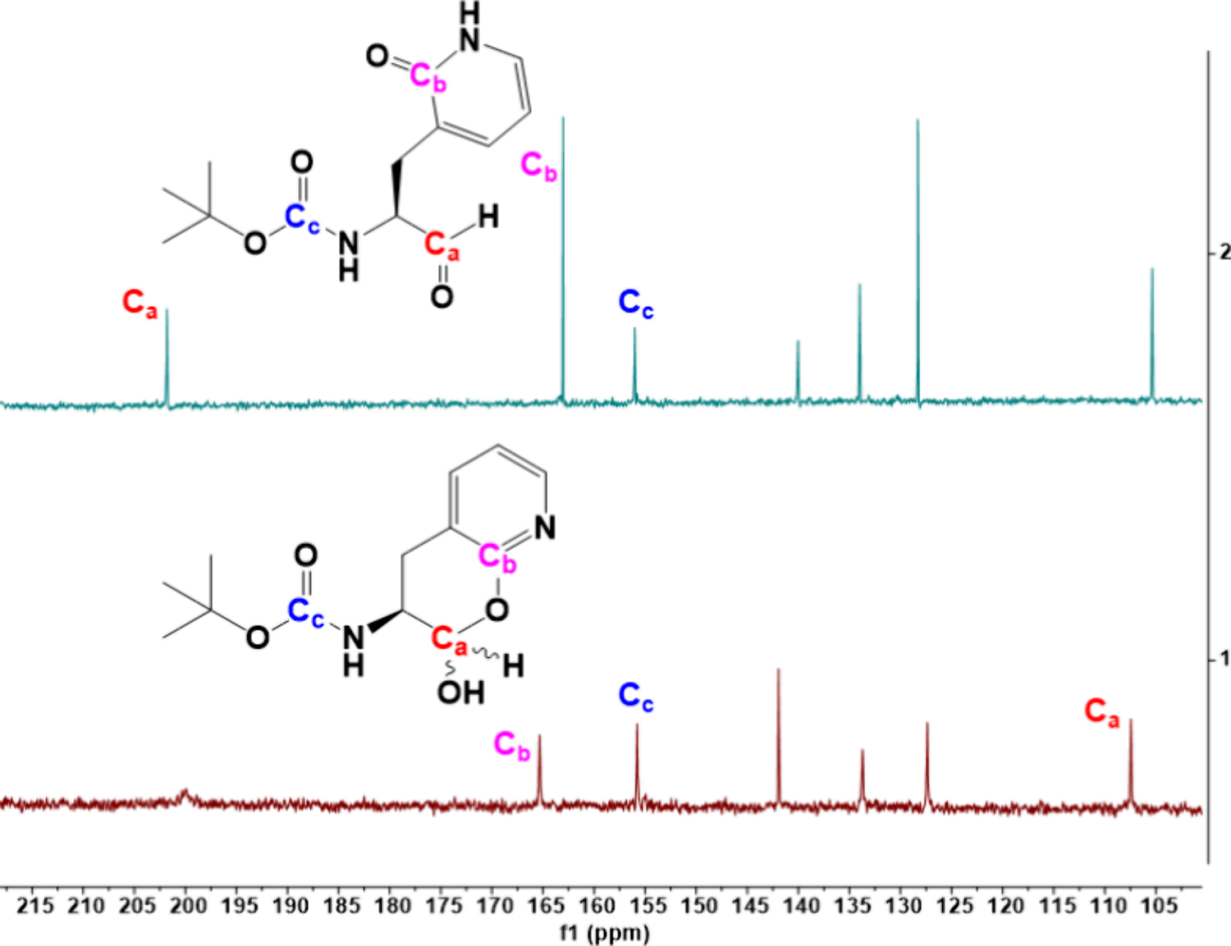
^13^C NMR spectra of N-*t*-Boc-2-pyridon-3-yl-alanal (**S9/S10**) obtained in DMSO-*d*_6_ (top) and CDCl_3_ (bottom) with three carbon atoms indicated (C_a_–C_c_). The chemical shifts of the aldehydic carbon (C_a_) is missing in the bottom spectra which is converted to the anomeric lactol carbon in CDCl_3_. C_c_ is the *t*-Boc carbonyl carbon and C_b_ is the lactam carbon in DMSO-*d*_6_ and the lactim carbon in CDCl_3_ which is slightly more downshielded.

We previously reported that compound **1**, a self-masked aldehyde inhibitor (SMAI) of cruzain^29^ and human cathepsin L^30^ but not 3CL-PR, exists in physiological solutions as a δ-lactol due to intramolecular cyclization between the phenolate and aldehyde (Figure 2). The incorporation of a nitrogen atom into the phenolic group of compound **1** (compound **2**, X = N) provided a potent 3CL-PR inhibitor (*K*_i_ = 250 nM). These data raise the question of whether the 2-pyridone group in compound **2** also forms a δ-lactol. Formation of the δ-lactol of 2-pyridone would involve the 2-hydroxy pyridine tautomer, for which it is known that the difference in the free energy (Δ*G*) of the two tautomeric forms is sufficiently small that the polarity of the solvent can determine which tautomer is preferred.^48,49^ Nonpolar solvents favor the 2-hydroxypyridine tautomer whereas polar solvents favor the 2-pyridone tautomer.^48,49^ In the tautomeric species of N-*t*-Boc-2-pyridon-3-yl-alanyl-(*N*-methoxy)methylamide (**S2a/S2a1**) (Figures S3 and S4) and N-*t*-Boc-2-pyridon-3-yl-alanal (**S9/S10**) (Figures 3 and 4) in a polar (DMSO-*d*_6_) and a nonpolar (CDCl_3_) solvent were evaluated by proton and ^13^C NMR. In the proton NMR spectra of N-*t*-Boc-2-pyridon-3-yl-alanyl-(*N*-methoxy) methylamide, the chemical shift of proton H_a_ changed from δ^1Ha^ ∼ 11.5 ppm in DMSO-*d*_6_ to δ^1H^ ∼ 13 ppm in CDCl_3_ (Figure S3), indicating that the lactim (**S2a1**) and lactam (**S2a**) tautomers are the predominate forms in, respectively, the polar and nonpolar solvent.

The proton at ∼13 ppm in CDCl_3_ is clearly more depleted by exchange with deuterium than the amide proton at 11.5 ppm in DMSO-*d*_6_, as expected, considering the higher acidity of the phenolic proton. For N-*t*-Boc-2-pyridon-3-yl alanal (**S9/S10**), we likewise investigated the nature of its tautomeric forms in DMSO-*d*_6_ and CDCl_3_ using both proton and ^13^C NMR. An aldehydic proton (H_d_, δ^1H^ ∼ 9.48 ppm and a lactam N–H proton (H_c_, δ^1H^ ∼ 11.57 ppm); (Figure 3) was observed in the proton NMR spectra of N-*t*-Boc-2-pyridon-3-yl alanal in DMSO-*d*_6_, and also in the ^13^C NMR, a carbon with the chemical shift of an aldehyde was observed (C_a_, δ^13C^ ∼ 201.8 ppm; Figure 4), indicating that the aldehyde form **S9** predominates in this polar solvent. Furthermore, carbon-2 of the 2-pyridone/hydroxy-pyridine ring displayed a chemical shift that was consistent with a lactam carbonyl (C_b_, δ^13C^ ∼ 162.9 ppm), from which a δ-lactol does not form. In contrast, in the nonpolar solvent CDCl_3_, both proton and ^13^C NMR spectra were consistent with the formation of a δ-lactol from the aldehyde and the lactim tautomer of the 2-pyridone (Figure 4). The chemical shift of carbon-2 of 2-hydroxypyridine (C_b_) (δ^13C^ ∼ 165.3 ppm) is consistent with the lactol/lactim structure of **S10**. This is corroborated by the proton NMR spectra of this compound (Figure 3). Proton NMR of compound **S9/S10** in CDCl_3_ indicated the formation of δ-lactol, characterized by chemical shifts of the anomeric C–H proton (H_a_, δ^1H^ ∼ 9.62 ppm) and lactol O–H proton (H_b_, δ^1H^ ∼ 12.91 ppm) (Figure 3).

Proton NMR data of compounds (**4–8**) in DMSO-*d*_6_ indicated that they all exist exclusively as aldehydes. Accordingly, the inhibition data below arise from the aldehyde forms of compounds **3–6**, while aldehydes are the only forms available to **7** and **8**.

### Kinetic and Anti-CoV-2 Characterization of Dual-Targets Inhibitors

The 2-pyridon-3-alanyl (2PyrdAla) P_1_ side chain within a peptidomimetic aldehyde was our first glutamine isostere designed to be accommodated at the S_1_ sites of both hCatL and 3CL-PR.^29^ We installed the 2Pyrd-Ala-aldehyde into the P_3_–P_2_ peptide scaffold of **K11777** in order to compare activity with that of **K11777** and SMAI **1** which all have nearly identical peptidomimetic scaffolds. As mentioned above, Compound **2** differs from **1** by the single nitrogen atom replacement, but this small change is sufficient to render **2** to be not only a potent inhibitor of hCatL (*K*_i_ = 1 nM), but also a submicromolar inhibitor of 3CL-PR (*K*_i_ = 240 nM), whereas compound **1** did not inhibit 3CL-PR (Figure 1). This encouraged our exploration of the 2Pyrd-Ala-CHO moiety in other peptidomimetic scaffolds that were similar to or identical to those of Pfizer drugs **Nirmatrelvir** and **PF-835231**. In compound **3** (**LL478**) the Cbz-Leu-cyclohexyl-alanine (CHA) scaffold is similar to that of **Nirmatrelvir**. The minor differences here include a Cbz-Leu group substituting for the trifluoro-acetamido-*tert-*Leu-Ala N-terminus of **Nirmatrelvir**, and a cyclohexyl-alanine substituting for the modified proline/leucine group of **Nirmatrelvir** (Table 3). Compound **3**(29) demonstrated reversible time-dependent inhibition of both hCatL (*K*_i_ = 8 nM) and 3CL-PR (*K*_i_ = 9 nM), and was only 3-fold less potent vs 3CL-PR than **Nirmatrelvir**, the latter of which had no activity vs hCatL. Compound **3** was a competitive inhibitor of both hCatL and 3CL-PR and for the latter enzyme, its values of *K*_i_ decreased from 16 nM to 9 nM upon longer incubation time (Figure S1). Compound **3** was only 2-fold less potent in inhibiting SARS-CoV-2 infection of Vero E6 cells in a head-to-head study (EC_50_ = 0.47 vs EC_50_ = 0.23 μM for **Nirmatrelvir** and **PF-835231** (Table 3 and Table S1)).

**Table 3:**
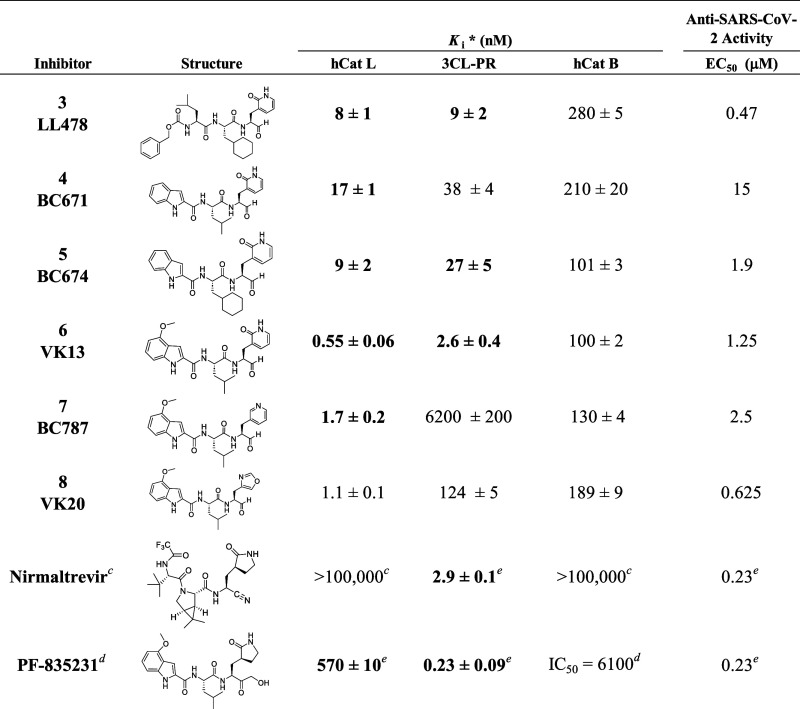
Dual-Target Inhibitors of Human Cathepsins L and SARS-CoV-2 3CL Protease with P_1_-Glutamine Isosteres^a^

a Inhibition studies were performed at 25 °C and at pH = 5.5 for human cathepsins B and L and pH = 7.5 for SARS-CoV-2 3CL protease, respectively. The *K*_i_ values for inhibitors that exhibited time-dependent inhibition were obtained at *t* ∼ 30 min, and are shown in bold. 2 μM CP-100356, a pgp (MDR) inhibitor, was included in the Vero E6 samples unless noted by an asterisk.

b Data for these inhibitors were as reported in Li et al. and Zhu et al.

c Owens et al.

d Boras et al.

e Data were ascertained in the current study.

**Figure 5:**
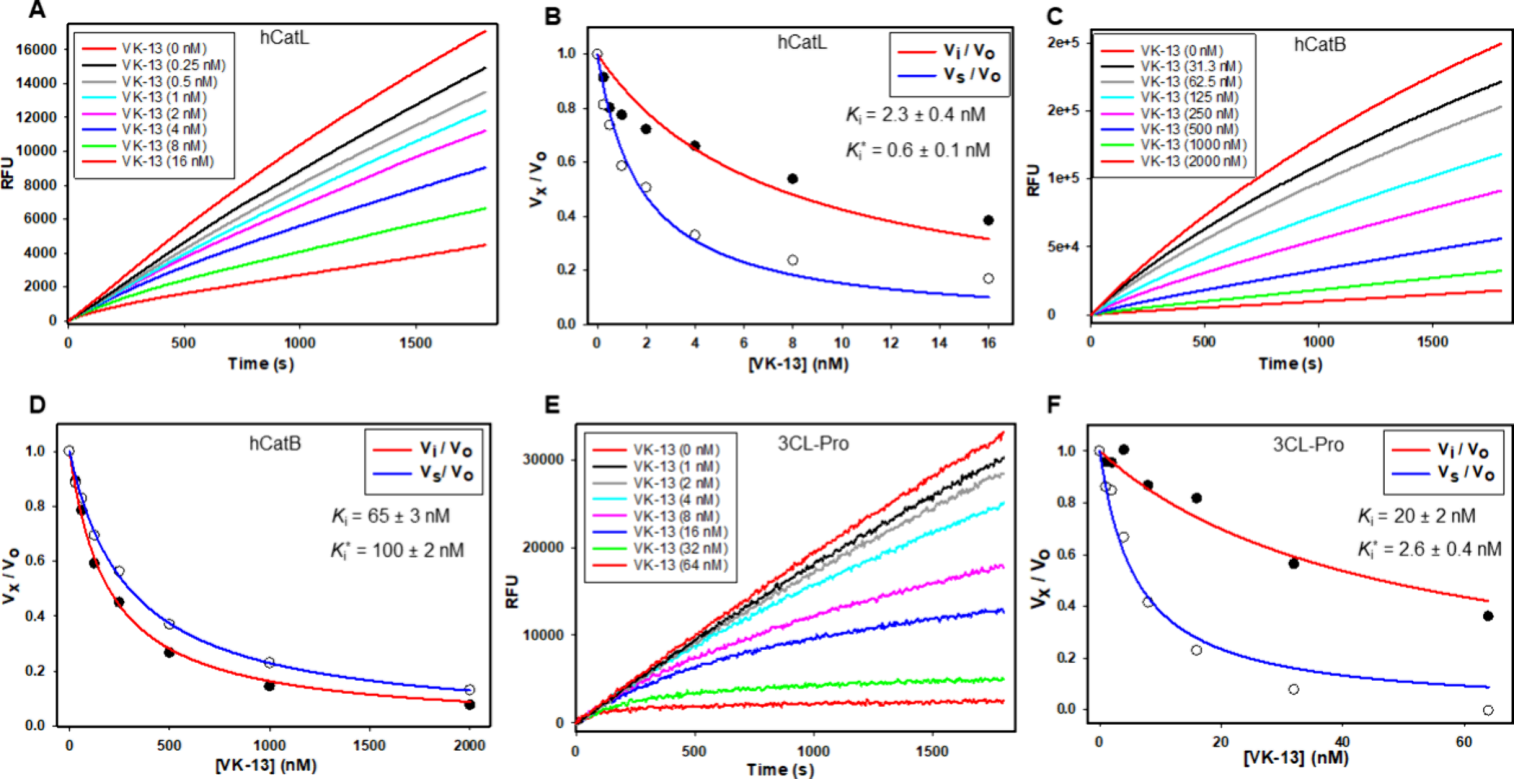
Inhibition of human cathepsin L, human cathepsin B, and 3CL protease by compound **6** (**VK13**). Inhibition by compound **6** was observed in the time courses of peptidolysis reactions of human cathepsin L (A; 0–16 nM), human cathepsin B (C; 0–2000 nM) and 3CL protease (E, 0–64 nM). Data from the time courses were fitted to eq 1. Plotting of the fraction of remaining enzyme activity (*v*_*x*_/*v*_0_) vs inhibitor concentrations at initial rates (*t* = 0–180 s, (*v*_i_/*v*_0_)) and steady-state rates (*t* = 1820–2000 s, (*v*_s_/*v*_0_)) for cathepsin L (B), cathepsin B (D), and 3CL protease (F). The lines drawn through the experimental data points resulted from fitting the data to eqs 4, 5, or 6, from which the inhibition constants were obtained.

The compound **PF-835231** has a 4-methoxy-indol-2-yl-Leu scaffold, contains a 2-oxo-pyrrolidin-3-alanyl (OpA)^35^ group at the P_1_ position group with an α -hydroxymethyl ketone warhead (Figure 1, Table 3), and also entered clinical trials for COVID-19 as a prodrug.^27^**PF-835231** (*K*_i_ = 0.23 nM) is more than 10-fold more potent than **Nirmatrelvir** vs 3CL-PR, and interestingly, in our hands and unreported by Boras et al.,^27^ we found **PF-835231** to be a moderately potent inhibitor of hCatL (*K*_i_ = 570 nM) (Table 1). **Olgotrelvir**, a recent entry in this area of research, is a bisulfite adduct of the aldehyde **AC1115** (Figure 1).^27^ It has an indol-2-yl-Leu scaffold, contains an OpA group at the P_1_ position, is a dual inhibitor of 3CL-PR and hCatL, and is a Phase 3 clinical candidate for the treatment of COVID-19.^26^ Accordingly, we evaluated (4-methoxy)-indol-2-yl-Leu peptidomimetic scaffolds that resemble that of the (4-methoxy)-indol-2-yl-leucine P_3_–P_2_ side chain of **AC1115** or **PF-835231**, which provides a more compact peptide scaffold than **Nirmatrelvir.** Compounds **4** (**BC671**) and **5** (**BC674**) feature an unsubstituted indol-2-yl moiety, like that of **AC1115**. Both inhibitors were considerably less active than **PF-835231** (*K*_i_ = 190 and 60 nM, respectively, vs 0.23 nM), and were poorly active in SARS-CoV-2-infected Vero E6 cells (Table 3, Figures S2, and S3). The conservative substitution of the CHA group in **5** (a competitive inhibitor of hCatL) for the leucine of **4** resulted in 2-fold and 1.5-fold improvement in inhibition of hCatL and 3CL-PR, respectively,

Compound **5** (**BC 674**) inhibited SARS-CoV-2 infection in Vero E6 cells with EC_50_ = 1.9 μM, which is eight-fold less active than **Nirmatrelvir** and **PF-835231** (Figure S3). Compound **6** (**VK13**, Figure 5) was an exceptionally potent inhibitor of both hCatL (***K*_i_** = 0.55 nM) and 3CL-PR (*K*_i_ = 2.6 nM), and its anti-CoV-2 activity (EC_50_ = 1.25 μM) was only five-fold less potent than the Pfizer inhibitors. While unknown, the 4-fold less anti-CoV-2 activity of compound **6** in comparison to its homologue **AC1115** is likely due to poor cell penetrance, although both inhibitors have not been evaluated in cellular assays under the same conditions. The structures of compounds **4** and **6** differ only by the 4-methoxy group on the indole group, but this modest substitution accounts for the 30-fold and 15-fold improvement in inhibition of hCatL and 3CL-PR, respectively. Retaining the 4-methoxy-indol-2-yl-leucine P_3_–P_2_ scaffold of **PF-835321**, we evaluated two additional glutamine isosteres as P_1_–CHO dual-target inhibitors. The highly conservative removal of the oxygen atom of the 2-pyridone side chain of **6** (**VK13**) resulted in the 3-pyridin-3-alanyl glutamine isostere found in compound **7** (**BC787**). Compound **7** (hCatL, *K*_i_ = 1.7 nM; 3CL-PR, *K*_i_ = 6200 nM) was a less effective inhibitor of either protease than **6**, particularly for 3CL-PR, suggesting that the pyridone oxygen is critical to mimicry of the glutamine side chain (Figure S4). This diminution of inhibitory activity vs hCatL (3-fold decrease) is reflected in the 2-fold loss of activity in CoV-2-infected cells (EC_50_ = 2.5 μM), and given the very poor potency vs 3CL-PR, suggests that the inhibition of hCatL is primarily responsible for the anti-CoV-2 activity of **7**. In compound **8** (**VK20**), the 1,3-oxazol-4-yl-alanine-CHO P_1_ group was shown by molecular modeling to be a likely surrogate for glutamine in the active site of 3CL-PR. While **8** was a subnanomolar inhibitor of hCatL (*K*_i_ = 0.87 nM), the oxazole mimicry of a glutamine side chain in the P_1_ site of 3CL-PR inhibitor was apparently not ideal (*K*_i_ = 124 nM) (Figure S5). Nonetheless, **8** exerted potent anti-CoV-2 activity (EC_50_ = 0.62 μM), which again suggested that its anti-CoV-2 activity arises solely from the inhibition of hCatL.

### Selectivity

We assessed our hCatL/3CL-PR dual inhibitors vs human cathepsin B as a measure of protease selectivity. Compounds **3–8** inhibited hCatB over a range of *K*_i_ = 100–480 nM, for which selectivity of the inhibitors for hCatL vs hCatB was 13–220-fold. Less selectivity was found vs 3CL-PR, selectivity was 1.5–36, for which the pyridine and oxazole P_1_ side chains had the least selectivity between 3CL-PR and hCatB, most likely due to poor accommodation of these side chains in the S_1_ site of 3CL-PR.

### X-ray Crystallographic Analysis of Inhibitors Bound to 3CL Protease

Existing crystal structures of compound **3**,^29^**Nirmatrelvir**,^8^ and **PF-835231**(27) bound to 3CL-PR provided a starting point for the assessment of how the 2-pyridone group at the P_1_ position of our tripeptide aldehyde inhibitors interacts with the enzyme in comparison to other glutamine isosteres, such as the OpA groups of the **Nirmatrelvir**, **PF-835231**, and **AC1115**. To explore these comparisons, we obtained crystal structures for inhibitors **4–8** bound to 3CL-PR.

Interestingly, the five new structures all had distinctly different crystallographic systems (space groups and/or unit cell dimensions) despite being grown from a standard crystal seedstock. In three cases, the proposed biological assembly, a homodimer, was found in the asymmetric unit (for compounds **5**, **7**, and **8**), whereas in structures of 3CL-PR-**4** and 3CL-PR-**6**, the asymmetric unit comprised a monomer from which the dimer was constructed by crystallographic symmetry operators (Figure S11).

**Figure 6:**
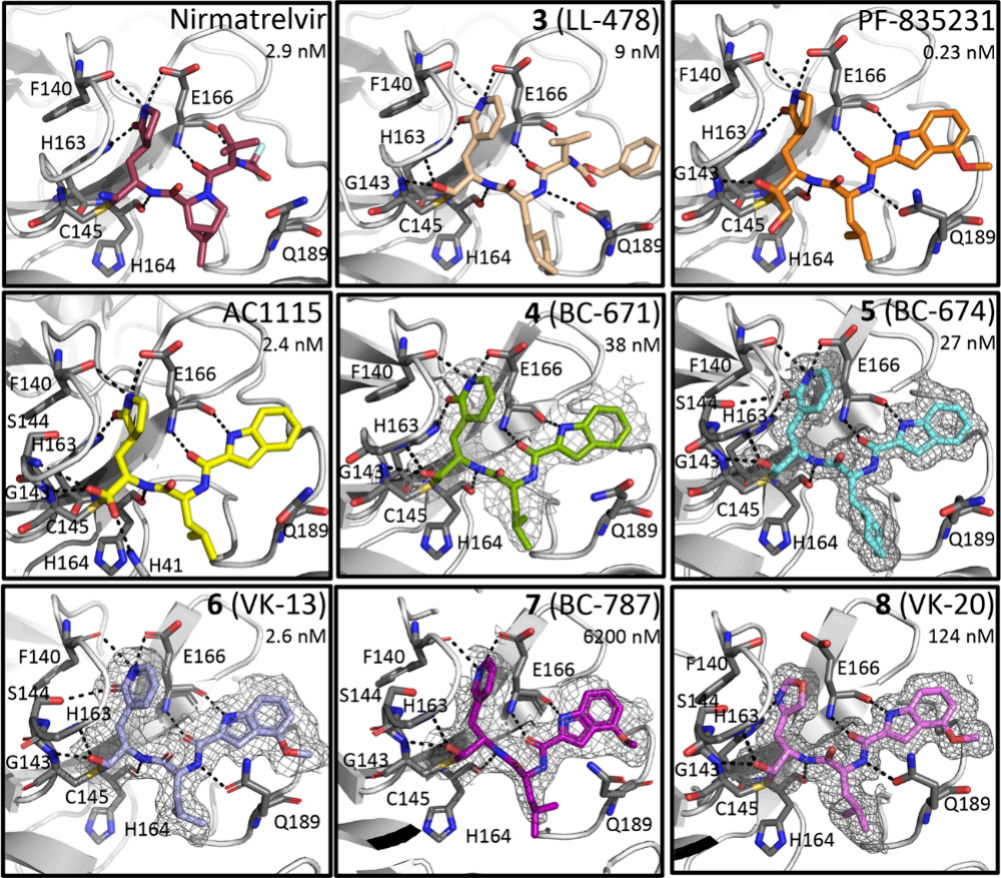
X-ray crystallographic analysis of inhibitors bound to 3CL protease. Potential hydrogen bonds are indicated by dashed lines, and inhibition constants (*K*_i_) are displayed in each panel. The active sites with inhibitors bound are shown for (top row) **Nirmatrelvir** (PDB entry 7RFS)) and **LL478** (**3**; (PDB entry 7M2P)), shown together due to their similar peptidomimetic scaffolds, along with **PF-835231** (PDB entry 6XHM). (Middle row) Structures of inhibitors **AC1115** (PDB 8UAB),^26^ and its analogues **BC671** and **BC674** (this work) to allow comparison of the binding of the substituted and unsubstituted indole groups. (Bottom row) Inhibitors **VK-13 (6**), **BC787**, (**7**) and **VK20** (**8**) (determined in this work) allow comparison of three different heterocyclic groups at the P_1_ within the same peptidomimetic scaffold. The Polder maps contoured at 3σ are shown for the structures determined in this study. Polder maps^50^ are omit maps that enhance visualization by excluding bulk solvent, used here because the inhibitor binds on a solvent exposed site. 2Fo-Fc maps are found in Figure S13.

**Figure 7:**
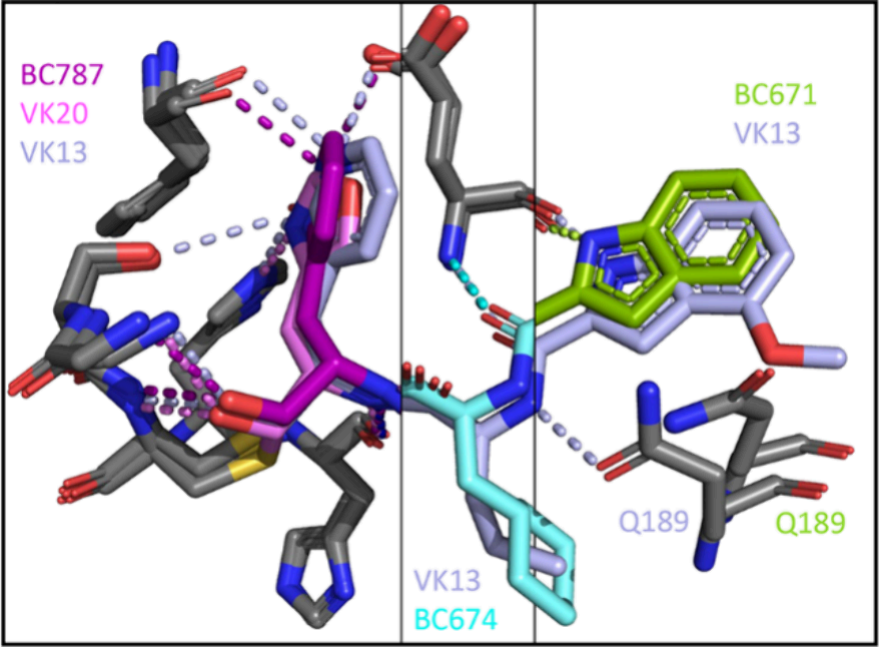
Structural overlays of P_1_ side chains of inhibitors **6–8** (respectively, the 2-pyridon-3-alanyl group of **VK13**, the pyridin-3-alanyl group of **BC787**, and the 1,3-oxazo-4-alanyl group of **VK20**) in the S_1_ site of 3CL-PR, the P_2_ side chains of inhibitors **5** and **6** (respectively, the cyclohexyl-alanyl and leucyl groups of **BC674** and **VK13** in the S_2_ site of 3CL-PR, the pyridin-3-alanyl group of **BC787**, and the 1,3-oxazo-4-alanyl group of **VK20**), and the P_3_ side chains of inhibitors **4** and **6** (respectively, the indoyl group of **BC671** and the 4-methoxy-indoyl group of **VK13**, the pyridin-3-alanyl group of **BC787** in the S_3_ site of 3CL-PR).

**Figure 8:**
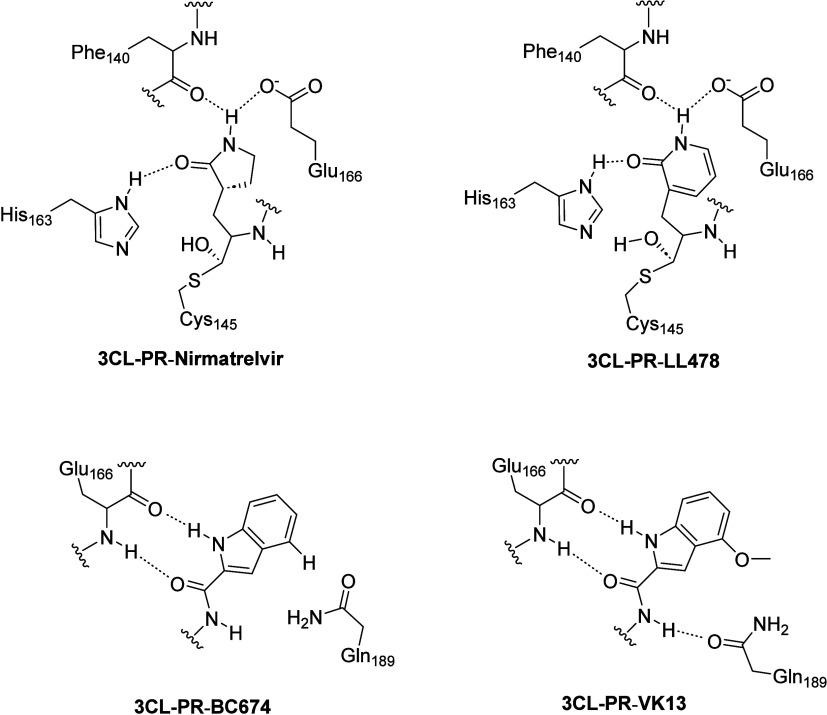
(top) Schematic structures of 3CL-PR-bound **Nirmatrelvir** and **LL-478** (**3**) in which identical hydrogen bonds are formed for the respective OpA and 2-Pyrd P_1_ groups of the two inhibitors. (bottom) Schematic structures of 3CL-PR-bound **BC674** and **VK13** indicating common hydrogen bonds between the enzyme and the indole-carbonyl heteroatoms, and the distinct additional hydrogen bond between the 4-methoxy group of **VK13** and the γ-carbonyl oxygen of Gln_189_.

Regardless of the crystal system, the individual monomers showed a high level of structural similarity with root-mean-square deviations (rmsd) per monomer chain ranging from 0.142 to 0.614 Å over 240–289 Cα carbons, depending on chains being compared, indicating that binding of the different inhibitors did not change the overall structure of the protein. When compared to the previously determined structures that are also part of the discussion in this work, the rmsd values range from 0.142 to 1.48 Å over 240–293 C-alpha atoms, maintaining a high degree of structural and conformational similarity. The inhibition constants of 3CL-PR with the eight inhibitors shown in Table 3 were compared with the structural features for binding of the inhibitors in the active site (Figure 6). Five of the structures were determined in this work, and four were determined previously with their coordinates retrieved from the Protein DataBank.^8,26,27^ In all cases, the inhibitors are seen as covalent adducts (thiohemiacetals) of Cys_145_ bound in a channel on the exterior of the protein. It is noted that the 2F_0_-F_c_ density for the inhibitors, especially those of lower resolution structures (≥2.0 Å), is of lower quality due to the surface location of the inhibitors; the maps presented here are Polder maps (Figure 6), which show significantly more interpretable density because they reduce the density modification from bulk solvent models in standard maps/refinement. The P_1_ side chains of the nine 3CL-PR inhibitors all similarly occupy the S_1_ binding subsite in all structures shown in Figures 6, 7 and 8. **Nirmatrelvir** and **LL478** (**3**) have similar substituents in their P_4_–P_2_ groups, and these groups occupy comparable spaces in the S_4_–S_2_ subsites of 3CL-PR (top panel of Figure 6). Conserved hydrogen bonds are observed between the α-amide and α-carbonyl oxygen of Glu_166_ and the complementary carbonyl oxygen between the P_3_–P_2_ groups and the amide protons between the P_4_–P_3_ groups. These hydrogen bonds are found in all eight structures. For **LL478** (**3**), but not **Nirmatrelvir**, the carbonyl oxygen of the γ-amide group of Gln_189_ forms a hydrogen bond with the backbone amide proton between the P_3_–P_2_ groups, which is also present in the structures of 3CL-PR bound with **PF835231**, **VK13**, and **VK20**. The respective OpA and 2-pyridon-3-alanyl P_1_ side chains of **Nirmatrelvir** and **LL478** bind in the S_1_ subsite of 3CL-PR in an identical manner, and conserved hydrogen bonds are established between the amide groups of the P_1_ side chains and the side chains of Glu_166_ (Glu_166_–C–O^–^···H–N–P_1_) and His_163_ (His_163_ N–H···O=C–P_1_) and the α-carbonyl oxygen atom of Phe_140_. These hydrogen bonds are also observed for structures for all inhibitors with a 2-pyridon-3-alanyl P_1_ group (compounds **3–6**) as well as **PL-835231**. This confirms that the 2-pyridon-3-yl alanyl side chain is a suitable bioisostere of the obligate glutamine amino acid found at the P_1_ positions of native polyprotein substrates, while at the same time, these P_1_ groups are apparently also well accommodated by the widely open S_1_ binding subsite of cathepsin L.

The three heterocyclic-alanyl groups at the P_1_ position of inhibitors **6–8** are shown superimposed in the S_1_ site of 3CL-PR in Figure 7. Thiohemiacetals for all three inhibitors are evident, and the hydroxyl group of all three thiohemiacetals forms apparent hydrogen bonds with the side chain N-ε of His_163_. Hydrogen bonds are formed between the 2-pyridonyl group of **VK13** (**6**), the backbone carbonyl oxygen atom of Phe_140_, the side chain oxygen atoms of Ser_144_ and Glu_166_, and the side chain hydroxyl of Ser_144_.

Compounds **6** and **7** differ in structure only by the 2-oxo or 2-hydroxy substituent of the 2-pyridone of **6**. The pyridine of **BC787** (**7**) forms apparent hydrogen bonds with Phe_140_ and Glu_166_, but not Ser_144_, a hydrogen bond found with the 2-pyridone and OpA substituents. **BC787** shows a binding mode consistent with that of the other inhibitors in one of the two monomers of the asymmetric unit. However, unlike all other structures, the binding mode in the second monomer is unique. In this binding mode, which occurs with poor and difficult-to-model electron density, the inhibitor is covalently bound to Cys_145_, but all of the anticipated hydrogen bonds are not formed (Figure S12). Indeed, the inhibitor does not bind with the inhibitor side chains in the enzyme-substrate sites (P_1_ is not in S_1_, etc.), likely explaining the poor electron density. This is also reflected in the 2400-fold loss of potency of **7** (*K*_i_ = 6200 nM) compared to that of **6** (*K*_i_ = 2.6 nM). The 1,3-oxazole group of **VK20** (**8**) does not form hydrogen bonds with any of these residues but it does with His_163_. The smaller size of the 1,3-oxazole ring retracts its heteroatoms from the S_1_ site residues Phe_140_, Ser_144_, and Glu_166_, as there are no apparent hydrogen bonds donated from these residues. This absence of hydrogen bonding arising from its oxazole substituent is consistent with the lower potency of **8** (*K*_i_ = 124 nM) compared to Compound **6** (50-fold more potent) for which these three inhibitors are otherwise identical. Regardless, the oxazole group of inhibitor **8** constitutes a glutamine analogue since it potently binds to 3CL-PR, whereas peptide aldehyde inhibitors that feature a Phe group at the P_1_ position do not bind to 3CL-PR at all.

The P_2_ side chains of inhibitors **3–8** (the centered side chains pointing downward in Figures 6 and 7) are accommodated by the S_2_ protease subsite, in which valine and leucine are commonly found in peptide substrates for 3CL-PR, and both the leucine or cyclohexylalanine side chains adopt similar conformations for all six of our inhibitors, and are superimposable (Figure 7). The isopropyl groups of the P_2_ leucine groups are mobile, adopting a variety of conformations, as reflected by the observed poor electron density for some structures. Leucine, and especially the cyclohexyl-alanine side chains of **3** and **6**, occupy comparable space with the dimethyl-3-azabicyclo[3.1.0]hexane proline/leucine mimic of **Nirmatrelvir**.

The P_3_ valine of **3** is effectively superimposable with the *tert*-Leu group at the P_3_ position of **Nirmatrelvi**r. The N-terminal trifluoroacetamide group of **Nirmatrelvir** and the corresponding benzylcarbonyloxy-N-terminus of **3** extend into the S_3_ binding pocket. For the indo-2-yl-Leu or 4-methoxy-indo-2-yl-Leu groups at the P_3_-P_2_ positions of inhibitors **4**-**8**, the nitrogens and 2-carbonyl groups of the indoles are coplanar, and these heteroatoms form hydrogen bonds with the backbone carbonyl and amide nitrogen of Glu_166_ of 3CL-PR (Figures 6, 7 and 8). Other hydrogen bonding is observed between the carbonyl oxygen of the γ-amide of Gln_189_ and the α-amino group of the P_2_ Leu, but not for all inhibitors for which we have obtained crystal structures. For inhibitors **3**, **6**, **8**, and **PF-835231**, the α-amino groups of the P_2_ Leu are in close contact with the γ-carbonyl oxygens of Gln_189_ such that hydrogen bonds are formed for these bound inhibitors (Figure 8). This H-bond is not available for **Nirmatrelvir** because of the azabicyclo ring system. Inhibitors **6**, **8**, and **PF-835231** all have 4-methoxy-indole groups at their P_3_ positions and form these hydrogen bonds, while inhibitors **AC115**, **4**, and **5** have an unsubstituted P_3_ indole but do not form this hydrogen bond (Figure 8). The 4-methoxy groups of the indoles of **6**, **8**, and **PF-835231** effect an apparent, common interaction with 3CL-PR that positions the carbonyl oxygen of Gln_189_ to form a hydrogen bond in a manner not available to the substitution-free indole groups of inhibitors **4** and **5**. As inhibitors **4** and **6** differ in structure only by the 4-methoxy group, we propose that the 15-fold increase in inhibitory potency of **6** over **4** is due to the establishment of this hydrogen bond. This hydrogen bond is also present in the 3CL-PR-**3** structure in which a Cbz-Val-Leu dipeptide comprises the P_4_–P_2_ group.

## Summary

Potent dual-target peptidomimetic aldehyde inhibitors of both human cathepsin Land SARS-CoV-2 3CL protease were generated, characterized, and shown to exist as free aldehydes and not δ-lactols in polar solvents. The inhibitors contain examples of three new side chains at the P_1_ peptide position: 2-pyridon-3-alanyl, 3-pyridin-alanyl, and 1,3-oxazo-4-alanyl substituents, and are housed within several peptidomimetic scaffolds similar to those found in three clinical candidates for the treatment of COVID-19, **Nirmatrelvir**, **PF-835231**, and **AC1115**. The P_1_ side chains are all accommodated in the S_1_ subsite of 3CL-PR, the best of which, the 2-pyridon-3-alanyl group, establishes the same hydrogen bonds found in the 2-oxo-pyrrolidin-3-alanyl of the clinical candidates and therefore act as isosteres of the glutamine found in the native substrate. The P_3_ and P_2_ side chains all occupy similar subsites in 3CL-PR. The cyclohexyl-Ala group of **LL-478** overlaps well with the dimethyl-3-azabicyclo[3.1.0]hexane group of **Nirmatrelvir**, but the cyclohexyl-Ala group allows the formation of a hydrogen bond of the backbone amide group of **LL-478** with Gln_189_, which is not the case for **Nirmatrelvir**. This difference could, in part, account for the dual-target activity of **LL-478** which is absent in **Nirmatrelvir**. **AC1115** and its homologue **VK-13** also showed nearly identical modes of binding to 3CL-PR, which is reflected in their identical values of *K*_i_. A potential binding advantage for both **VK-13** and **VK-20** is that the 4-methoxy group of the indole group, absent in **AC1115**, also enables the apparent hydrogen bond established between the backbone amide group of the two inhibitors and Gln_189_. The anti-CoV-2 activities found in these dual-target inhibitors approach those of the clinical candidates. The *K*_i_ values for hCatL were well correlated with the anti-CoV-2 activities (EC_50_ values) for a subclass of these new inhibitors, suggesting that the action of hCatL during the onset of infection is more sensitive to inhibition than 3CL protease in the permissive Vero E6 cell line used. The dual-target inhibitors described here that contain novel heterocyclic isosteres of glutamine and OpA provide a second generation of protease inhibitors with potent anti-CoV-2 activity. Derivatives of these inhibitors will likely lead to therapeutic agents for this lingering disease because of the significant advantage that their dual-target activities will surmount the inevitable development of drug resistance arising from point mutations within 3CL protease.

## Supporting Information Available

Plots of inhibitors vs variable substrates for 3CL-PR and hCatL, Cheng–Prusoff plots for time-dependent inhibition of 3CL-PR, hCatL and hCat B, anti-CoV-2 activity in infected cell lines, analytical data (NMR, HPLC, and mass spectrometric data) for synthetic compounds, SDS-PAGE gels and mass spectrometry data for the purification of human cathepsin L, and additional structural data for inhibitors bound to 3CL-PR (PDF)

## Abbreviations

3CL-PR: 3-chymotrypsin-like protease (or main protease)
Abz: 2-aminobenzoic acid
AMC: 7-amino-4-methylcoumarin
Cbz and Z: carbobenzyloxycarbonyl
CHAPS: 3-[(3-cholamidopropyl)dimethylammonio]-1-propanesulfonate
CP: cysteine protease
DIPEA: di-isopropylethylamine
Dnp: dinitrophenol
DMF: dimethylformamide
DCM: dichloromethane
DMF: dimethylformamide
DTT: dithiothreitol
Edans: (5-((2-aminoethyl)amino)naphthalene-1-sulfonyl
EDTA: ethylenediaminetetraacetic acid
GST: glutathione transferase
HATU: hexafluorophosphate azabenzotriazole tetramethyl uronium
His_6_: hexa-histidine; human rhinovirus 3CL protease, HRV-3CL
IPTG: isopropyl β-d-1-thiogalactopyranoside
NMR: nuclear magnetic resonance
Opa: 2-oxo-pyrrolidin-3-alanyl
RFUs: relative fluorescence units
TFA: trifluoroacetic acid
