# Supplementary material for: Dual Inhibitors of SARS-CoV-2 3CL Protease and Human Cathepsin L Containing Glutamine Isosteres Are Anti-CoV-2 Agents
Source: J Am Chem Soc. 2025 Jan 2;147(2):1631–48. doi: 10.1021/jacs.4c11620 (PMC11744766; doi:10.1021/jacs.4c11620)
Supplement: Supplementary file 1 — ja4c11620_si_001.pdf [file ja4c11620_si_001.pdf]

SUPPORTING INFORMATION

Dual Inhibitors of SARS-CoV-2 3CL Protease and Human Cathepsin L Containing Glutamine Isosteres Are Anti-CoV-2 Agents

Vivek Kumar,[1,#] Jiyun Zhu[1,#,§], Bala C. Chenna[1,#], Zoe A. Hoffpauir[2],[#] Andrew Rademacher[1], Ashley M. Rogers[1], Chien-Te Tseng[3], Aleksandra Drelich[3], Sharfa Farzandh[2], Audrey L. Lamb[2,*], and Thomas D. Meek[1,*]

[1]Department of Biochemistry and Biophysics, Texas A&M University, 301 Old Main Drive, College Station, Texas 77845, United States

[2]Department of Chemistry, University of Texas at San Antonio, 1 UTSA Circle, San Antonio, Texas 78249, United States

[3]Department of Microbiology & Immunology Centers for Biodefense and Emerging Diseases, The University of Texas Medical Branch at Galveston, 301 University Boulevard, Galveston, Texas 77555, United States

*Emails: Thomas.Meek@ag.tamu.edu; Audrey.lamb@utsa.edu

,

Present address:

[§]J. Z.: 300 Pasteur Drive, Edwards Boulevard, Department of Pathology, Stanford University, Stanford, CA, 94305-5324, United States

*Author to whom correspondence should be addressed

Running Title: Dual Inhibitors of SARS-CoV-2 3CL Protease and Human Cathepsin L

Table of Contents

Figures… 2-30

FIGURES

Compound 3 (LL-478).

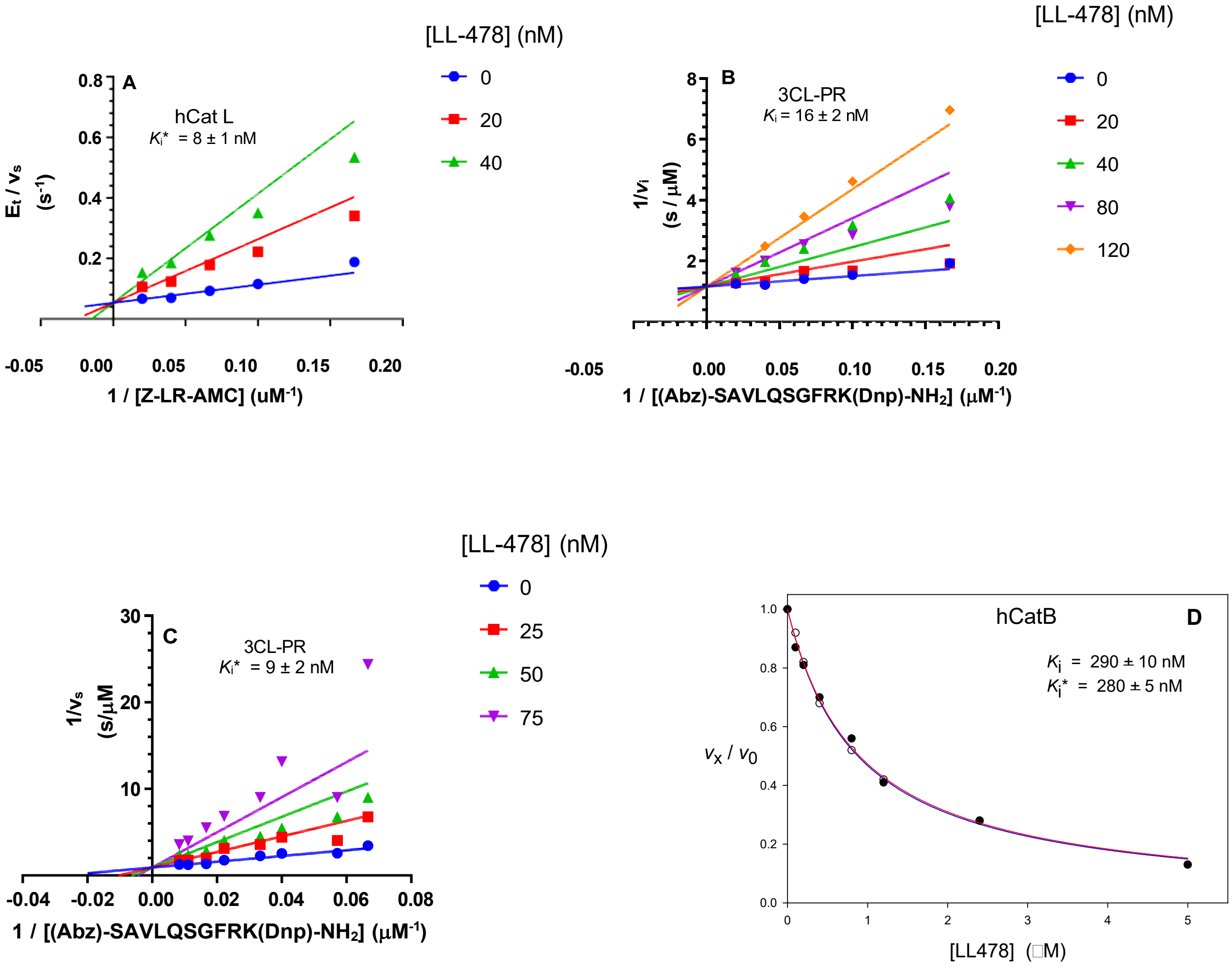

Figure S1. (**A**) Apparent competitive inhibition of human cathepsin L by **LL478** vs. Cbz-Leu-Arg-AMC at 15-25 min after reaction initiation plotted as [E_t] / v_s vs. 1/(substrate), (**B**), apparent competitive inhibition of 3CL-PR by **LL478** vs. Abz-SAVQSGFRK(Dnp)-NH_2 at < 10 min plotted as 1 / v_s vs. 1/(substrate); (**C**) same as **B** except incubation time > 20 min. Lines drawn through the experimental data were from fitting to eq 7. (**D**) Cheng-Prusoff plot of fractional inhibition of human cathepsin B by **LL478** plotted as normalized initial rates v_i/v_0 (t = 0-3 min) and steady-state rates v_s/v_0 (t = 27-30 min) vs. [**LL478**] at a fixed concentration (50 µM) of the substrate Cbz-Leu-Arg-AMC. Curves drawn through the experimental are from fitting data to eq 4 or 5. Values of K_i and K_i* are those obtained, respectively, from initial (v_i) or steady-state (v_s) rates in the presence of inhibitor.

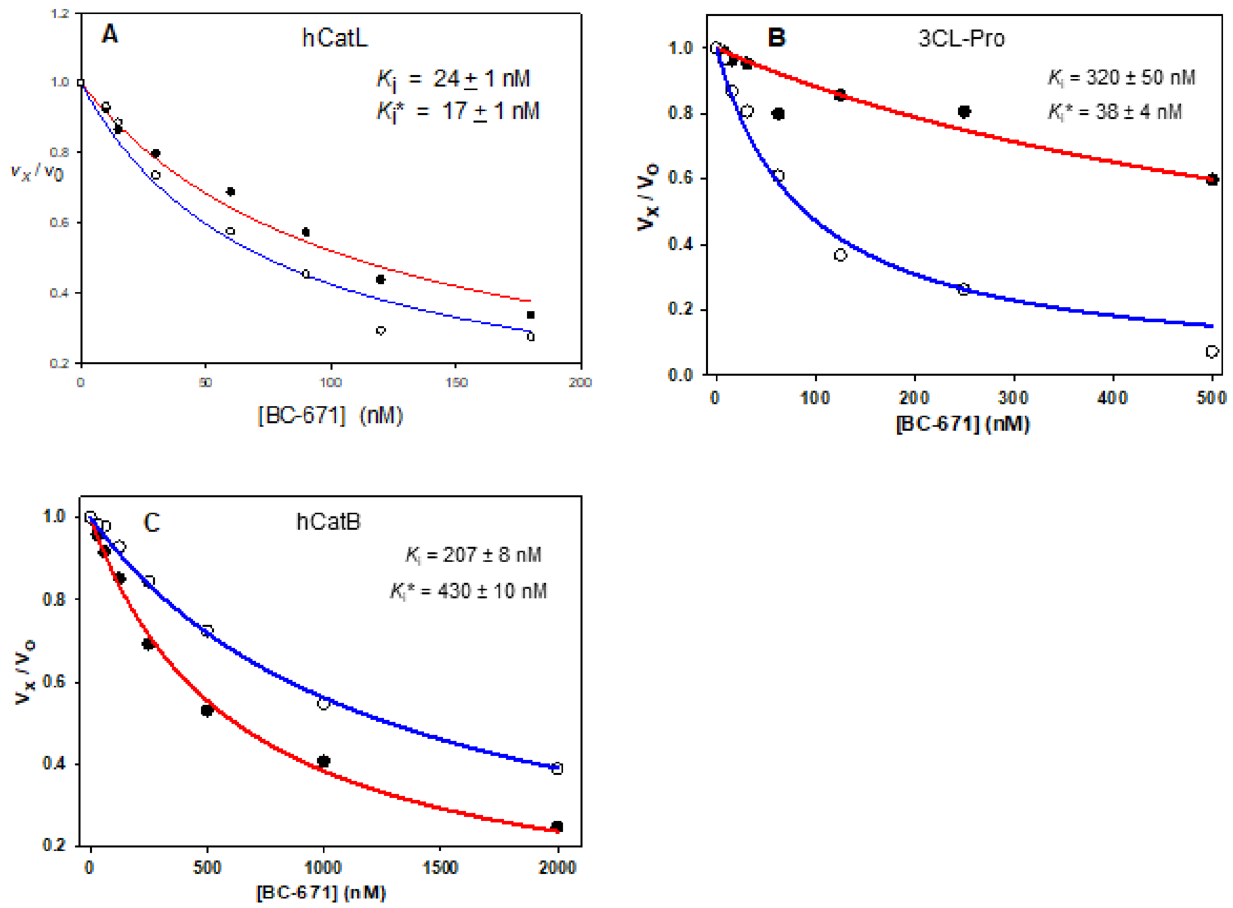

Figure S2. Cheng-Prusoff plot of fractional inhibition of human cathepsin L **(A)** and human cathepsin B **(C)** by **BC671** plotted as normalized initial rates v_i/v_0 (t = 0-3 min) and steady-state rates v_s/v_0 (t = 27-30 min) vs. [**BC671**] at a fixed concentration (10 μM for hCatL and 50 μM for hCatB) of the substrate Cbz-Leu-Arg-AMC. **(B)** Cheng-Prusoff plot of fractional inhibition of 3CL-PR by **BC-671** plotted as normalized initial rates v_i/v_0 (t = 0-3 min) and steady-state rates v_s/v_0 (t = 27-30 min) vs. [**BC-671**] at a fixed concentration (50 μM) of the substrate Dabcyl-KTSAVLQSGFRKME-Edans. Curves drawn through the experiment are from fitting data to eq 4 or 5. Values of K_i and K_i* are those obtained, respectively, from initial (v_i) or steady-state (v_s) rates in the presence of inhibitor.

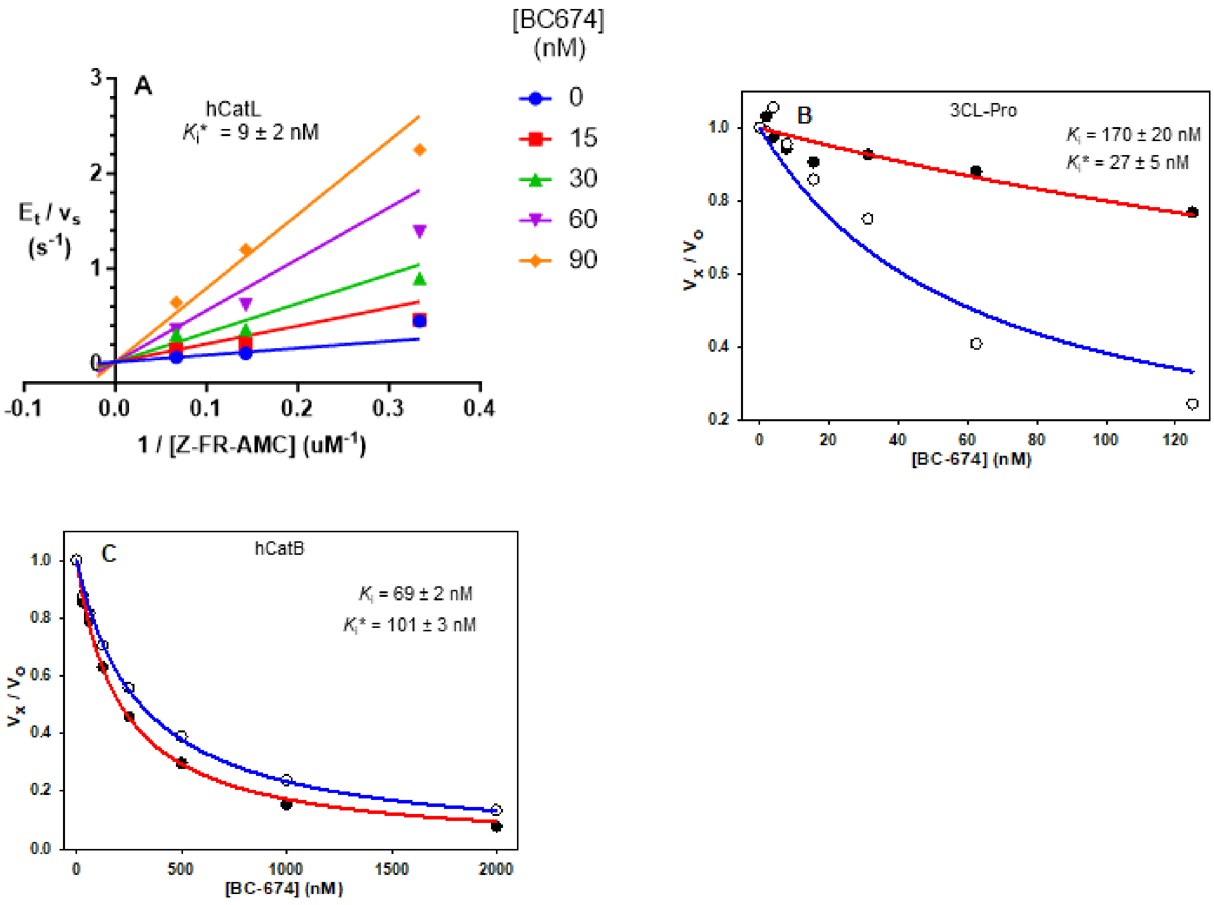

Figure S3. (**A**) Apparent competitive inhibition of human cathepsin L by **BC674** vs. Cbz-Phe-Arg-AMC at 15-25 min after reaction initiation, plotted as $[E_t]$ / v_s vs. 1/(substrate) **(B)** Cheng-Prusoff plot of fractional inhibition of 3CL-PR by **BC674** plotted as normalized initial rates v_i / v_0 (t = 0-3 min) and steady-state rates v_s / v_0 (t = 27-30 min) vs. [**BC674**] at a fixed concentration (50 μM) of the substrate Dabcyl-KTSAVLQSGFRKME-Edans. Curves drawn through the experiment are from fitting data to eq 4 or 5. (**C**) Cheng-Prusoff plot of fractional inhibition of human cathepsin B by **BC674** plotted as normalized initial rates v_i/v_0 (t = 0-3 min) and steady-state rates v_s/v_0 (t = 27-30 min) vs. [**BC674**] at a fixed concentration (50 μM) of the substrate Cbz-Leu-Arg-AMC. Curves drawn through the experiment are from fitting data to eq 4 or 5. Values of K_i and K_i* are those obtained, respectively, from initial (v_i) or steady-state (v_s) rates in the presence of inhibitor.

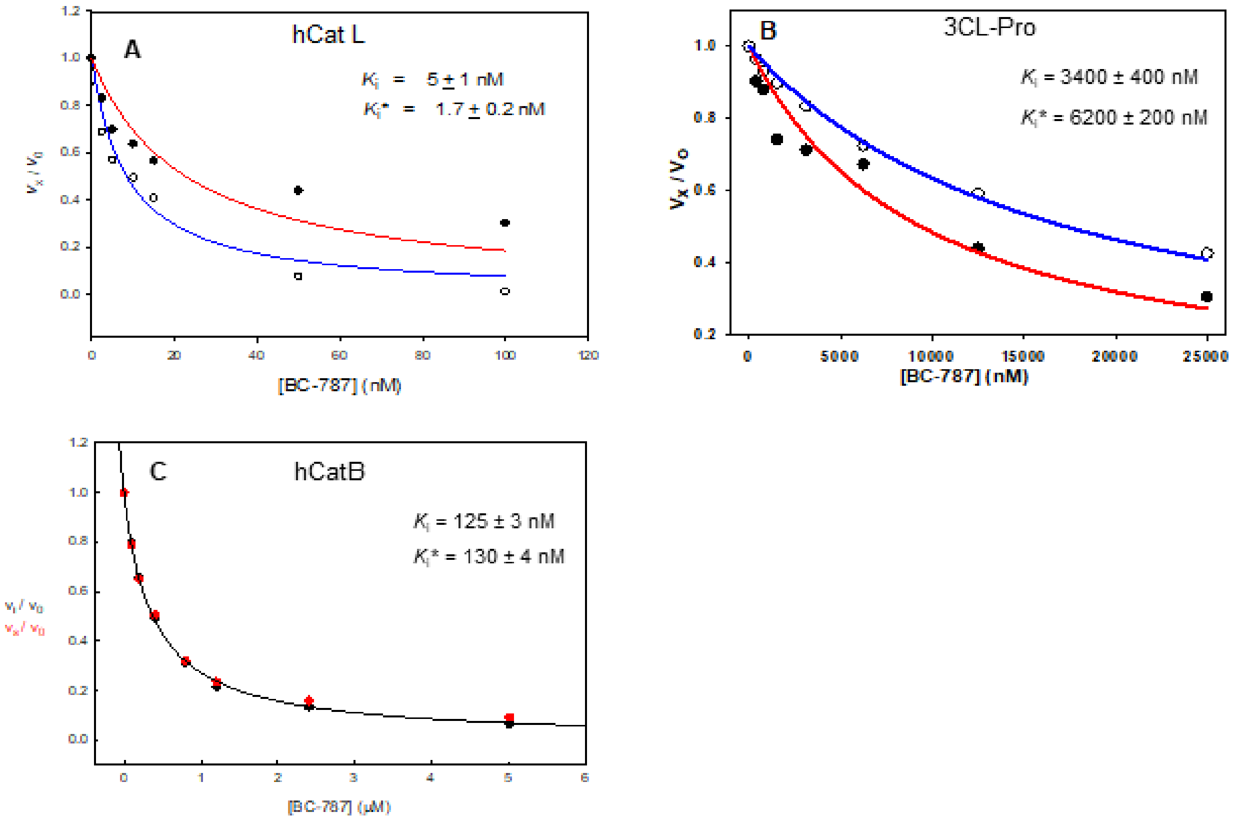

Figure S4. Cheng-Prusoff plot of fractional inhibition of human cathepsin L **(A)** and human Cathepsin B **(C)** by **BC787** plotted as normalized initial rates v_i/v_0 (t = 0-3 min) and steady-state rates v_s/v_0 (t = 27-30 min) vs. [**BC787**] at a fixed concentration (10 μM for hCatL and 50 μM for hCatB) of the substrate Cbz-Leu-Arg-AMC. **(B)** Cheng-Prusoff plot of fractional inhibition of 3CL-PR by **BC787** plotted as normalized initial rates v_i/v_0 (t = 0-3 min) and steady-state rates v_s/v_0 (t = 27-30 min) vs. [**BC787**] at a fixed concentration (50 μM) of the substrate Dabcyl-KTSAVLQSGFRKME-Edans. Curves drawn through the experiment are from fitting data to eq 4 or 5. Values of K_i and K_i* are those obtained, respectively, from initial (v_i) or steady-state (v_s) rates in the presence of inhibitor.

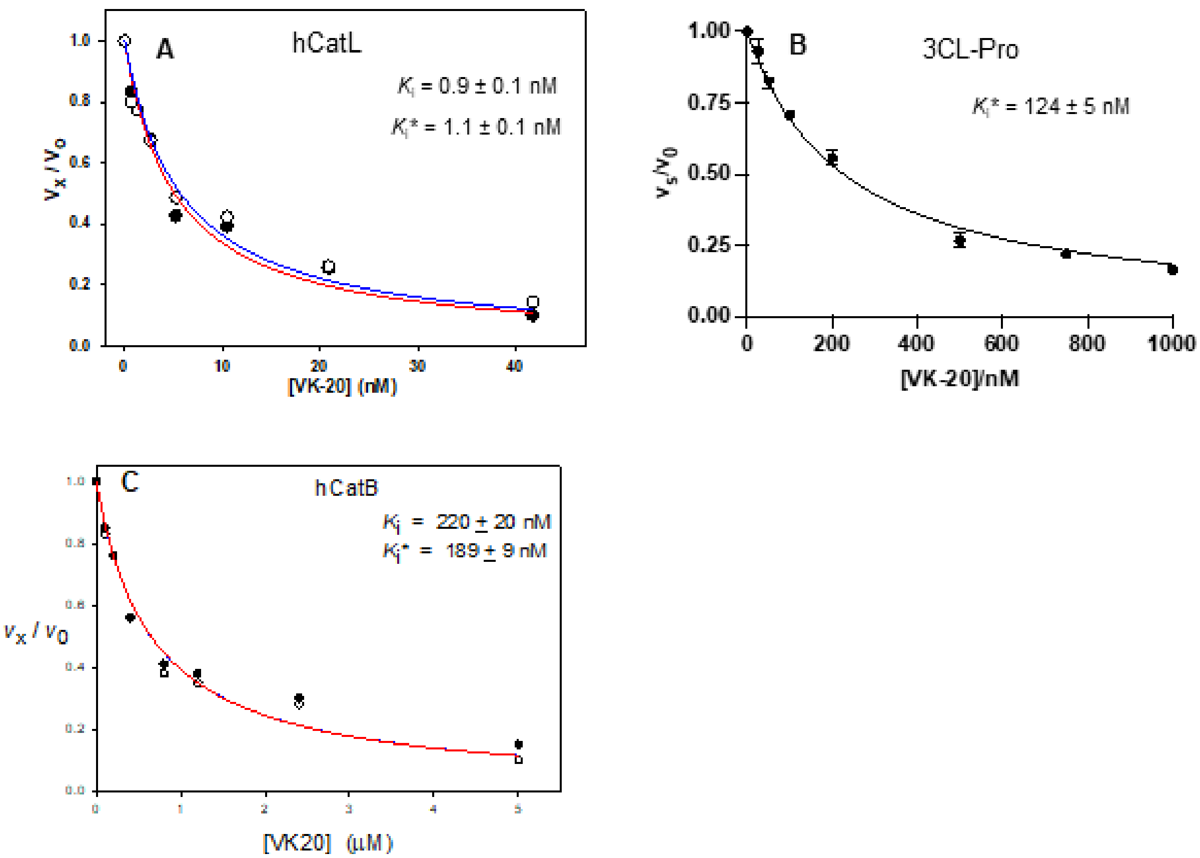

Figure S5. Cheng-Prusoff plot of fractional inhibition of human cathepsin L (**A**) and human cathepsin B (**C**) by **VK20** plotted as normalized initial rates v_i/v_0 (t = 0-3 min) and steady-state rates v_s/v_0 (t = 27-30 min) vs. [**VK20**] at a fixed concentration (10 μM for hCatL and 50 μM for hCatB) of the substrate Cbz-Leu-Arg-AMC. (**B**) Cheng-Prusoff plot of fractional inhibition of 3CL-PR by **VK20** plotted as normalized steady-state rates v_s/v_0 (t = 17-20 min) vs. [**VK20**] at a fixed concentration (50 μM) of the substrate Abz-SAVLQSGFRK(Dnp)-NH_2. Curves drawn through the experiment are from fitting data to eq 4 or 5. Values of K_i and K_i* are those obtained, respectively, from initial (v_i) or steady-state (v_s) rates in the presence of inhibitor.

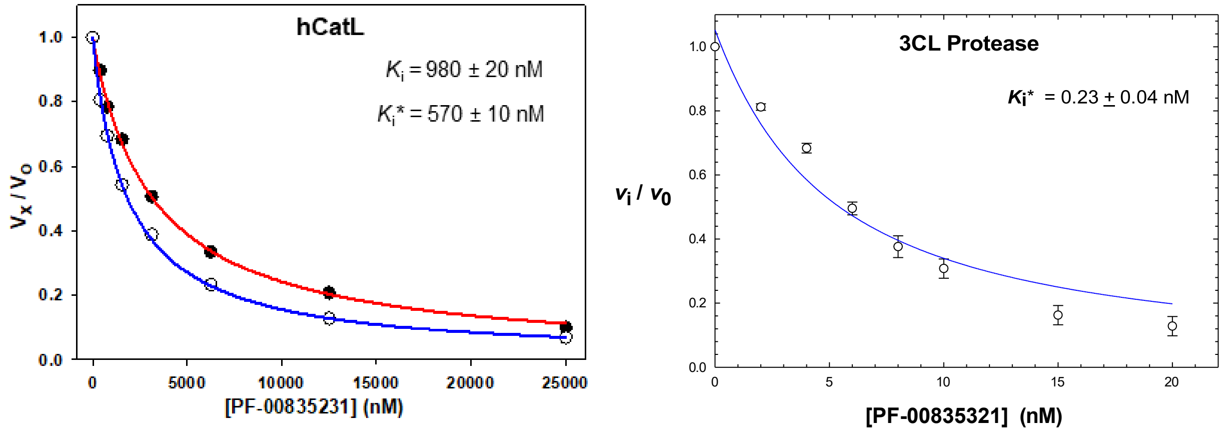

Figure S6. (**Left**) Cheng-Prusoff plot of fractional inhibition of human cathepsin L by **PF-835231** plotted as normalized initial rates v_i/v_0 (t = 0-3 min) and steady-state rates v_s/v_0 (t = 27-30 min) at a fixed concentration (50 μM) of the substrate Cbz-Leu-Arg-AMC. Curves drawn through the experiment are from fitting data to eq 4 or 5. Values of K_i and K_i* are those obtained, respectively, from initial (v_i) or steady-state (v_s) rates in the presence of inhibitor. (**Right**) Plot of fractional inhibition 3CL-PR by **PF-835231** plotted as normalized steady-state rates v_s/v_0 (t = 17-20 min) at a fixed concentration of Abz-SAVLQSGFRK(Dnp)-NH_2 and 1 nM enzyme. Curves drawn

through the experimental are from fitting data to eq 6 from which the K_i* value was obtained.

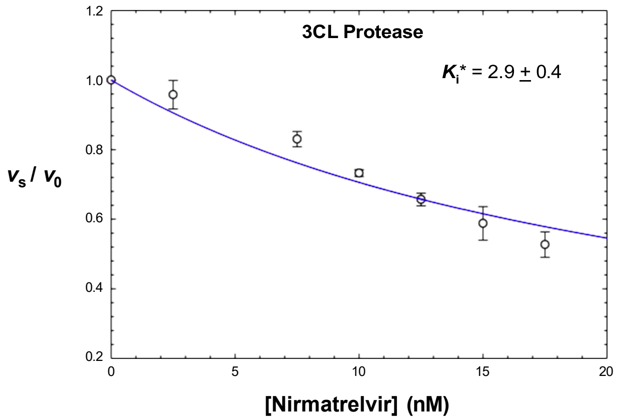

Figure S7. Plot of fractional inhibition of 3CL-PR by **Nirmatrelvir** plotted as normalized steady-state rates v_s/v_0 (t = 17-20 min) at a fixed concentration of Abz-SAVLQSGFRK(Dnp)-NH_2 and 1 nM enzyme. Curves drawn through the experimental are from fitting data to eq 6 from which the K_i* value was obtained.

Table S1. Anti-CoV-2 Data for Self-Masked Aldehyde Inhibitors in SARS-CoV-2-Infected Vero E6 Cells.*

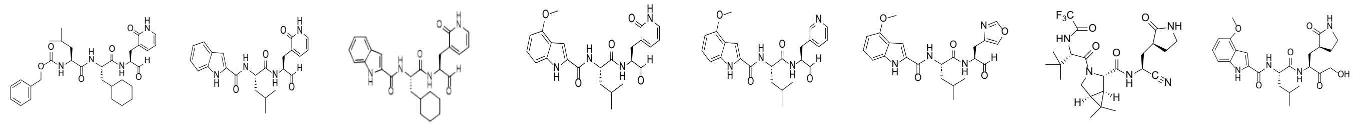

	LL478	BC671	BC674	VK13	BC787	VK20	Nirmatrelvir	PF-835231
	$K_{i^*\ CatL}$ = 9 nM	$K_{i^*\ CatL}$ = 17 nM	$K_{i^*\ CatL}$ = 7 nM	$K_{i^*\ CatL}$ = 0.55 nM	$K_{i^*\ CatL}$ = 1.7 nM	$K_{i^*\ CatL}$ = 0.9 nM	$K_{i\ CatL}$ > 100 μM	$K_{i^*\ CatL}$ = 0.74 nM
	$K_{i^*\ 3CL\text{-}PR}$ = 9 nM	$K_{i^*\ 3CL\text{-}PR}$ = 38 nM	$K_{i^*\ 3CL\text{-}PR}$ = 27 nM	$K_{i^*\ 3CL\text{-}PR}$ = 2.6 nM	$K_{i\ 3CL\text{-}PR}$ = 6200 nM	$K_{i^*\ 3CL\text{-}PR}$ = 124 nM	$K_{i^*\ 3CL\text{-}PR}$ = 2.9 nM	$K_{i^*\ 3CL\text{-}PR}$ = 0.23 r

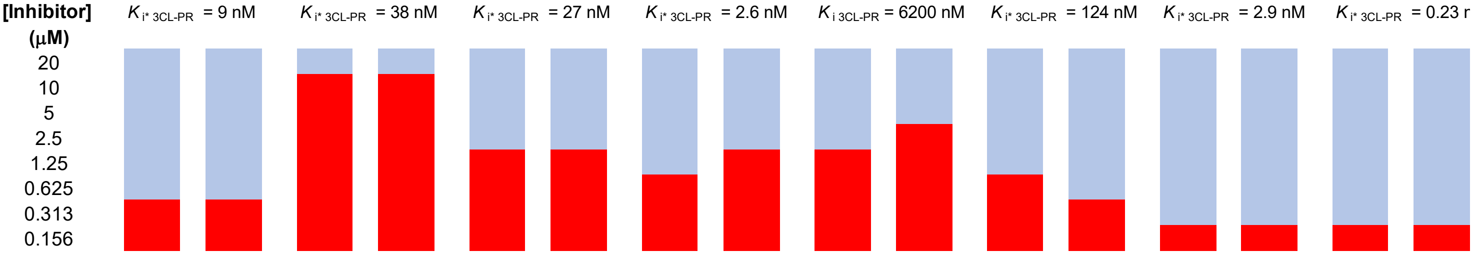

*Samples contained 1% (v/v) DMSO and 2 μM CP-100356. Red indicates the presence of the cytopathic effect of SARS-CoV-2 while blue indicates its absence.

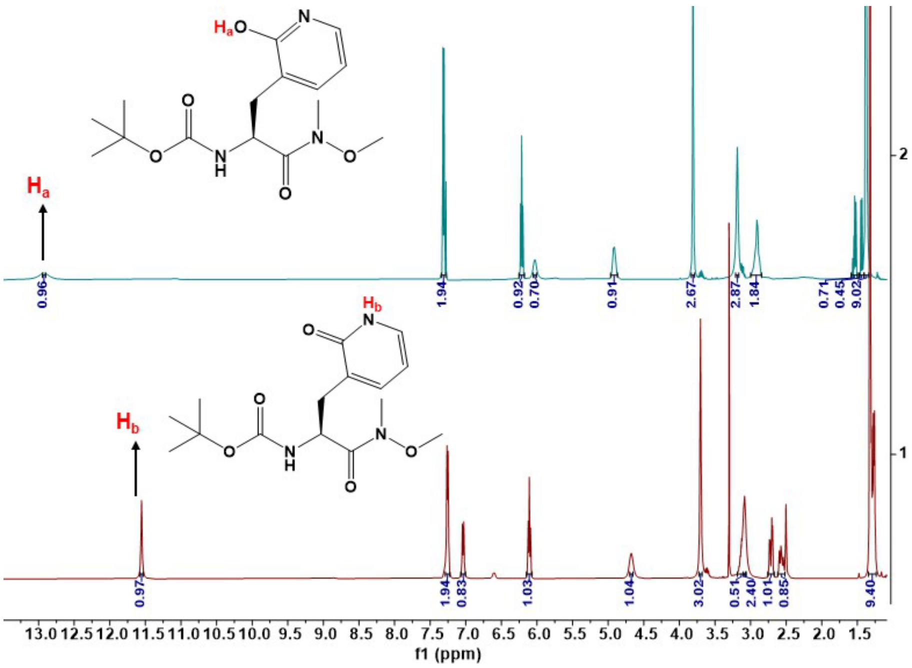

Figure S8: ^{1}H NMR spectra of compound **S2a**/**S2a1** obtained in $CDCl_3$ (Top) and DMSO-d_6 (Bottom). The different chemical shifts of the proton attached to either the oxygen (H_a) or the nitrogen (H_b) of the heterocyclic group demonstrated the lactim (**S2a1**) and lactam (**S2a**) tautomers are the respective predominate species in the non-polar and polar solvent.

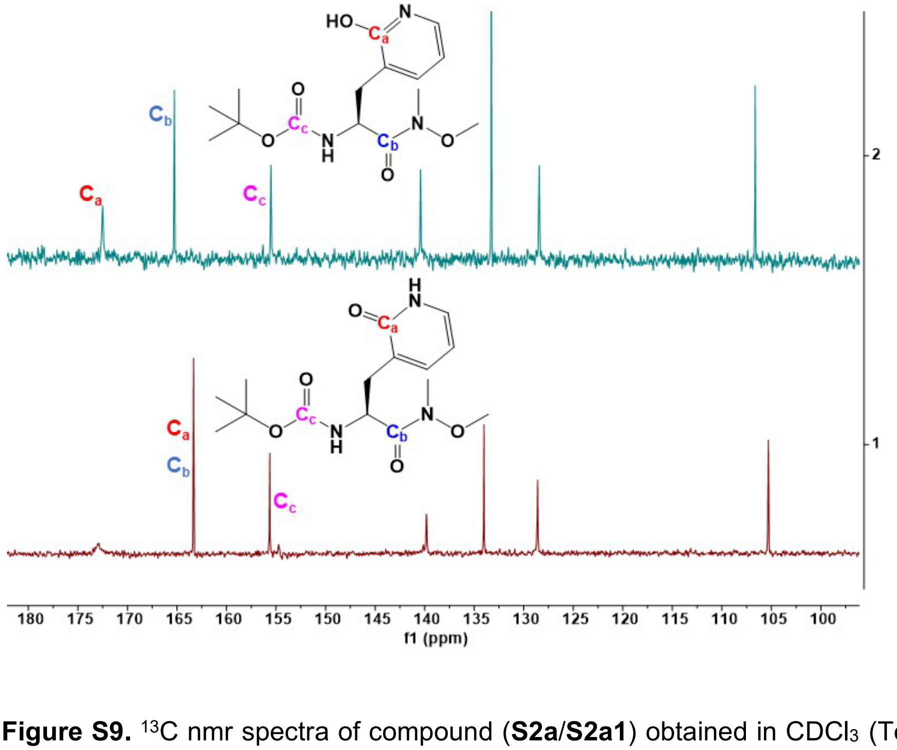

Figure S9. ^{13}C nmr spectra of compound (**S2a**/**S2a1**) obtained in $CDCl_3$ (Top) and DMSO-d_6 (Bottom). with three carbon atoms indicated (C_a-C_c). The chemical shifts of the lactam carbon (C_a) in DMSO-D_6 and more downfield lactim carbon in

CDCl$_3$ suggest lactam and lactim tautomers respectively. C$_c$ is Boc carbon and C$_b$ is Weinreb amide carbon shown in both spectra.

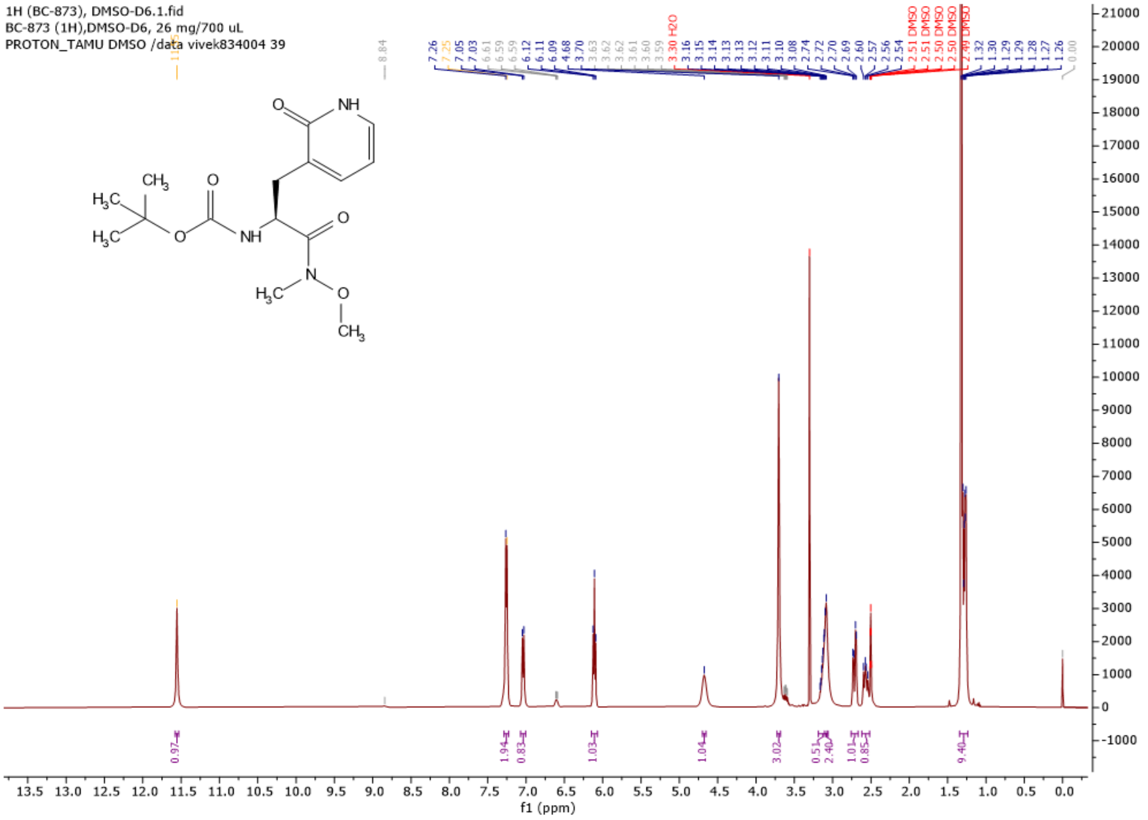

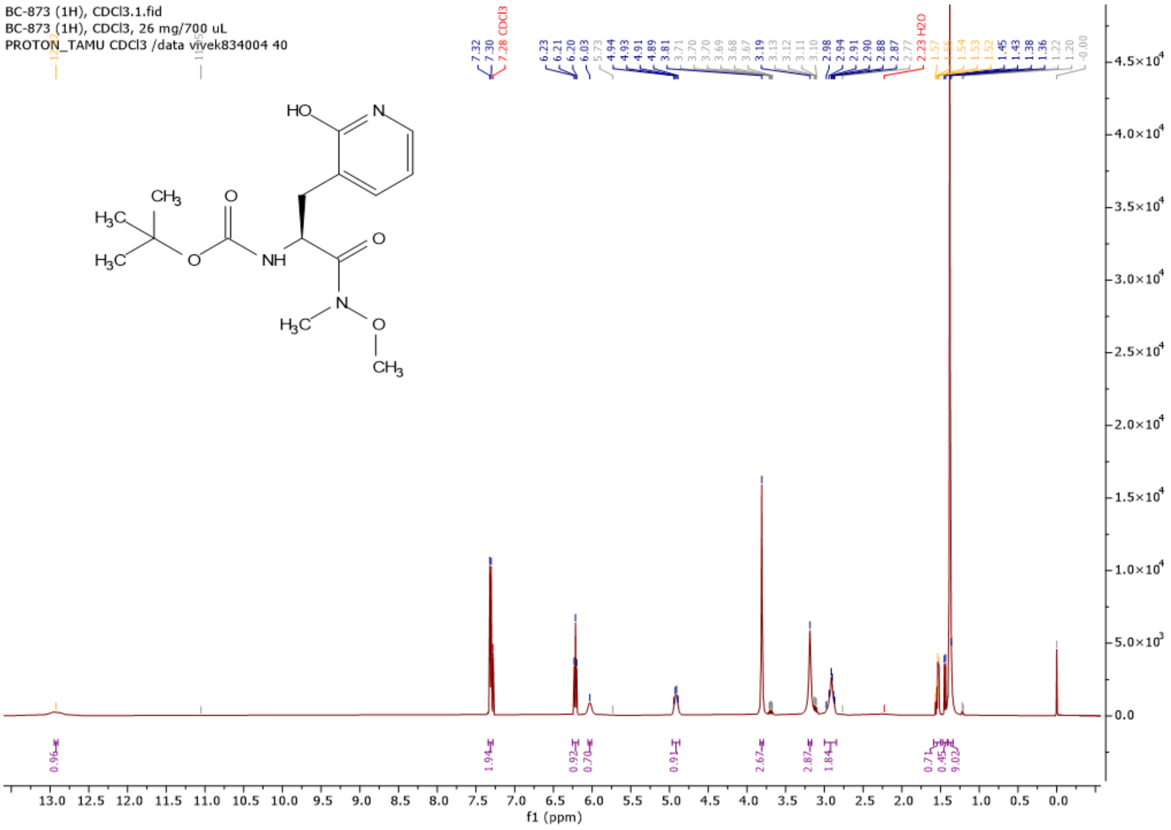

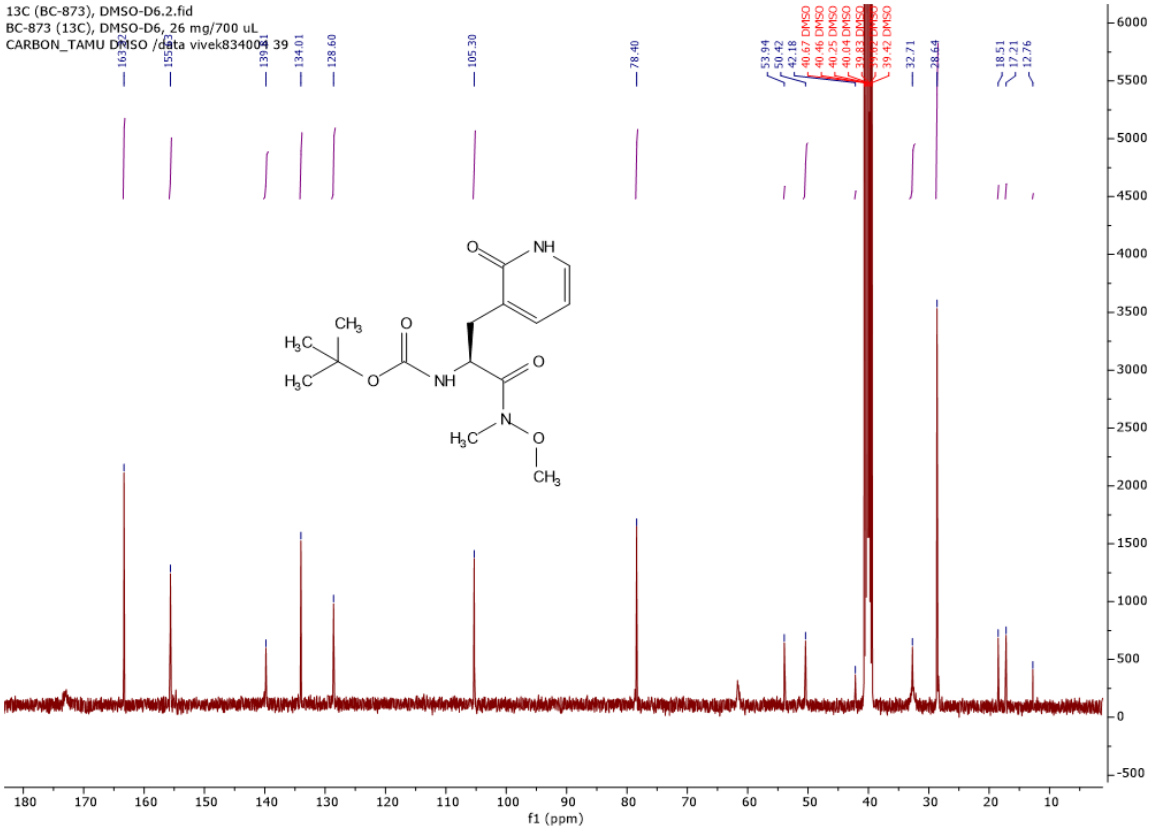
13C (BC-873), DMSO-D6.2.fid
BC-873 (13C), DMSO-D6, 26 mg/700 uL
CARBON_TAMU DMSO /data vivek834004 39
134.01
128.60
105.30
78.40
53.94
50.42
42.18
40.67 DMSO
40.46 DMSO
40.25 DMSO
40.04 DMSO
39.83 DMSO
39.42 DMSO
32.71
18.51
17.21
12.76
O
NH
CH3
H3C
H3C
O
NH
O
N
H3C
O
CH3
f1 (ppm)

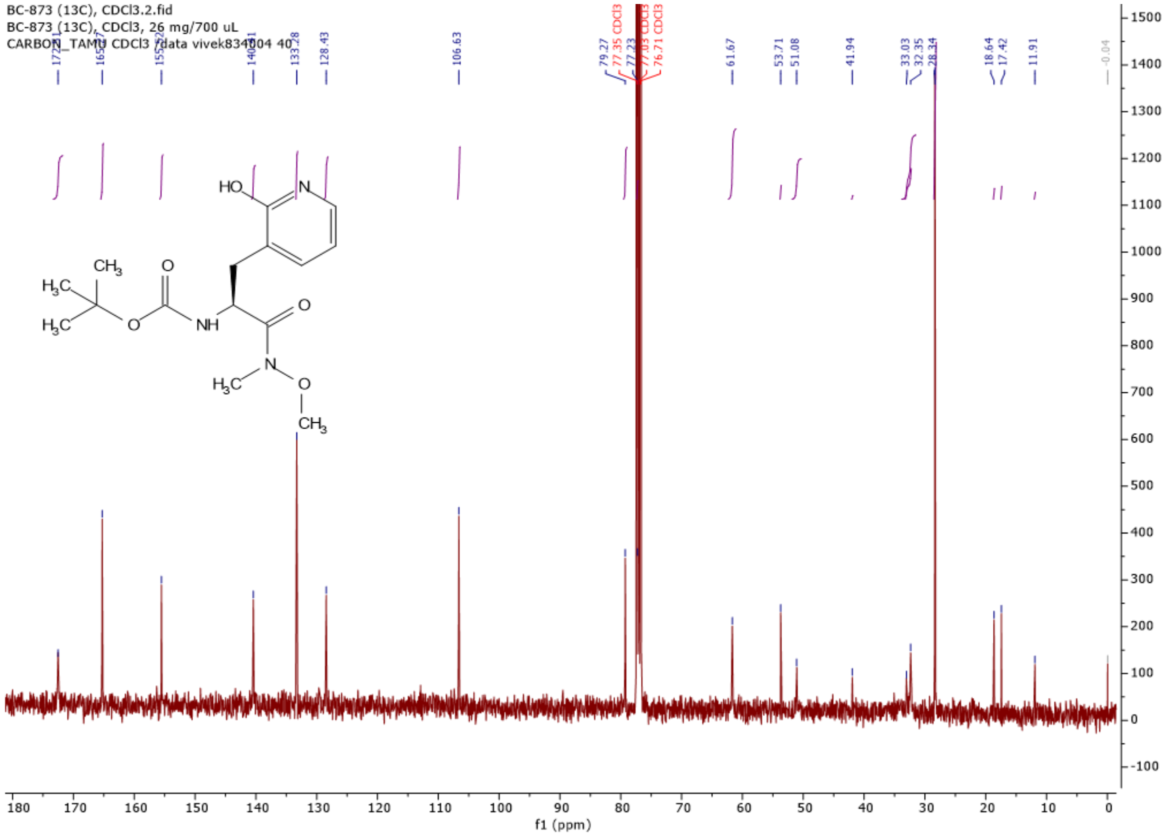
BC-873 (13C), CDCl3.2.fid
BC-873 (13C), CDCl3, 26 mg/700 uL
CARBON_TAMU CDCl3 /data vivek834004 40
133.28
128.43
106.63
79.27
77.35 CDCl3
77.23
76.71 CDCl3
61.67
53.71
51.08
41.94
33.03
32.35
18.64
17.42
11.91
-0.04
HO
N
CH3
H3C
H3C
O
NH
O
N
H3C
O
CH3
f1 (ppm)

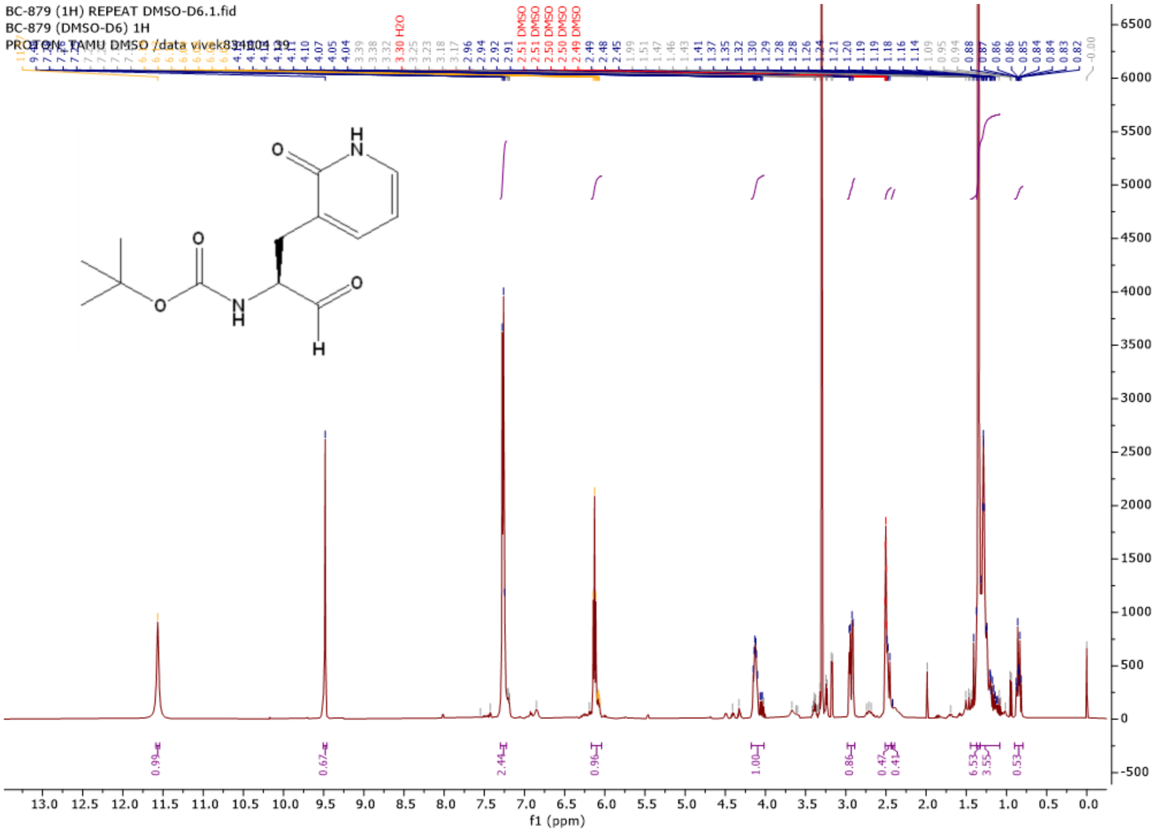
BC-879 (1H) REPEAT DMSO-D6.1.fid
BC-879 (DMSO-D6) 1H
PROTON_TAMU DMSO /data vivek834004 29
f1 (ppm)

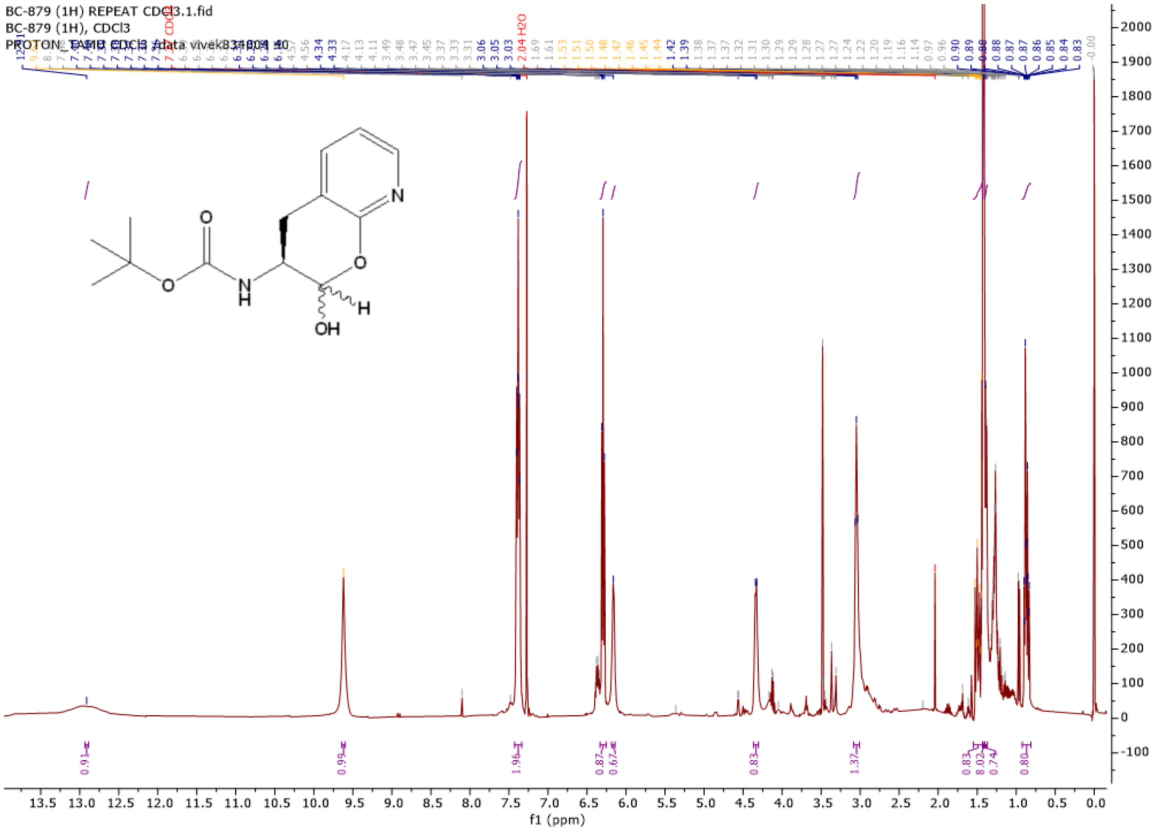
BC-879 (1H) REPEAT CDCl3.1.fid
BC-879 (1H), CDCl3
PROTON_TAMU CDCl3 /data vivek834004 40
f1 (ppm)

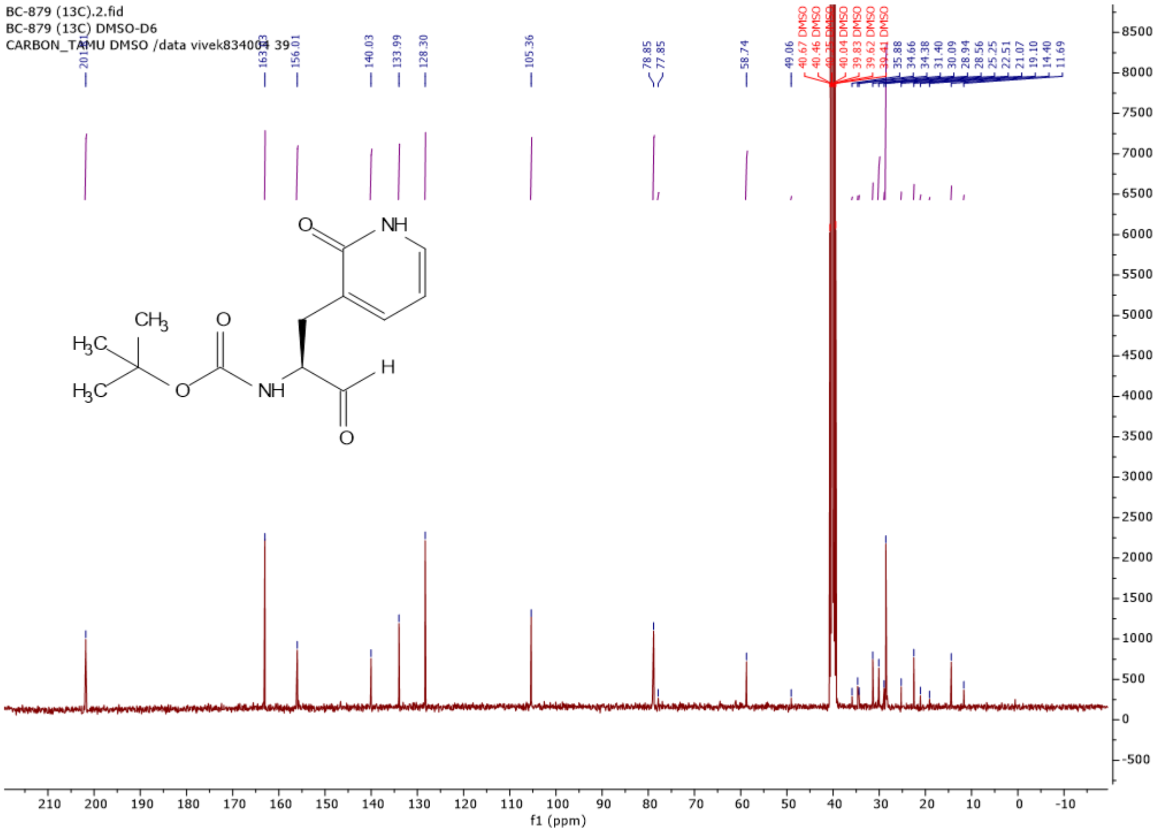
BC-879 (13C).2.fid
BC-879 (13C) DMSO-D6
CARBON_TAMU DMSO /data vivek834004 39
f1 (ppm)

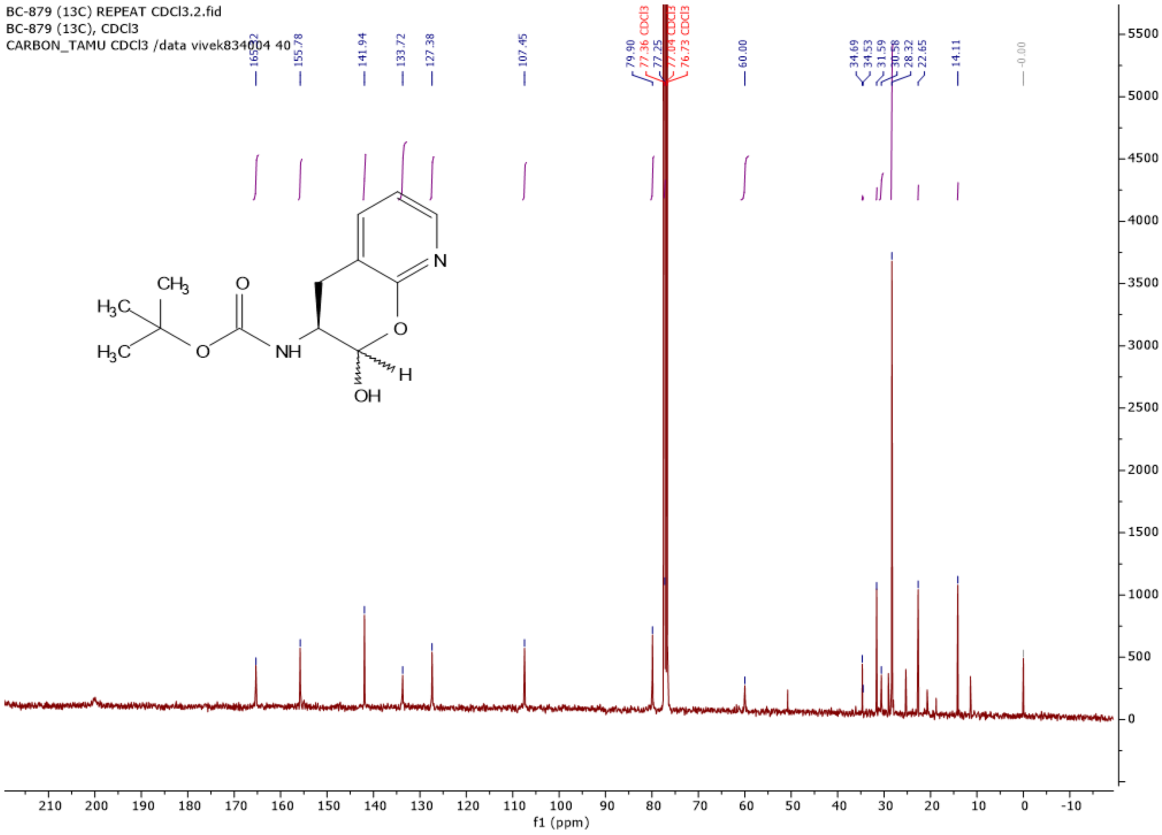
BC-879 (13C) REPEAT CDCl3.2.fid
BC-879 (13C), CDCl3
CARBON_TAMU CDCl3 /data vivek834004 40
f1 (ppm)

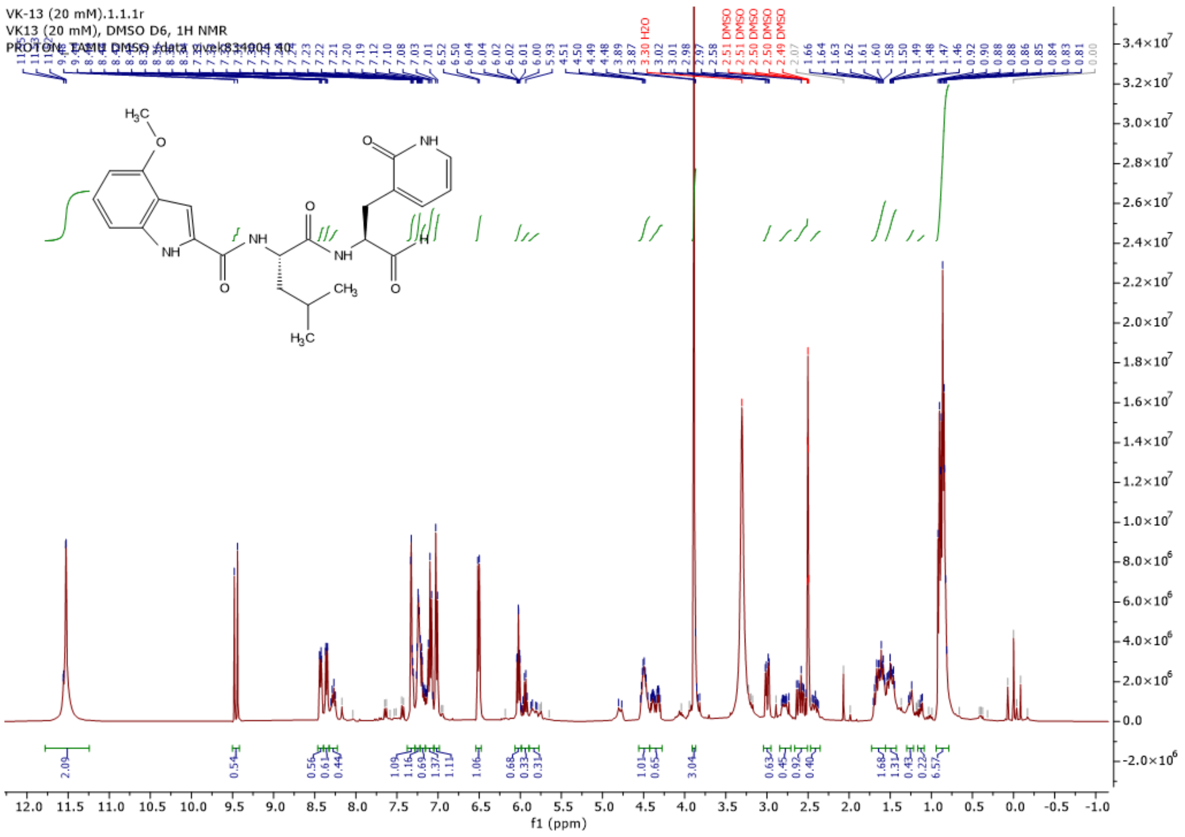

VK-13 (20 mM).1.1.1r
VK13 (20 mM), DMSO D6, 1H NMR
3.30 H2O
2.51 DMSO
2.51 DMSO
2.50 DMSO
2.50 DMSO
2.49 DMSO
2.09
0.54
1.06
3.04
6.57
f1 (ppm)

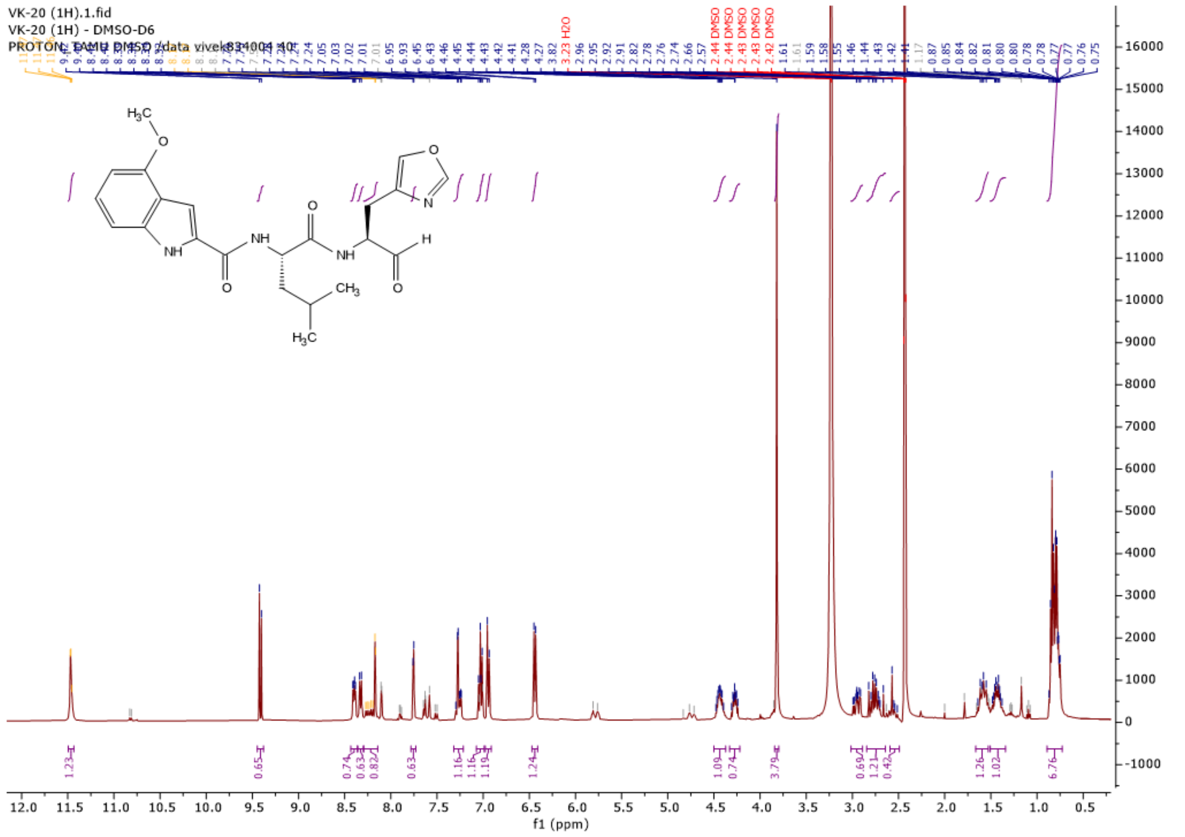

VK-20 (1H).1.fid
VK-20 (1H) - DMSO-D6
3.23 H2O
2.44 DMSO
2.44 DMSO
2.43 DMSO
2.43 DMSO
2.42 DMSO
1.23
0.65
1.24
3.79
6.76
f1 (ppm)

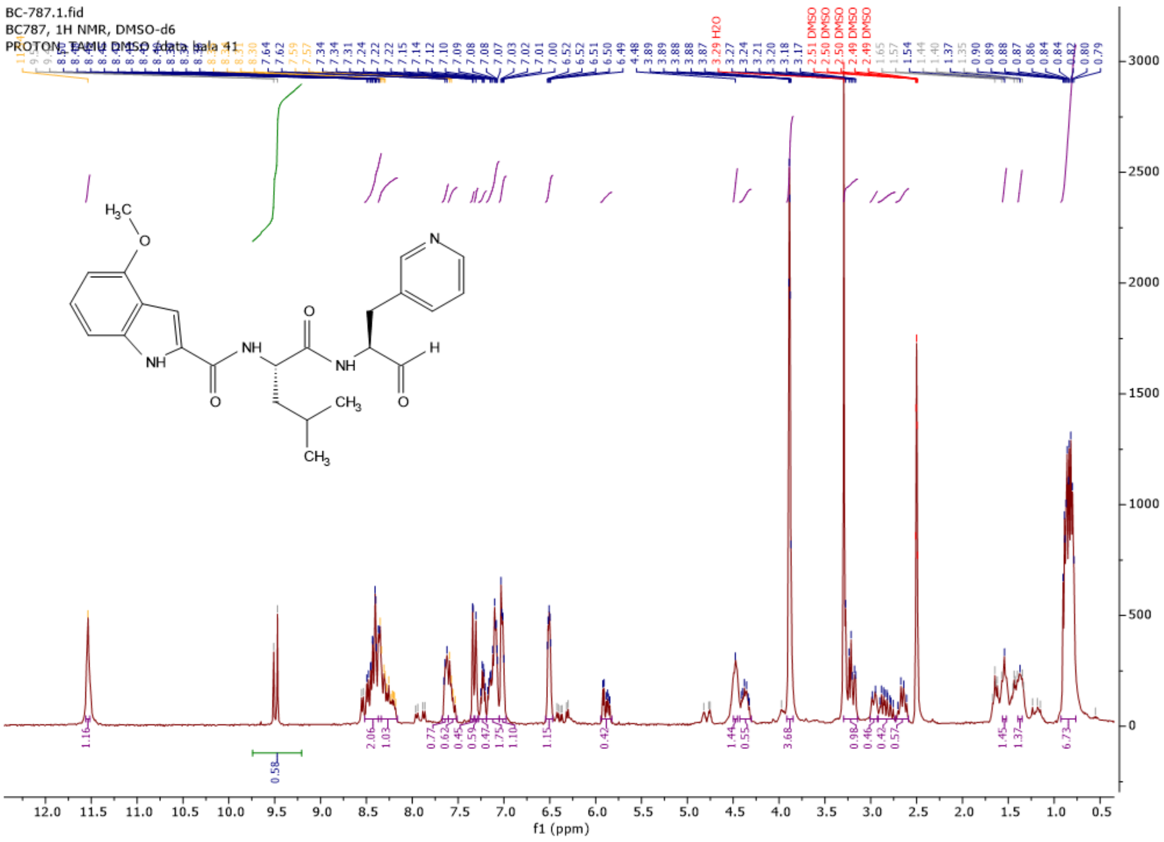
BC-787.1.fid
BC787, 1H NMR, DMSO-d6
3.29 H2O
2.51 DMSO
2.50 DMSO
2.50 DMSO
2.49 DMSO
2.49 DMSO
f1 (ppm)

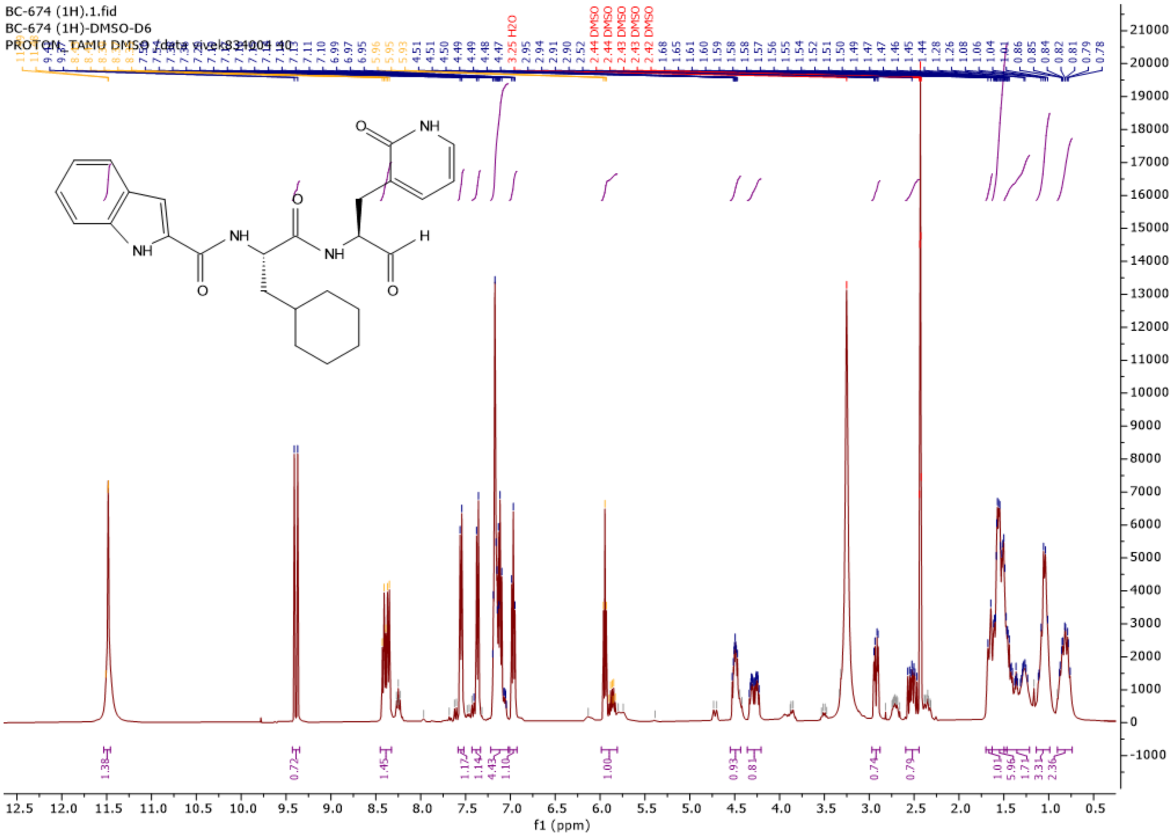
BC-674 (1H).1.fid
BC-674 (1H)-DMSO-D6
3.25 H2O
2.44 DMSO
2.44 DMSO
2.43 DMSO
2.43 DMSO
2.42 DMSO
f1 (ppm)

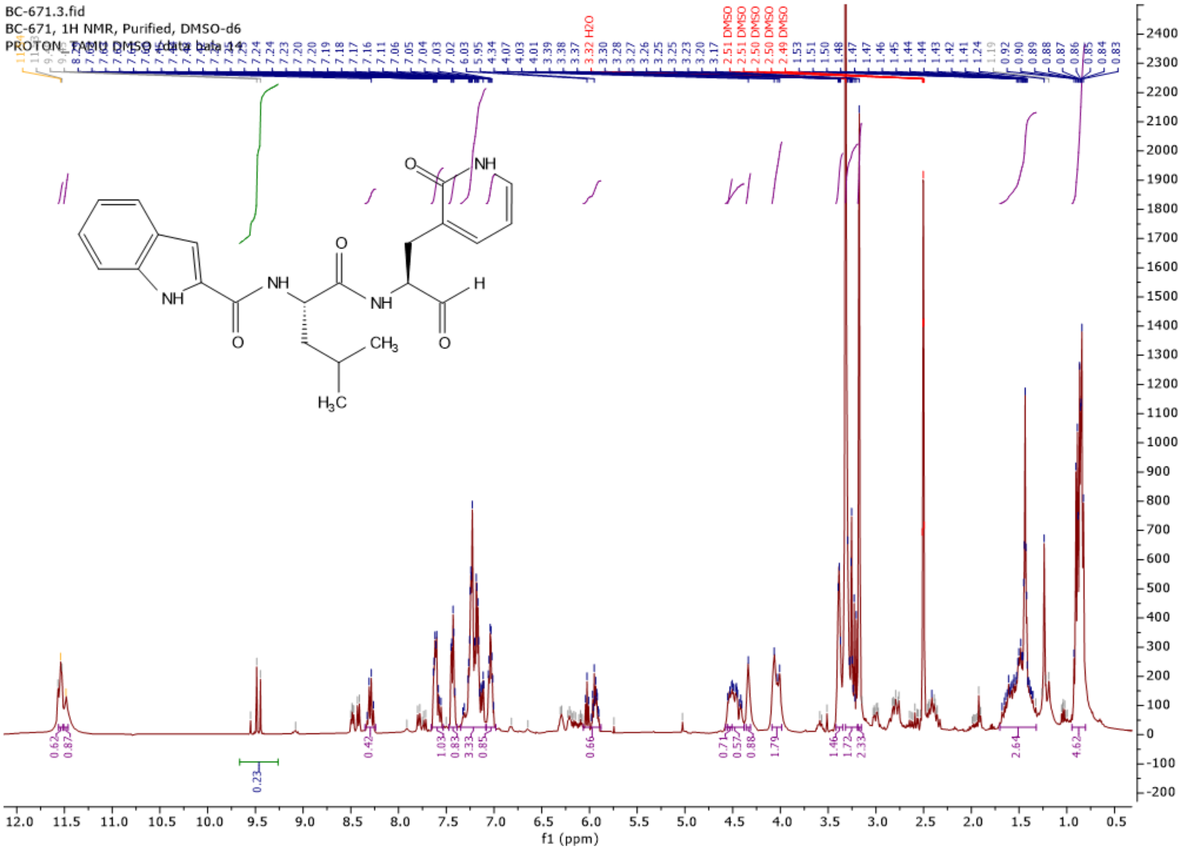

BC-671.3.fid
BC-671, 1H NMR, Purified, DMSO-d6
PROTON_TAMU DMSO {data bak} 14
f1 (ppm)

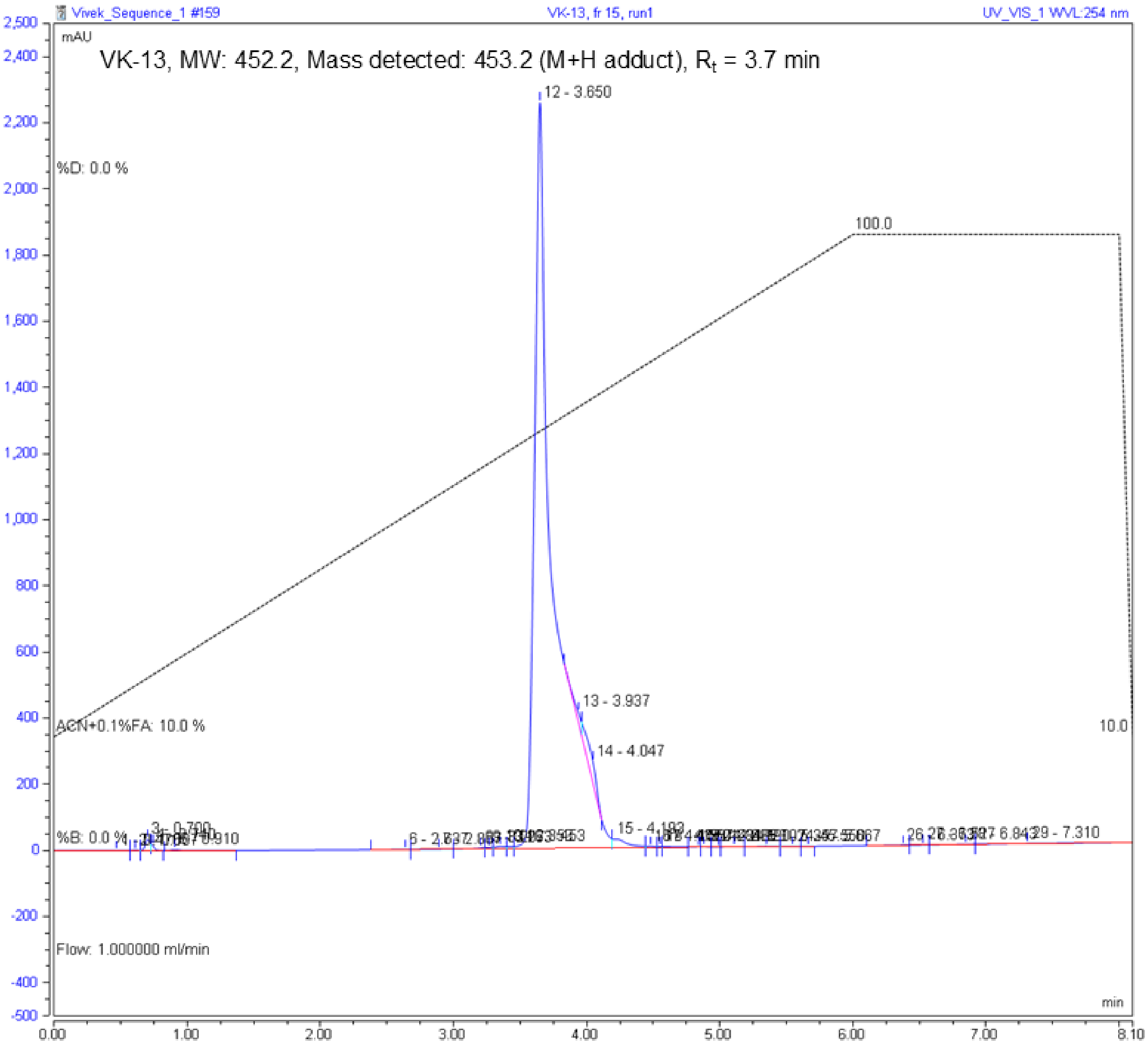
Vivek_Sequence_1 #159
VK-13, fr 15, run1
UV_VIS_1 WVL:254 nm
mAU
VK-13, MW: 452.2, Mass detected: 453.2 (M+H adduct), Rt = 3.7 min
12 - 3.650
%D: 0.0 %
100.0
13 - 3.937
14 - 4.047
ACN+0.1%FA: 10.0 %
10.0
15 - 4.193
29 - 7.310
%B: 0.0 %
Flow: 1.000000 ml/min
min

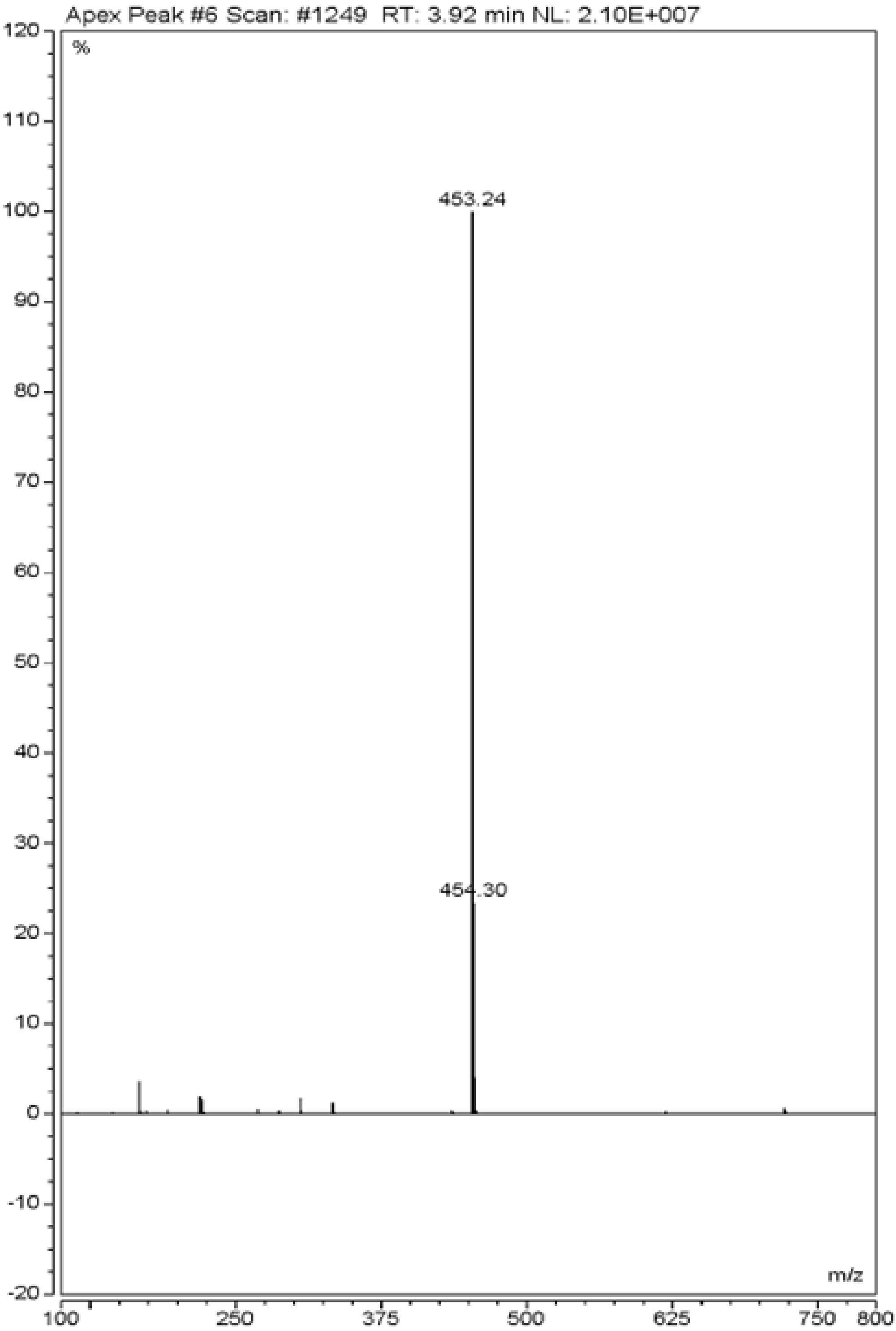

Apex Peak #6 Scan: #1249 RT: 3.92 min NL: 2.10E+007
%
453.24
454.30
m/z

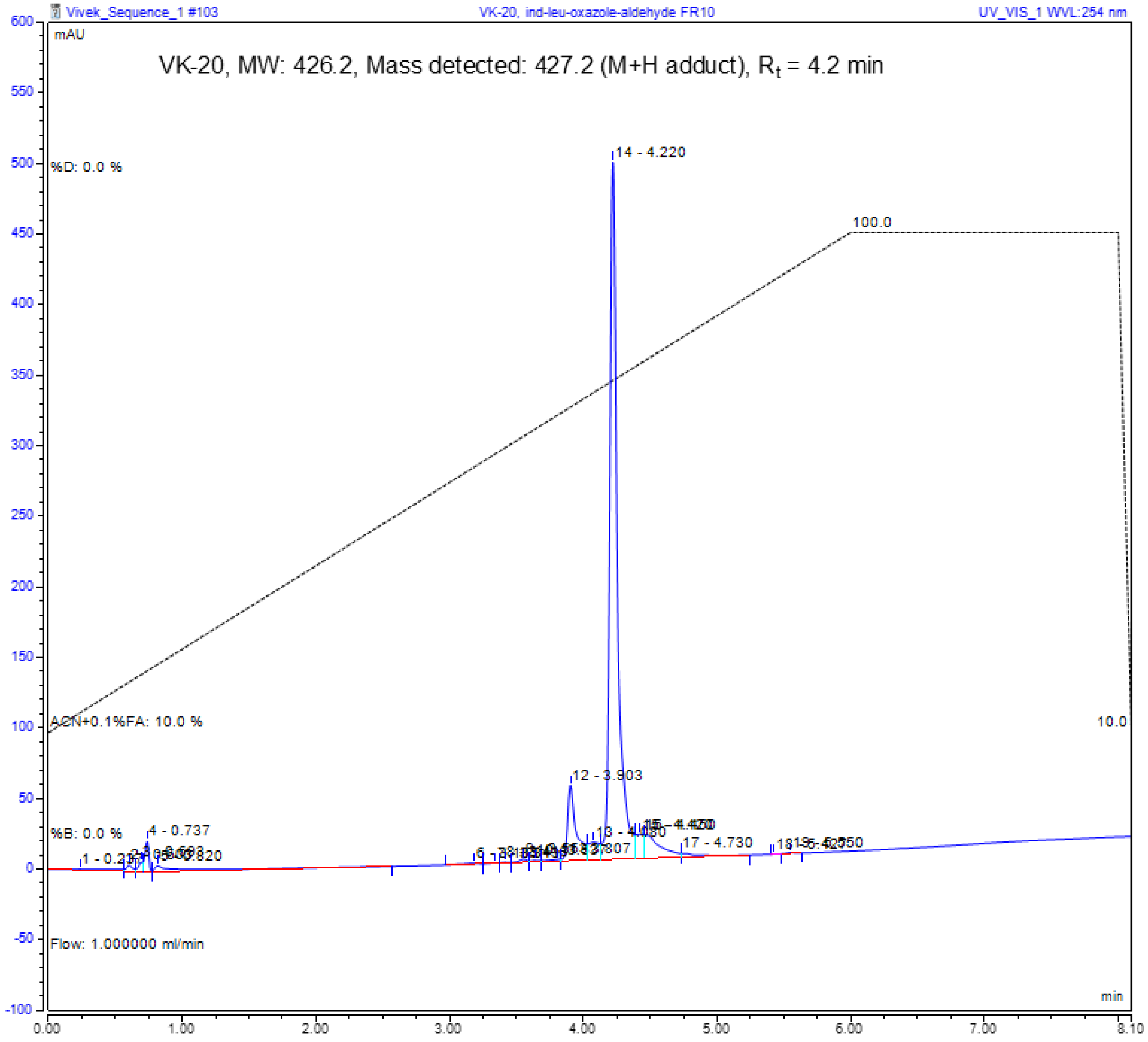

Vivek_Sequence_1 #103
VK-20, ind-leu-oxazole-aldehyde FR10
UV_VIS_1 WVL:254 nm
mAU
VK-20, MW: 426.2, Mass detected: 427.2 (M+H adduct), R_t = 4.2 min
14 - 4.220
100.0
%D: 0.0 %
ACN+0.1%FA: 10.0 %
10.0
12 - 3.903
%B: 0.0 %
4 - 0.737
17 - 4.730
Flow: 1.000000 ml/min
min

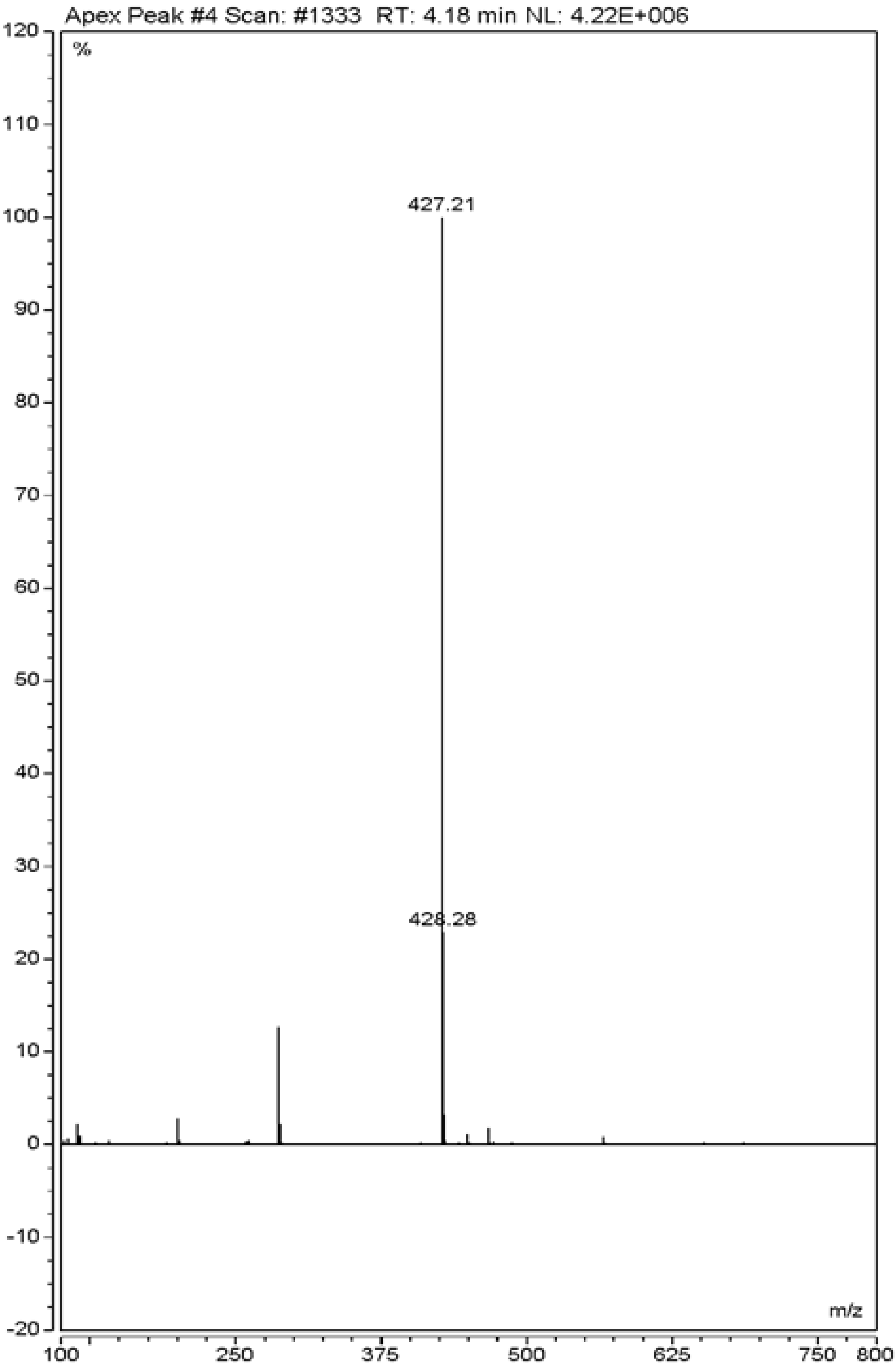
Apex Peak #4 Scan: #1333 RT: 4.18 min NL: 4.22E+006
%
427.21
428.28
m/z
120
110
100
90
80
70
60
50
40
30
20
10
0
-10
-20
100
250
375
500
625
750
800

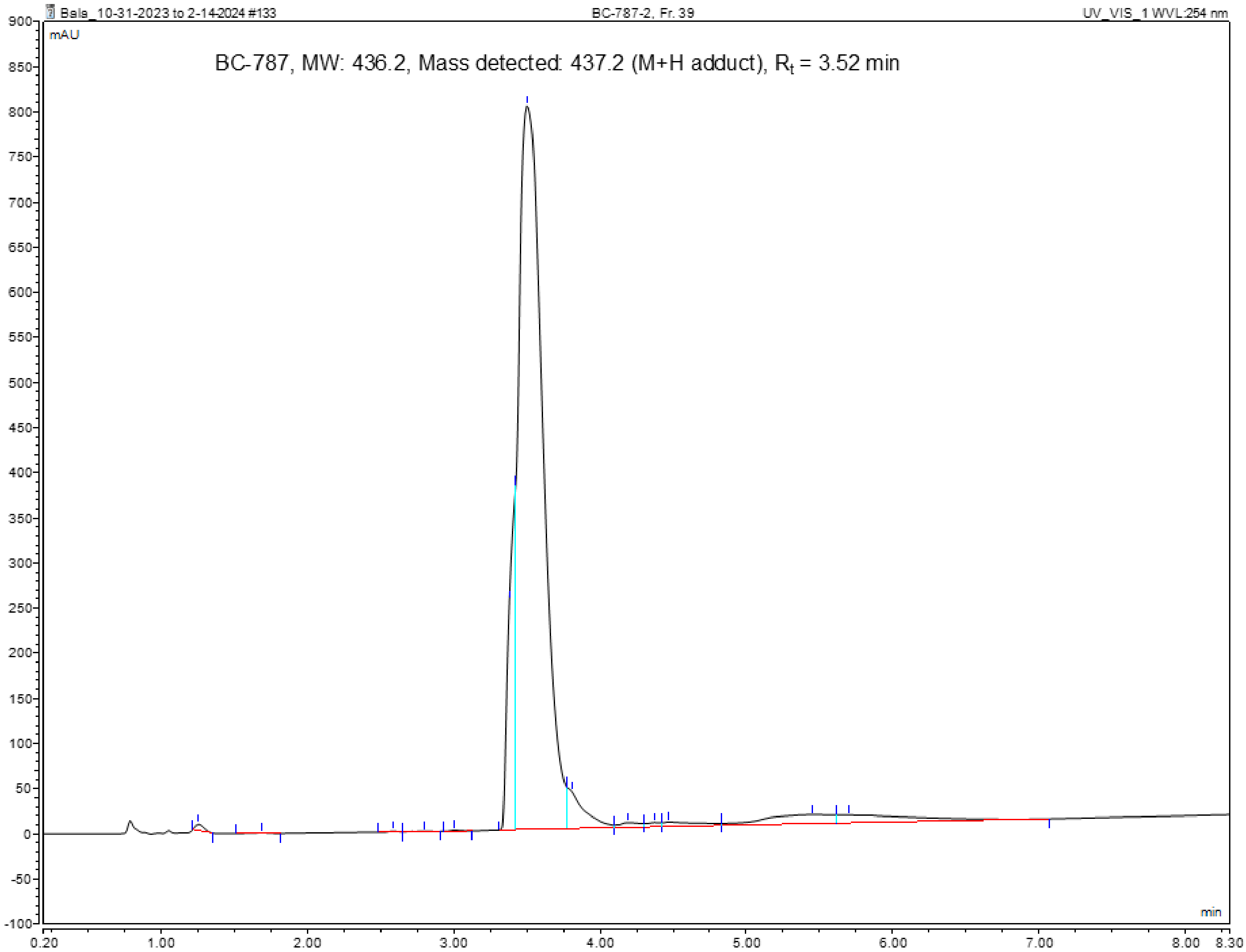
Bala_10-31-2023 to 2-14-2024 #133
BC-787-2, Fr. 39
UV_VIS_1 WVL:254 nm
mAU
BC-787, MW: 436.2, Mass detected: 437.2 (M+H adduct), R_t = 3.52 min
min
900
850
800
750
700
650
600
550
500
450
400
350
300
250
200
150
100
50
0
-50
-100
0.20
1.00
2.00
3.00
4.00
5.00
6.00
7.00
8.00
8.30

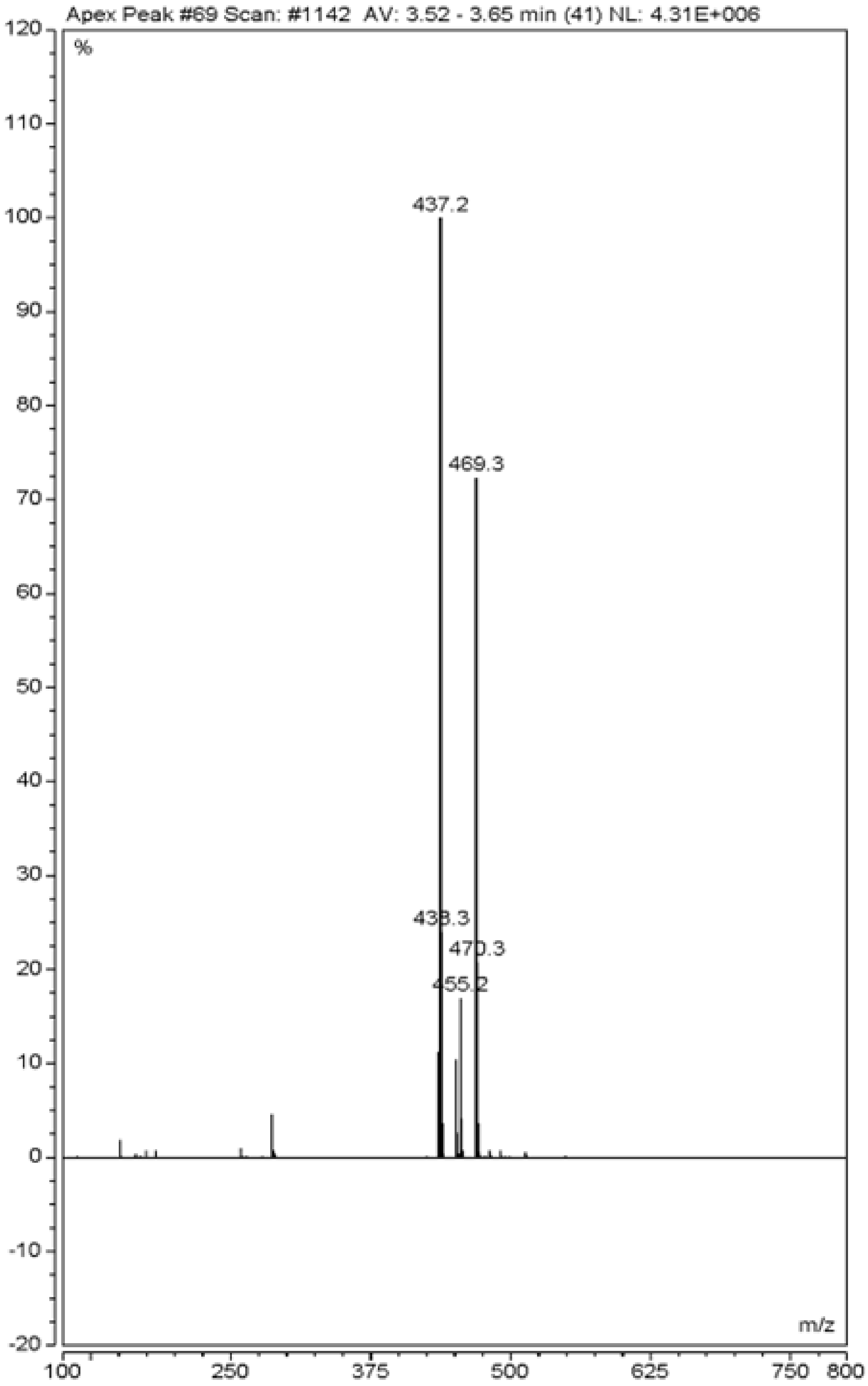

Apex Peak #69 Scan: #1142 AV: 3.52 - 3.65 min (41) NL: 4.31E+006
%
437.2
469.3
438.3
470.3
455.2
m/z
120
110
100
90
80
70
60
50
40
30
20
10
0
-10
-20
100
250
375
500
625
750
800

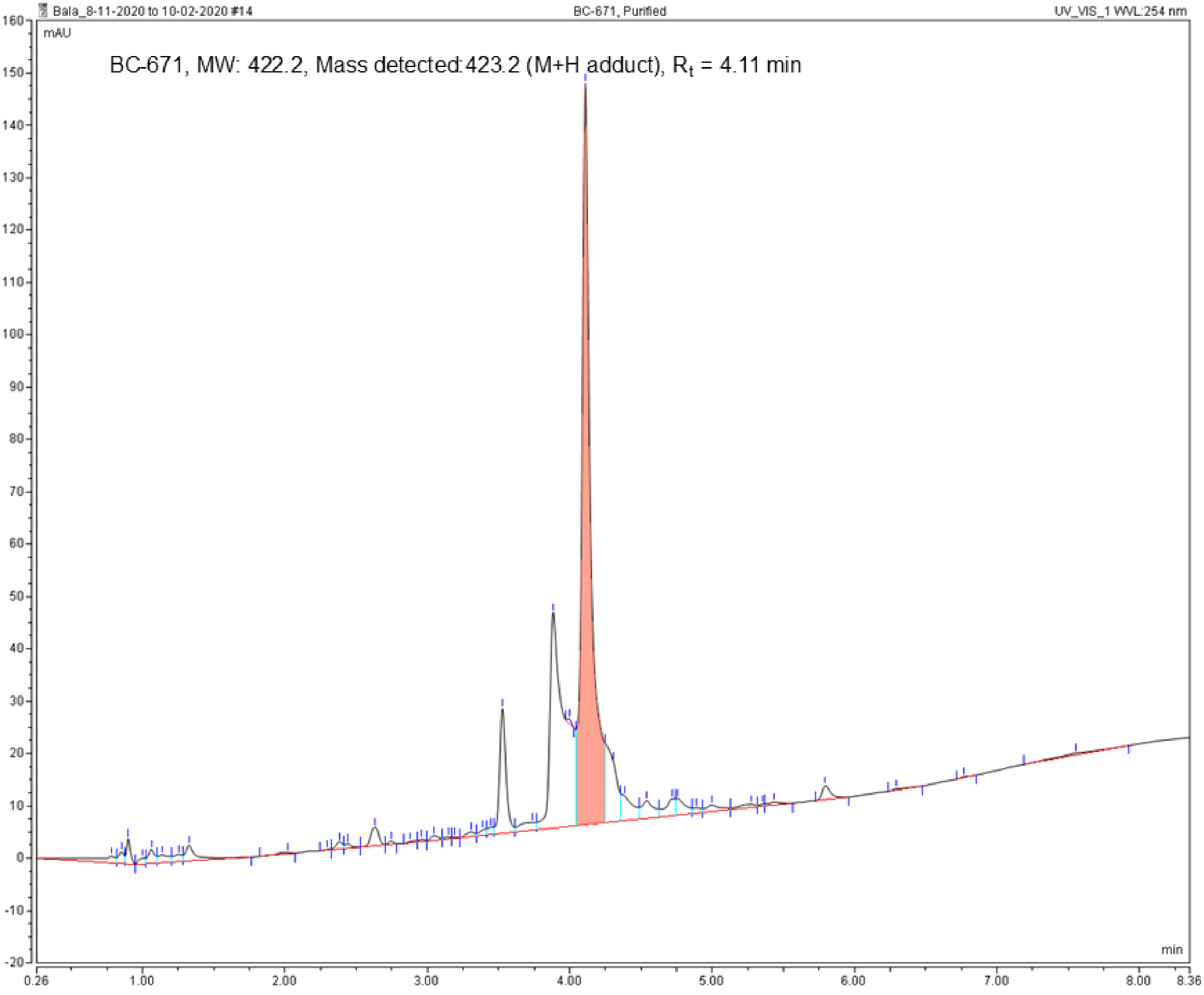
Bala_8-11-2020 to 10-02-2020 #14
BC-671, Purified
UV_VIS_1 WVL:254 nm
mAU
BC-671, MW: 422.2, Mass detected:423.2 (M+H adduct), R_t = 4.11 min
min

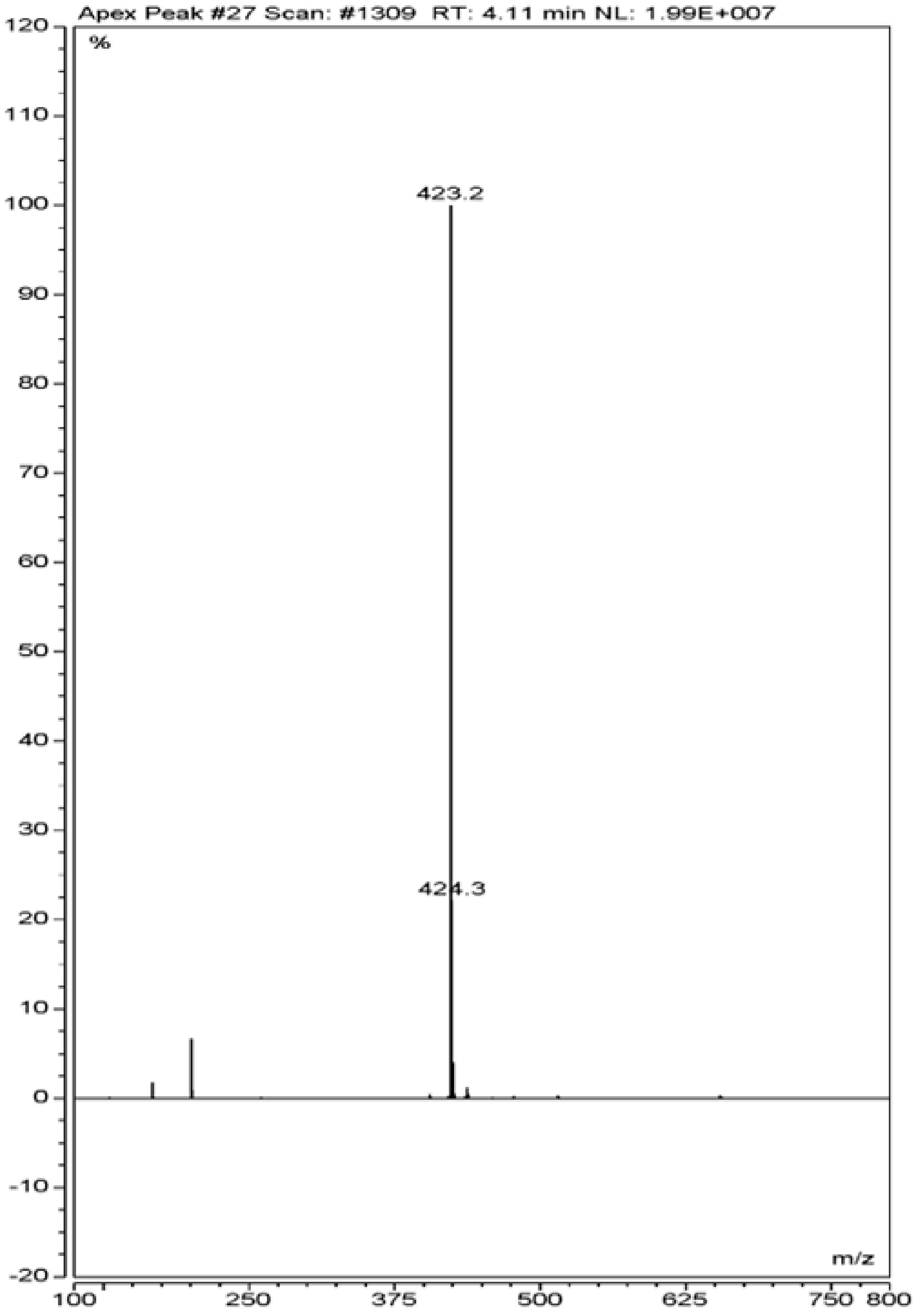

Apex Peak #27 Scan: #1309 RT: 4.11 min NL: 1.99E+007
%
423.2
424.3
m/z
120
110
100
90
80
70
60
50
40
30
20
10
0
-10
-20
100
250
375
500
625
750
800

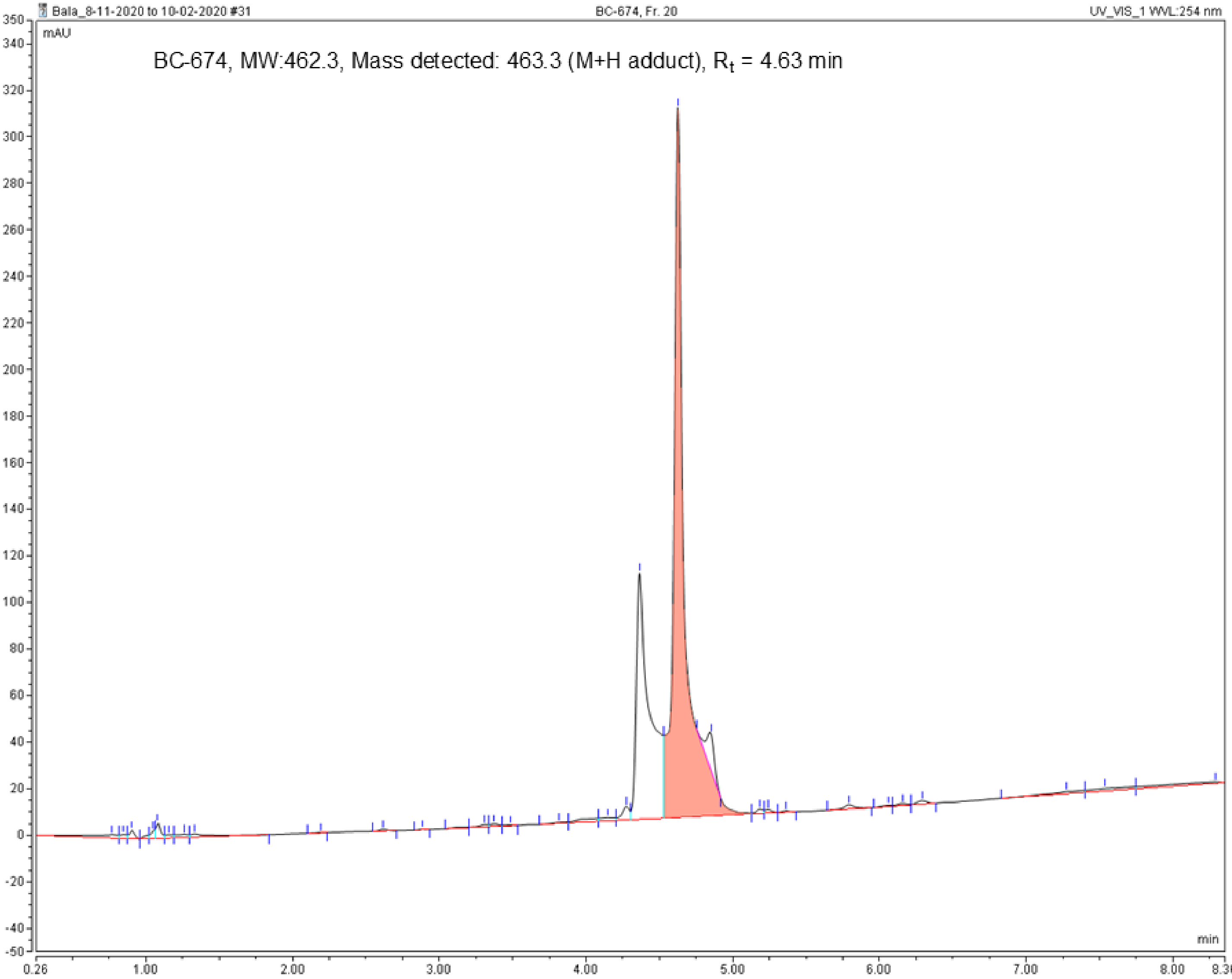

Bala_8-11-2020 to 10-02-2020 #31
BC-674, Fr. 20
UV_VIS_1 WVL:254 nm
mAU
BC-674, MW:462.3, Mass detected: 463.3 (M+H adduct), R$_t$ = 4.63 min
min

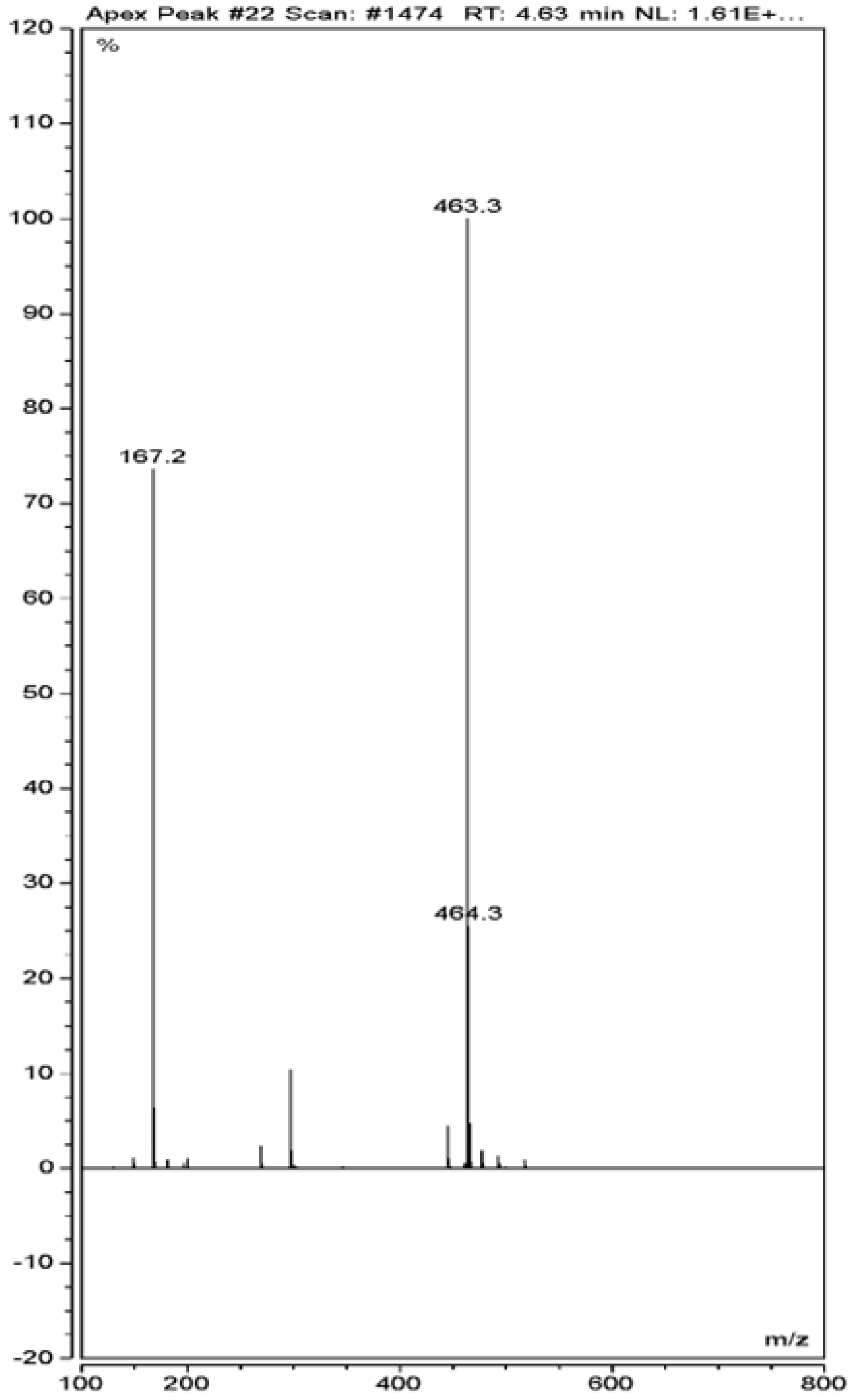

Figure S10. NMR spectra, mass spectra, and HPLC chromatograms of compounds prepared as described in the text.

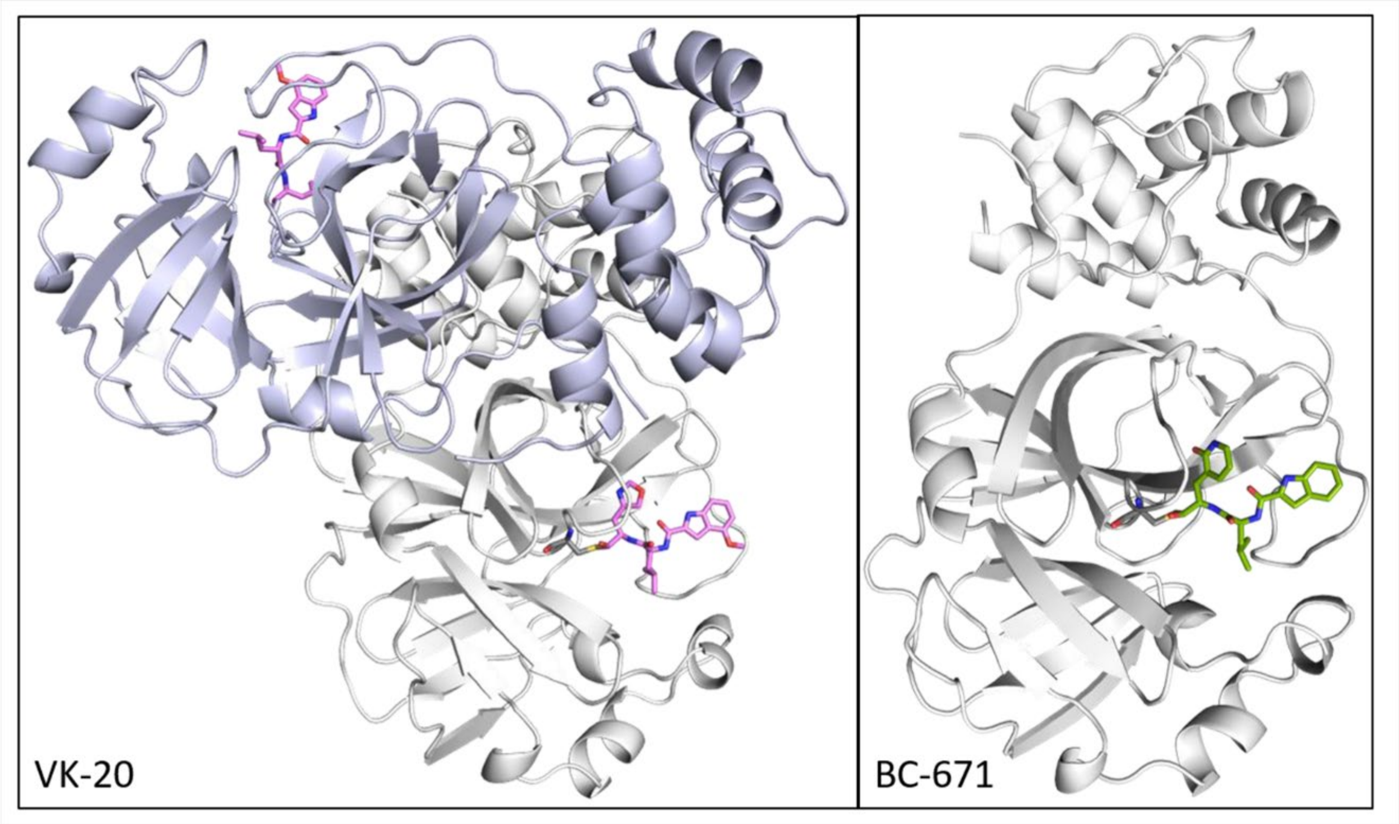

Figure S11. Representative structural overview of 3CL-PR with inhibitors bound. The monomers of the 3CL-PR-**8**: asymmetric unit are shown in lavender and white with the active sites highlighted by Cys_{145} (grey sticks) and the inhibitor (pink). The single monomer of the 3CL-PR-**4** asymmetric unit is shown in white with the active site highlighted by Cys_{145} (grey sticks) and the inhibitor (green).

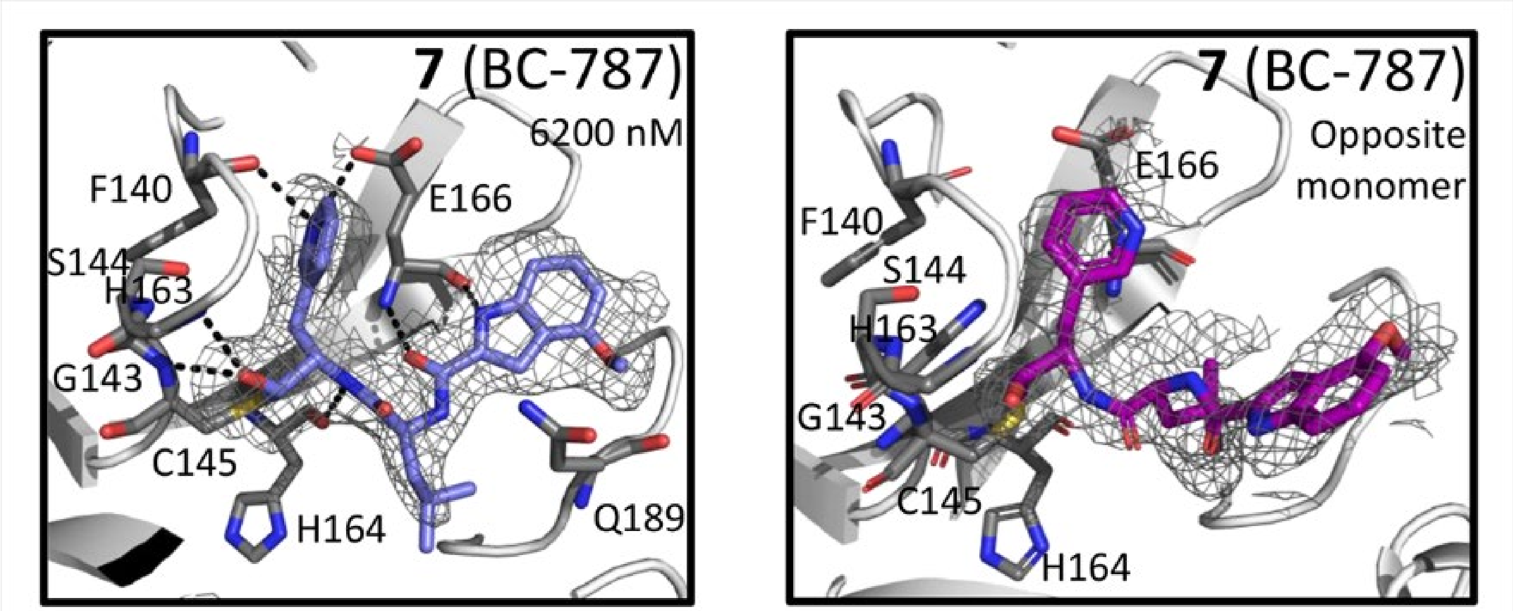

Figure S12. Polder maps contoured at 3σ showing **BC787** bound to each monomer of 3CL-PR of the asymmetric unit.

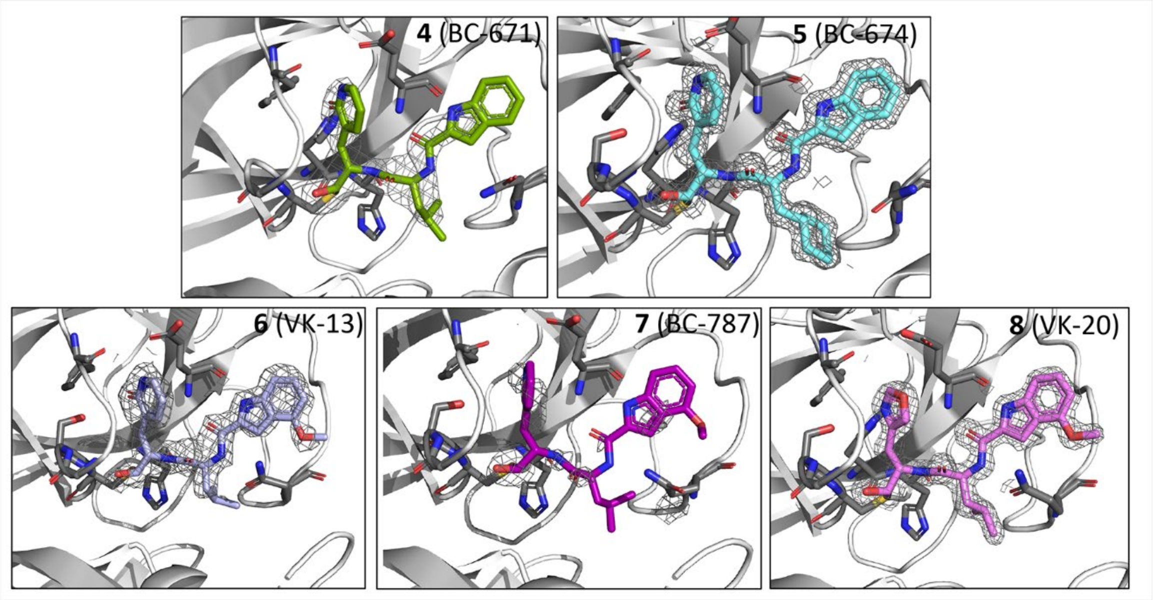

Real-space correlation coefficients (RSCC)

PDB	Inhibitor	RSCC for inhibitor bound in Chain A	RSCC for inhibitor bound in Chain B
9CEK	VK20	0.985	0.990
9CED	VK13	0.924	NA
9CF9	BC787	0.662	0.802
9CEC	BC671	0.921	NA
9CFB	BC674	0.958	0.976

Figure S13. 2Fo-Fc maps contoured at 1.5 σ. Electron density maps are shown for (**Top row**) Inhibitors **BC671** and **BC674** (**Bottom row**): Inhibitors **VK-13 (6**), **BC787**, (**7**) and **VK20** (**8**). The real space correlation coefficients for the inhibitor density are shown in the table below the maps.

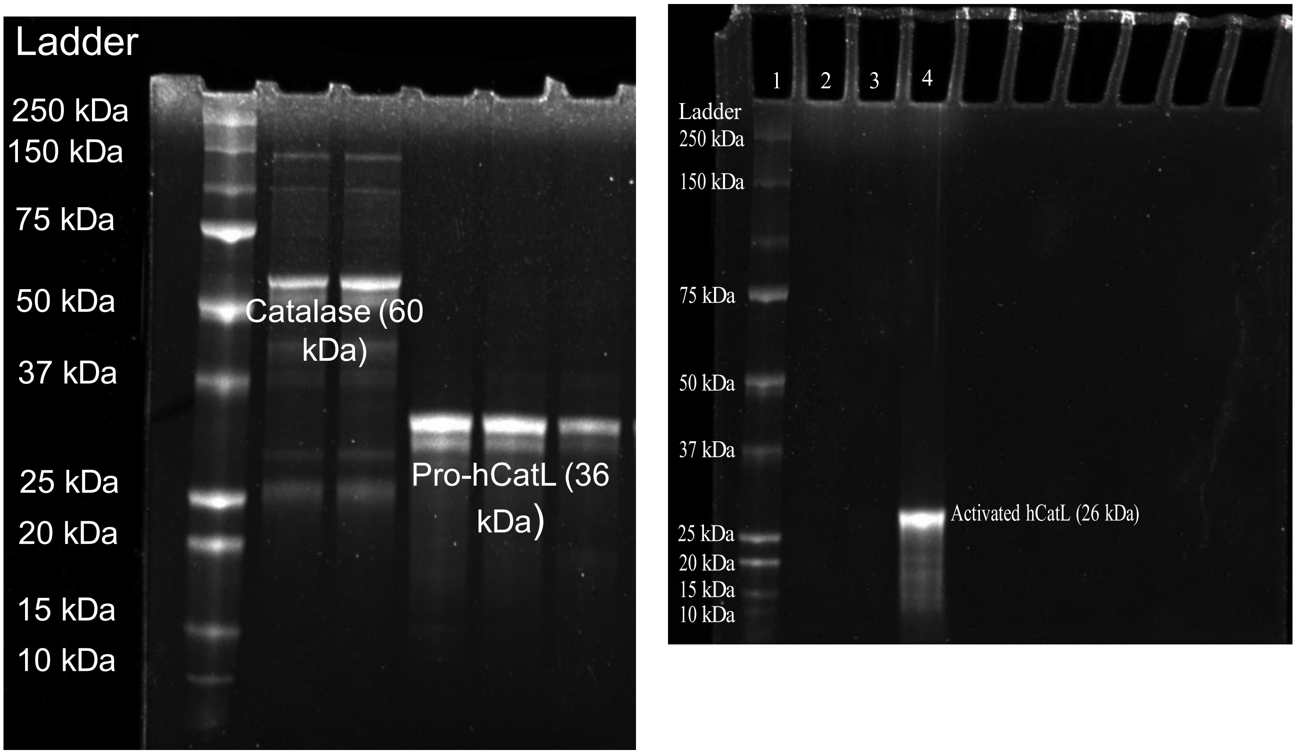

Figure S14. Purification of recombinant human cathepsin L. (**Left**) SDS-PAGE of protein, with fractions obtained from the size exclusion chromatography step of purification procedure. Protein Precision Plus standards (BioRad) and molecular weights, two fractions containing catalase, three fractions containing purified pro-hCatL. (**Right**) (1) Protein Precision Plus standards, (2) concentration flowthrough, (3) concentration flowthrough, (4) activated hCatL

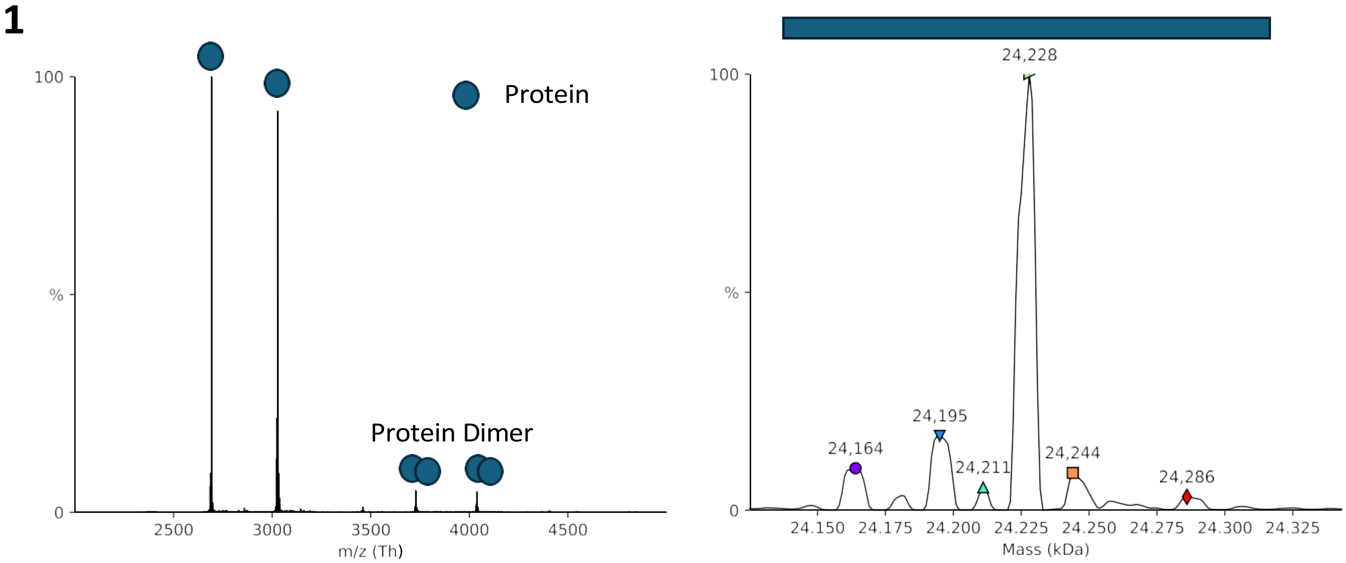

Figure S15. Native mass spectrometry of purified human cathepsin L. Figure (1) Raw mass spectra, (2) Deconvoluted mass spectra.
